# Synthesis of Polypeptides with High-Fidelity Terminal Functionalities under NCA Monomer-Starved Conditions

**DOI:** 10.34133/2021/9826046

**Published:** 2021-11-17

**Authors:** Lei Li, Jie Cen, Wenhao Pan, Yuben Zhang, Xuanxi Leng, Zhengqi Tan, Hao Yin, Shiyong Liu

**Affiliations:** ^1^Hefei National Laboratory for Physical Sciences at the Microscale, Department of Polymer Science and Engineering, School of Chemistry and Materials Science, University of Science and Technology of China, Hefei, Anhui 230026, China; ^2^Mass Spectrometry Lab, Hefei National Laboratory for Physical Sciences at the Microscale, University of Science and Technology of China, Hefei, Anhui 230026, China

## Abstract

Controlled polypeptide synthesis via *α*-amino acid *N*-carboxylic anhydride (NCA) polymerization using conventional primary amine initiators encounters two major obstacles: (i) normal amine mechanism (NAM) and activated monomer mechanism (AMM) coexist due to amine basicity and nucleophilicity and (ii) NCA is notoriously sensitive towards moisture and heat and unstable upon storage. We serendipitously discover that *N*-phenoxycarbonyl-functionalized *α*-amino acid (NPCA), a latent NCA precursor, could be polymerized solely based on NAM with high initiating efficiency by using primary amine hydrochloride as an initiator. The polymerization affords well-defined polypeptides with narrow polydispersity and high-fidelity terminal functionalities, as revealed by the clean set of MALDI-TOF MS patterns. We further demonstrate successful syntheses of random and block copolypeptides, even under open-vessel conditions. Overall, the integration of moisture-insensitive and air-tolerant NPCA precursors with stable primary amine hydrochloride initiators represents a general strategy for controlled synthesis of high-fidelity polypeptides with sophisticated functions.

## 1. Introduction

Synthetic polypeptides are analogues of proteins and exhibit biocompatibility, biodegradability, and stimuli responsiveness [1–4]. Polypeptide synthesis via polymerization of *α*-amino acid *N*-carboxylic anhydride (NCA) is a straightforward approach since its invention by Hermann Leuchs in 1906 [5, 6]. Up to date, controlled synthesis of polypeptides with high-fidelity terminal functionality and narrow polydispersity (*Đ*, *M*_*w*_/*M*_*n*_) still remains a considerable challenge [6, 7]. Although NCA polymerization initiated by primary amines proceeds mainly via the normal amine mechanism (NAM), the activated monomer mechanism (AMM) is also implicated as side reactions due to amine basicity (pKa ~10-12). In addition, primary amine moieties at the terminal of growing chains are also associated with side reactions such as termination by the solvent (e.g., DMF and DMAc) [7–10].

Another challenge originates from the moisture- and heat-sensitive nature of NCA [6, 11]. It is unstable upon storage due to spontaneous initiation by trace water molecules and inadvertently generated amine species, and the preparation of absolutely moisture-free and amine-free NCAs is a formidable task [6, 12]. The structural integrity and stability of primary amine initiators pose another obstacle. Primary amines in the native (i.e., unprotonated) state undergo spontaneous oxidation and carboxylation with CO_2_ [13]. In general, the conjugate acid of primary amines, i.e., ammonium salts, is more stable and could be facilely purified due to loss of both nucleophilicity and basicity.

In order to improve the controllability of NCA polymerization, transition metal [1, 14, 15] and rare earth complexes [16, 17], trimethylsilyl amine and sulfide derivatives [18, 19], and primary ammonium [20–23] have been used as initiators. Recent progresses include hydrogen bonding-assisted organocatalysis [24, 25], LiHMDS-initiated superfast NCA polymerization [26], and superfast NCA polymerization utilizing local cooperative milieu from neighboring *α*-helices [27]. Despite these new developments, controlled NCA polymerization still faces challenges [28]. NCA polymerization is typically conducted at high monomer concentration (0.1-0.5 M) to ensure a reasonable polymerization rate, and this poses the risk of oligomerization via AMM. The use of strong bases (e.g., LiHMDS) is implicated with “carbamate mechanism,” in addition to AMM [29, 30]. Furthermore, NCA polymerization kinetics and polypeptide products were typically characterized with FT-IR, NMR, and GPC techniques. For previous reports of NCA polymerization using MALDI-TOF MS technique, the presence of impurity peaks associated with side reactions is clearly evident, indicating uncertain fidelity for polypeptide chain terminals [7, 9, 16–19, 25, 26, 31, 32]. To address the moisture and heat-sensitive nature of NCA monomers [6, 10, 11], activated urethane (i.e., *N*-aryloxycarbonyl) derivative of *α*-amino acid, which is stable and much easier to handle, has been utilized to in situ generate NCA at elevated temperature for polypeptide synthesis [33–40]. However, the polymerization process is still associated with multiple side reactions (AMM, solvent-mediated initiation and termination, and cyclization, etc.) when amine initiators are used.

**Figure 1:**
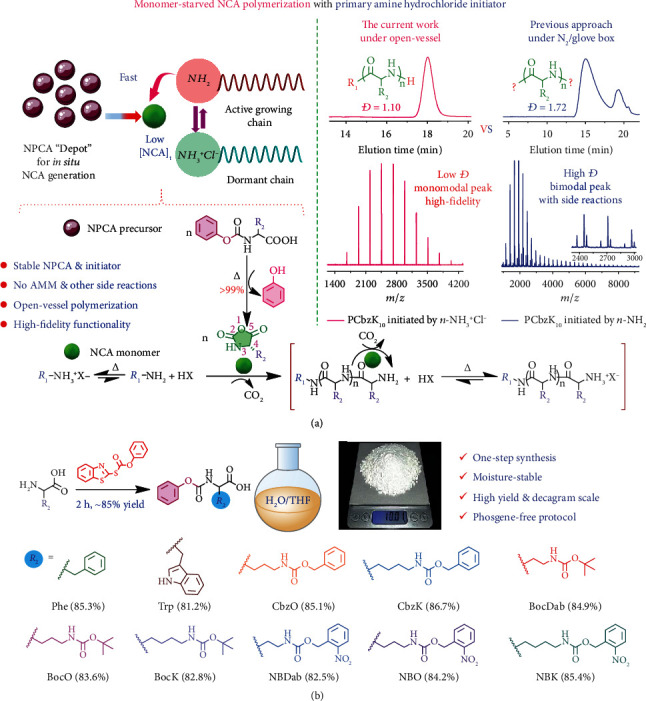
Controlled polypeptide synthesis via NPCA polymerization using primary amine hydrochloride initiator. (a) Schematics of controlled synthesis of polypeptides with predetermined MW, low polydispersity, and well-defined chain terminal functionalities using *N*-phenoxycarbonyl-functionalized *α*-amino acid (NPCA) as NCA monomer precursor and structurally stable primary amine hydrochloride as initiator. Upon heating, moisture-insensitive NPCA in situ transforms into NCA. Primary amine initiators and terminal amine moieties of growing chains shuttle between dormant state (protonated) and activated state (deprotonated), which could prominently diminish undesired side reactions associated with conventional NCA polymerization. The controllability of polymerization is assisted by low NCA concentration throughout the polymerization process (i.e., monomer-starved condition) and NCA anion-capturing capability of released phenol upon NPCA transformation into NCA, thus inhibiting polymerization via the AMM pathway. All these features lead to controlled synthesis of (co) polypeptides with diverse chain topologies and high-fidelity terminal functionalities. The polypeptide synthesis could be facilely conducted under open-vessel condition, as exhibited by the monomodal GPC elution peak and clean set of MALDI-TOF MS pattern recorded for PCbzK_10_ as a typical example. For comparison, GPC and MALDI-TOF MS data of PCbzK_10_ synthesized under glovebox condition using *n*-BuNH_2_ initiator are also shown. (b) Schematics of decagram scale synthesis of moisture-stable NPCA precursors in high yield using (*S*)-1,3-benzothiazol-2-yl-*O*-phenylthiocarbonate as the key intermediate. NPCAs are stable upon storage under open air, which solves the moisture-sensitive issue associated with conventional NCA monomer.

During the course of synthesizing unnatural polypeptides with triggered degradation features [41, 42], we serendipitously discovered that *N*-phenoxycarbonyl-functionalized *α*-amino acid (NPCA), the latent NCA precursor [33, 34], could be polymerized solely based on NAM with high initiating efficiency by using primary amine hydrochloride as the initiator. Well-defined (co) polypeptides with narrow polydispersity and high-fidelity terminal functionalities could be obtained, as revealed by the clean set of MALDI-TOF mass peaks (Figure 1). The use of the primary amine hydrochloride initiator allows for the shuttling of amine moieties of growing chain termini between dormant state (protonated) and activated state (deprotonated), diminishing undesired side reactions associated with amine nucleophilicity and polymerization via AMM. In-depth mechanistic studies revealed that the polymerization is conducted under NCA monomer-starved condition due to in situ NCA generation from latent NPCA precursor and fast consumption of the former. This could effectively inhibit side reactions associated with NCA instability and AMM-relevant NCA oligomerization. The released phenol during transformation of NPCA into NCA could also help suppress the AMM pathway, as phenol has slightly lower pKa compared to NCA anions [43].

## 2. Results and Discussion

### 2.1. Facile Synthesis of Moisture-Stable NPCA Precursors

The strategy of in situ generation and polymerization of NCA monomers could be dated back to 1951 [44]. Ehler and Orgel [45] utilized *N*-imidazolyl-(1)-carbonyl functionalized amino acids as NCA precursors for polypeptide synthesis in aqueous media. NPCA derivatives were also prepared by masking *α*-amino functionality with phenyl chloroformate or diphenyl carbonate (DPC), which suffers from prolonged reaction time, unsatisfactory yields, and incompatibility with acid-labile functionalities [34, 36]. After screening diverse range of protocols and reagents, we found that (*S*)-1,3-benzothiazol-2-yl-*O*-phenylthiocarbonate could serve as a potent reagent to mask *α*-amino functionality and generate corresponding NPCA precursors (Figure 1(b)) [46]. The improved protocols could be completed within ~2 h with a yield up to ~85%. As no hydrogen chloride was evolved during reaction, this approach is also compatible with acid-labile functionalities (e.g., Boc protecting group shown in Figure 1(b)) [34]. To demonstrate the generality and feasibility, a variety of functionalized NPCA precursors based on natural and unnatural amino acids including lysine (K), ornithine (O), and 2,4-diaminobutyric acid (Dab) were synthesized. Pendant amine moieties were protected with *tert*-butoxycarbonyl, benzyloxycarbonyl, and *o*-nitrobenzyloxycarbonyl functionalities (Schemes S1-S4; Figure 1(b)). Detailed procedures and structural characterization data of NBDab, NBO, NBK, BocDab, BocK, BocO, CbzO, and CbzK are described in Supplementary Materials (Schemes S1-S4; Figures S2-S8). Moreover, tryptophan and phenylalanine-based NPCA derivatives, Trp and Phe, were also synthesized (Scheme S4). Compared to moisture-sensitive and storage instability issues relevant to NCA [6, 11], NPCAs are moisture-insensitive and stable upon storage under open air [33–38, 47]. Thus, NPCAs could be purified by recrystallization in an open vessel without generating any impurities, rendering it convenient for scale-up. For example, NBO precursor could be prepared as white crystals at ~10 g scale from a single batch (Figure 1(b) and Figure S4).

### 2.2. Insights into NPCA Polymerization Kinetics Initiated by Primary Amine Hydrochloride

When *n*-BuNH_2_ was used at first to initiate NBO polymerization, GPC traces of obtained polypeptides are bimodal, with MWs deviating from target ones. *Đ* of PNBO ([*M*]_0_/[*I*]_0_ = 5-100) is quite broad (1.35-1.89) (Figure S9). In order to solve this problem, we used *n*-BuNH_2_ initiator in combination with 10 eq. acetic acid to enhance polymerization controllability [39, 40]. Unfortunately, the resultant polypeptides still exhibited bimodal distribution with *Đ* in the range of 1.36-1.69 for a series of NPCAs (Figure S10). Next, small molecule primary ammonium salt, *n*-butylamine hydrochloride (*n*-BuNH_3_^+^Cl^−^) [20–23], was used as the initiator for NPCA polymerization; unexpectedly, we found that NBO precursor could be polymerized in a controlled manner at 70°C and [*M*]_0_/[*I*]_0_ feed ratio of 80, with *Đ* being 1.14 (Table S1, entry 6).

**Figure 2:**
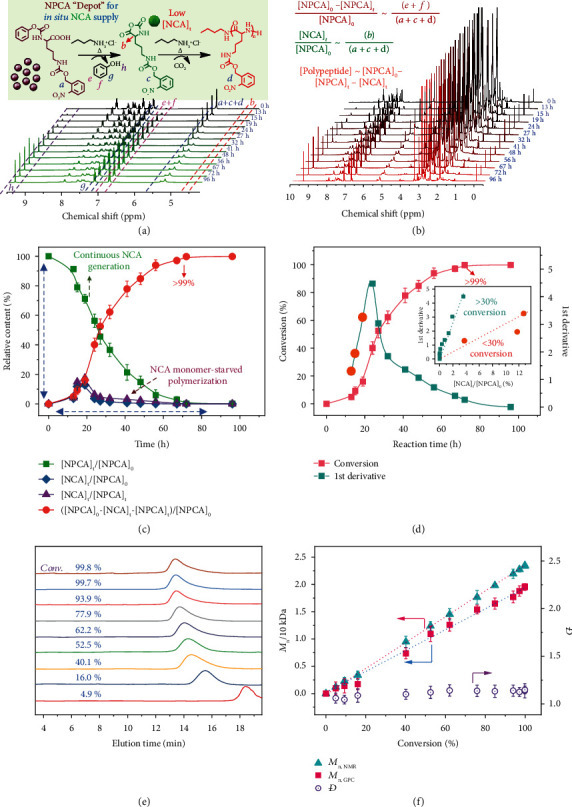
Kinetics of NPCA polymerization initiated by *n*-BuNH_3_^+^Cl^−^. (a) Time-dependent evolution of NMR spectra with corresponding peak assignments of NPCA precursor, in situ generated NCA monomer, and polypeptide. (b) Real-time ^1^H NMR spectra recorded for polymerization kinetics of NBO precursor initiated by *n*-BuNH_3_^+^Cl^−^ ([*M*]_0_/[*I*]_0_ = 80, [*M*]_0_ = 0.25 M, DMAc, 70°C). (c) Time-dependent evolution of relative contents of [NPCA]_*t*_, [NCA]_*t*_, and polypeptide. (d) Evolution of the extent of polypeptide formation (conversion) and corresponding 1^st^ derivatives; the inset shows the plot of 1^st^ derivatives versus relative contents of NCA_*t*_. (e) GPC elution traces during NBO polymerization. (f) *M*_*n*,NMR_, *M*_*n*,GPC_, and *Đ* (*M*_*w*_/*M*_*n*_) recorded during NBO polymerization. Data are presented as the mean ± SD (*n* = 3).

Upon heating to 70°C, NPCA precursor undergoes cyclization and converts in situ into NCA (Figure 1), which was evidenced from the evolution of ^1^H NMR signals characteristic of both NCA and released phenol (Figures 2(a) and 2(b)). Note that phenol does not directly initiate NCA polymerization [48–50]. Variations of instantaneous NCA intermediate ([NCA]_*t*_/[NPCA]_0_) and residual NPCA ([NPCA]_*t*_/[NPCA]_0_) are shown in Figure 2(c) and Figure S11. Remarkably, [NCA]_*t*_ maintained at a relatively low concentration throughout the entire polymerization process, and the highest concentration (~12.8% relative to [NPCA]_0_) was reached at ~19 h. Relative NCA concentrations remained in the range of 0-1.9% from 24 h to 72 h (Figure 2(c)). These results implied that the NCA generation process was the rate-limiting step, and the polymerization was conducted under NCA monomer-starved condition at both intermediate and final stages. Aiming for a target DP of 80, the extent of polypeptide formation was >99% within ~72 h, and the conversion exceeded 95% within ~60 h (Figure 2(d)).

The 1^st^ derivative of polypeptide conversion *vs.* time plot reflected relative rates of polymerization. Intriguingly, the variation of the 1^st^ derivative with relative [NCA]_*t*_ revealed two distinct stages of polymerization kinetics (inset in Figure 2(d)). The polymerization was relatively sluggish when the conversion was lower than 30%, which is in agreement with the gradual increase of [NCA]_*t*_. At elevated conversion, the polymerization rate rapidly increased. We ascribed the rate increase to the formation of polypeptide secondary structures, which also occurs for conventional NCA polymerization [51]. The polymerization process was also monitored by GPC (Figures 2(e) and 2(f)). With the increase of NPCA consumption extent, MWs of formed polypeptides gradually increased, exhibiting quite narrow polydispersities (*Đ* ~1.04-1.14). The linear correlation between *M*_*n*,GPC_ and *M*_*n*,NMR_ with conversions verified the controlled living feature of *n*-BuNH_3_^+^Cl^−^ initiated NPCA polymerization up to >99% conversion (Figure 2(f)). Moreover, the DPs of obtained polypeptides agreed quite well with theoretical values (Table S1).

### 2.3. Synthesis of High-Fidelity Polypeptides via NPCA Polymerization Initiated by *n*-BuNH_3_^+^Cl^−^

To investigate the effects of counter anions on NPCA polymerizations, a series of primary ammonium salts with varying counter anions including Cl^−^, Br^−^, BF_4_^−^, PF_6_^−^, and ClO_4_^−^ were used as initiators (Figures S12-S15). NBO polymerization was conducted in DMAc at 70°C and a fixed [*M*]_0_/[*I*]_0_ feed ratio of 100 (Figure S16(a); Table S2). All primary ammonium salts could successfully initiate NBO polymerization, although MW and *Đ* of the final polypeptide products varied. Specifically, *n*-BuNH_3_^+^Cl^−^ initiator resulted in almost complete conversion (>99%) with the narrowest *Đ* (~1.13; Table S2). *n*-BuNH_3_^+^Br^−^ initiator afforded a moderate *Đ* (1.24), but with much lower monomer conversion (<60%). In the absence of *n*-BuNH_3_^+^Cl^−^ initiator, self-polymerization of CbzK precursor at elevated temperature was completely inhibited by the introduction of HCl, revealing the sole role of amine moiety as initiating species (Figure S17). These results also indicated that in DMAc, chloride ion cannot directly initiate NPCA polymerization; moreover, the HCl component in *n*-BuNH_3_^+^Cl^−^ initiator could effectively inhibit inadvertent oligomerization and other side reactions.

For primary ammonium initiators with more bulky and nonnucleophilic BF_4_^−^, PF_6_^−^, and ClO_4_^−^ counteranions, NBO polymerization at 70°C and [*M*]_0_/[*I*]_0_ = 100 all reached >90% conversion after 72 h, with *Đ* being 1.22, 1.35, and 1.27, respectively (Figure S16(a); Table S2). This might be partially due to the intrinsic instability of HBF_4_ and HPF_6_ (moisture sensitivity, spontaneous degradation in contact with glassware surface) and thermal decomposition at elevated temperatures. Previously, neopentylammonium tetrafluoroborate was used to initiate polymerization of the NCA monomer of *N*-*ε*-benzyloxycarbonyl-*L*-lysine in DMF at 40°C; DMF GPC analysis revealed an increase of *Đ* from 1.15 to 1.70 when the average DP of PCbzK decreased from 196 to 24 [20, 52]. We therefore compared CbzK polymerizations using *n*-BuNH_3_^+^Cl^−^, *n*-BuNH_3_^+^BF_4_^−^, and *n*-BuNH_2_ as initiators. At a fixed [*M*]_0_/[*I*]_0_ ratio of 30, only *n*-BuNH_3_^+^Cl^−^ initiator afforded monomodal elution peak, whereas both *n*-BuNH_3_^+^BF_4_^−^ and *n*-BuNH_2_ initiators led to bimodal or even multimodal GPC elution traces (Figure S18). For CbzK polymerization using *n*-BuNH_3_^+^BF_4_^−^ initiator at even lower [*M*]_0_/[*I*]_0_ ratios (10 and 20), multimodal GPC elution traces were also obtained (Figure S19). These results revealed that counteranions of primary ammonium initiators played crucial roles in regulating NPCA polymerizations [20]. We further conducted NPCA polymerization at varying temperatures (30-70°C) using *n*-BuNH_3_^+^Cl^−^ as the initiator (Figure S16(b); Table S2). When the polymerization was performed at 30°C and 40°C (Table S2, entries 1-2), no polypeptide was formed, indicating that low temperature is insufficient to shift the amine protonation-deprotonation equilibrium. However, the polymerization could smoothly proceed at temperatures > 50°C, with a polymerization temperature of 70°C being the optimized condition in terms of both *Đ* and target DPs.

**Figure 3:**
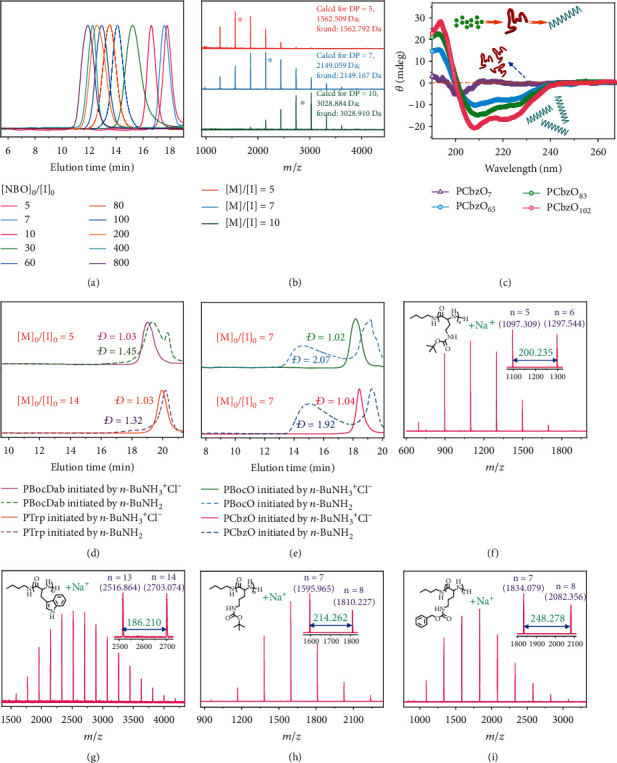
Characterization of NPCA polymerization products using primary amine and primary amine hydrochloride initiators. (a) GPC traces of PNBO polypeptides synthesized at varying [*M*]_0_/[*I*]_0_ ratios using *n*-BuNH_3_^+^Cl^−^ as the initiator. (b) MALDI-TOF MS spectra recorded for PNBO polypeptides synthesized using *n*-BuNH_3_^+^Cl^−^ initiator at [*M*]_0_/[*I*]_0_ ratios of 5, 7, and 10, respectively. (c) Circular dichroism (CD) spectra recorded for PCbzO_7_, PCbzO_65_, PCbzO_83_, and PCbzO_102_ in HFIP (20°C, 0.05 mg/mL). (d, e) Comparison of GPC elution traces of polypeptides, PBocDab, PTrp, PBocO, and PCbzO, synthesized via NPCA polymerization at varying [*M*]_0_/[*I*]_0_ ratios using *n*-BuNH_3_^+^Cl^−^ and *n*-BuNH_2_ as initiators, respectively. (f–i) MALDI-TOF MS spectra recorded for (f) PBocDab_5_, (g) PTrp_13_, (h) PBocO_7_, and (i) PCbzO_7_ synthesized using *n*-BuNH_3_^+^Cl^−^ as the initiator. All polymerizations were conducted in DMAc at [*M*]_0_ = 0.25 M and 70°C.

We further explored the synthesis of PNBO polypeptides with varying DPs by adjusting [*M*]_0_/[*I*]_0_ feed ratios in the range of 5-800 using *n*-BuNH_3_^+^Cl^−^ as the initiator (Table S1). The DPs were generally consistent with feed ratios, and all GPC traces of resultant polypeptides were monodisperse with relatively narrow *Đ* (Figure 3(a)). Remarkably, MALDI-TOF MS data of PNBO_5_, PNBO_7_, and PNBO_10_ revealed only one single set of peaks (Figure 3(b) and Figure S20; Table S1, entries 1-3), and the mass peak interval (~293.275 Da) agreed with the NBO repeating unit. During the process of PCbzK_35_ synthesis, MALDI-TOF MS, instead of GPC, was used to directly monitor polypeptide chain growth at varying NPCA conversions. We could observe the clear shift of MS patterns with the maximum MS peak increasing to higher MWs. Most importantly, only one set of MS peaks corresponding to the desired polypeptide was detected at all intermediate conversions (Figure S21). Note that this is unprecedented in the field of polypeptide synthesis via NCA polymerizations [2]. The high fidelity of terminal functionalities for polypeptides synthesized using *n*-BuNH_3_^+^Cl^−^ initiator was applicable to a diverse range of NPCAs including NBK and NBDab, as revealed by GPC and MALDI-TOF MS data (Figures S22-S26; Table S1). These results confirmed that primary amine hydrochloride-initiated NPCA polymerizations strictly follow the NAM pathway (Figure 1). It is worthy of noting that in previous studies concerning NPCA polymerization using primary amine initiators, MALDI-TOF MS data revealed the presence of shoulder MS peaks corresponding to impurities generated by the AMM pathway and amine-incurred side reactions, exhibiting broader *Đ* and even multimodal GPC elution peaks [34, 36, 38, 53].

*n*-BuNH_3_^+^Cl^−^ was further used to initiate the polymerization of other types of NPCAs including CbzO, Trp, BocDab, and BocO in DMAc at 70°C (Table S1). All GPC traces were monomodal with narrow *Đ* values (1.02-1.16), and *M*_*n*,NMR_ values of resultant polypeptides were close to theoretical ones. PCbzO_65_, PCbzO_83_, and PCbzO_102_ in hexafluoroisopropanol (HFIP) displayed circular dichroism (CD) signals characteristics of an *α*-helix secondary structure (Figure 3(c)). In contrast, the CD spectrum of PCbzO_7_ revealed typical random coil conformation in HFIP. Besides, characteristic ATR-FT-IR amide peaks at 1651 cm^−1^ (amide I band) and 1543 cm^−1^ (amide II band) for PCbzO confirmed the formation of peptide backbone linkages (Figure S27c).

Figures 3(d) and 3(e) compare GPC traces of PBocDab, PTrp, PBocO, and PCbzO synthesized via polymerization of corresponding NPCAs using *n*-BuNH_3_^+^Cl^−^ and *n*-BuNH_2_ as initiators, respectively. All polypeptides synthesized using *n*-BuNH_3_^+^Cl^−^ initiator exhibited monomodal GPC traces with narrow *Đ* (<1.05), and the DPs are close to predetermined feed ratios (Table S1). MALDI-TOF MS analysis further confirmed that polypeptides with *n*-BuNH_3_^+^Cl^−^ initiation exhibited narrow MW distribution with a clean set of MS patterns corresponding to [*M*_*n*_ + Na]^+^, and no impurities corresponding to side reactions could be discerned (Figures 3(f)–3(i)). In contrast, the use of *n*-BuNH_2_ initiator, its combination with acetic acid, and *n*-BuNH_3_^+^BF_4_^−^ initiator all afforded polypeptides with multimodal MW distributions, as evidenced from both GPC and MALDI-TOF MS characterization data (Figures 3(d) and 3(e) and Figures S28-S31).

Based on the above GPC and MALDI-TOF MS data (Tables S1 and S2; Figure 3 and Figures S9-S31), we could conclude that NPCA polymerizations at 70°C in DMAc using primary amine hydrochloride initiator are ideal for controlled synthesis of well-defined polypeptides ranging from low to high DPs. Though tremendous progress has been made towards high DP polypeptides via NCA polymerization [20, 26, 51], the synthesis of low DP polypeptides with well-defined terminal functionalities encounters major challenges [52, 54]. First, the initial consumption of primary amine initiator lags behind the NCA polymerization process (i.e., chain growth) due to the former possessing higher pKa compared to peptidic terminal amines [13], leading to preferential protonation of primary amine initiator by in situ generated carbamic acid moiety of growing chains. Second, above a critical chain length, secondary structure formation of growing polypeptide chains will exhibit a dramatically enhanced polymerization rate compared to polypeptides with lower DPs, leading to broader or even bimodal MW distributions [54]. Finally, secondary structure formation of polypeptides in the low DP range also poses difficulties for GPC analysis; secondary structure formation leads to chain collapse and contributes to shoulder or even bimodal GPC elution traces [52]. In the current work, primary amine hydrochloride initiators not only prohibit most of the side reactions relevant to basicity (e.g., AMM pathway) and nucleophilicity of both initiator and peptidic amines, the protonation-deprotonation equilibrium will also favor the initial fast consumption of initiator amines due to its higher nucleophilicity compared to peptidic amine moieties. We surmise that at elevated temperatures, the pKa discrepancy between initiator and peptidic amines will be lower. This could help solve the issue of the sluggish initiation step of primary amine-initiated NCA polymerization due to the higher pKa of amine initiators compared to peptidic amines. Note that in conventional NCA polymerizations, the terminal carbamic acid moiety of growing chains tends to protonate both initiator and peptidic amines, especially at early stages. The preferential protonation of the former leads to problematic slow initiation. At intermediate and later stages, the proton level will decrease due to consumption of NCA monomers and CO_2_ release. Side reactions associated with both amine basicity and nucleophilicity will then emerge, leading to multimodal GPC elution traces and impurity MALDI peaks.

In the current study, GPC analysis was conducted in DMF solvent using two TSKgel columns (G3000 H_HR_ and G5000 H_HR_) with MW ranges of 1-4000 kDa against polystyrene standards. Thus, MW and *Đ* values of low DP polypeptides might not be accurate and, the latter tends to be overestimated. We are fully aware that for an ideal living polymerization, the theoretical limit of *Đ* is equal to 1/DP+1. To further probe this issue, MALDI-TOF MS characterization, which reports structural parameters at the chain level rather than the ensemble level (i.e., in GPC), was extensively utilized to characterize these low DP polypeptides. As demonstrated in Table 1, Figure 3, and Figures S28-S31, all oligopeptides with DPs in the range of 5-13 exhibit narrow *Đ*_MALDI_ values comparable to those obtained from GPC (*Đ*_GPC_). Although the mass discrimination issue will complicate MALDI analysis of polymers with high MW and broad *Đ*, the polymers analysed in this study are of low MW and narrow *Đ*; the associated errors should be fairly small [55]. We thus conclude that these results verified the highly controlled nature for polypeptide synthesis via NPCA polymerization in DMAc at 70°C using a primary amine hydrochloride initiator.

Figure S29c directly compares the CD spectra of PCbzK_7_ synthesized using either *n*-BuNH_3_^+^Cl^−^ or *n*-BuNH_2_ as the initiator. It is intriguing to note that the former exhibits typical random coil conformation whereas the latter exhibits characteristic *α*-helix signals. These results are consistent with MALDI-TOF MS data (Figure S30a), and PCbzK_7_ synthesized using *n*-BuNH_2_ initiator exhibits bimodal MW distribution. From the high MW shoulder peaked at ~4 kDa, we could also deduce that the critical DP for secondary structure formation is ~15 for PCbzK. Note that this critical DP corresponds to the GPC elution time at ~16 min. Closer examination of Figure 3(a) and Figure S16a reveals that if the GPC elution profile spans across this critical region, apparent broadening and tailing are clearly evident, implying gradual instead of abrupt transition from random coil to *α*-helix with ascending polypeptide DPs [52].

### 2.4. Insights into NPCA Polymerization Mechanisms

Aiming to probe relevant side reactions associated with NPCA polymerization initiated with *n*-BuNH_2_, we analysed polypeptide products by MALDI-TOF MS (Figure S32). As-synthesized PTrp consisted of target sequences via NAM (i) and chain end modification with NPCA-derived isocyanate derivative (ii and iii, Figure S32(a)) [56]. In addition to isocyanate-relevant side products, impurities originating from the reaction of terminal amine with DMAc solvent (ii, Figure S32(b)) are also observed for PCbzO, which is similar to chain termination side reaction for conventional NCA polymerization using DMF as solvent [7, 9, 32]. Note that during MALDI-TOF MS characterization, terminal amine also reacts with the MALDI matrix, *trans*-2-[3-(4-*tert*-butylphenyl)-2-methyl-2-propenylidene] malononitrile (DCTB; iii, Figure S32(b)) [57]. Carboxyl moiety at the *C*-terminal (iii, Figure S32(c)) could be attributed to intermolecular polycondensation reaction [33, 34]. Moreover, side products corresponding to *C*-terminal NCA ring-modified PBocO (ii, Figure S32(c)) and PBocDab (iv, Figure S32(d)) and cyclic PBocDab (ii, Figure S32(d)) could also be discerned. Note that these impurities are ascribed to the AMM polymerization pathway, whereas the formation of cyclic polypeptide side products was likely due to the backbiting of terminal amine onto the *5*-carbonyl moiety of conjugated NCA at the *C*-terminal [10, 58]. For NPCA polymerization initiated with *n*-BuNH_2_ in combination with acetic acid and HBF_4_, MALDI-TOF MS analysis also revealed the presence of impurities due to similar side reactions described above (Figures S33 and S34).

Figure S1 compared NPCA polymerization mechanisms using primary amine and primary amine hydrochloride initiators. Clean MALDI-TOF MS patterns in Figures 3(f)–3(i) and Figures S20-S26 revealed that primary amine hydrochloride-initiated NPCA polymerization follows the NAM pathway without any discernible side reactions (Figure 1). In contrast, both conventional NCA polymerization and primary amine-initiated NPCA polymerization are plagued with various side reactions, leading to uncertainties in chain terminal functionalities (Figure S1) [33–38]. As shown in Figure 2, NPCA polymerization proceeds under NCA monomer-starved conditions, especially at intermediate and late stages. Upon heating, in situ generated NCA from NPCA precursor was initiated by primary amines derived from the protonation-deprotonation equilibrium for primary ammonium initiators. Thus, NCA concentration remains to be quite low throughout the polymerization process, thereby eliminating side reactions associated with NCA instability. The decrease of effective NCA concentration will also help eliminate AMM-relevant NCA oligomerization.

During chain growth, newly generated NCAs undergo nucleophilic substitution reactions with peptidic terminal amines; note that this process is more preferred due to polypeptide secondary structure formation (Figure 2(d)). Amine moieties of primary ammonium initiators and growing peptidic chains shuttle between dormant state (protonated) and activated state (deprotonated), thus diminishing undesired amine basicity-relevant side reactions (Figure 1 and Figure S1). According to FT-IR spectra of *n*-BuNH_3_^+^Cl^−^ in DMAc at varying temperatures (Figure S35), the amine protonation-deprotonation equilibrium more favor the inert protonated state even at 70°C. This feature is advantageous to effectively prohibit side reactions relevant to nucleophilicity of both amine and chloride ions. As *n*-BuNH_3_^+^Cl^−^ initiator possesses higher pKa (~11.1 in DMSO) compared to terminal peptidic amine (~8.4 in DMSO), the initiation step will be much faster than the chain growth step due to the higher amine nucleophilicity of the former. Meanwhile, the presence of amine protonation-deprotonation equilibrium also effectively suppresses NCA anion (NCA^−^) formation, which is the active intermediate associated with the AMM pathway [12, 23]. We also propose that released phenol (pKa ~18 in DMSO) during NPCA transformation into NCA could help suppress NCA^−^ formation; in organic solvents, the pKa of phenol is comparable to that of NCA/NCA^−^ ionization equilibrium [43, 59]. Furthermore, the reversible protonation of peptidic terminal amines also excluded the occurrence of nucleophilic side reactions with solvents (DMAc) and isocyanate intermediates and backbiting-relevant intramolecular cyclization (see Figure S32 for details).

For NPCA polymerization initiated with *n*-BuNH_2_, although carboxyl moiety in NPCA precursor could reversibly protonate *n*-BuNH_2_ at the initial stage, the transformation of NPCA into NCA and phenol will gradually consume carboxyl moieties. Note that the released phenol (pKa ~18 in DMSO) loses the capability of protonating peptidic amines (pKa ~8.4 in DMSO). Thus, at intermediate and late stages, available carboxyl moieties are insufficient to render reversible amine protonation. Previously, *n*-BuNH_2_ in combination with an excess of acetic acid was also used for NPCA polymerization [39, 40]. Considering that acetic acid (pKa ~12 in DMSO) is incapable of efficient protonation of both *n*-BuNH_2_ and peptidic terminal amines, the observed less controllability of NPCA polymerization could be expected (Figures S10, S28, and S33). Overall, primary amine hydrochloride-initiated NPCA polymerization at elevated temperatures provides a reliable strategy towards the synthesis of well-defined polypeptides with controlled MW, narrow polydispersity, and high-fidelity terminal functionalities (Figure 1).

### 2.5. Open-Vessel NPCA Polymerization Initiated by Primary Amine Hydrochloride Initiator

**Figure 4:**
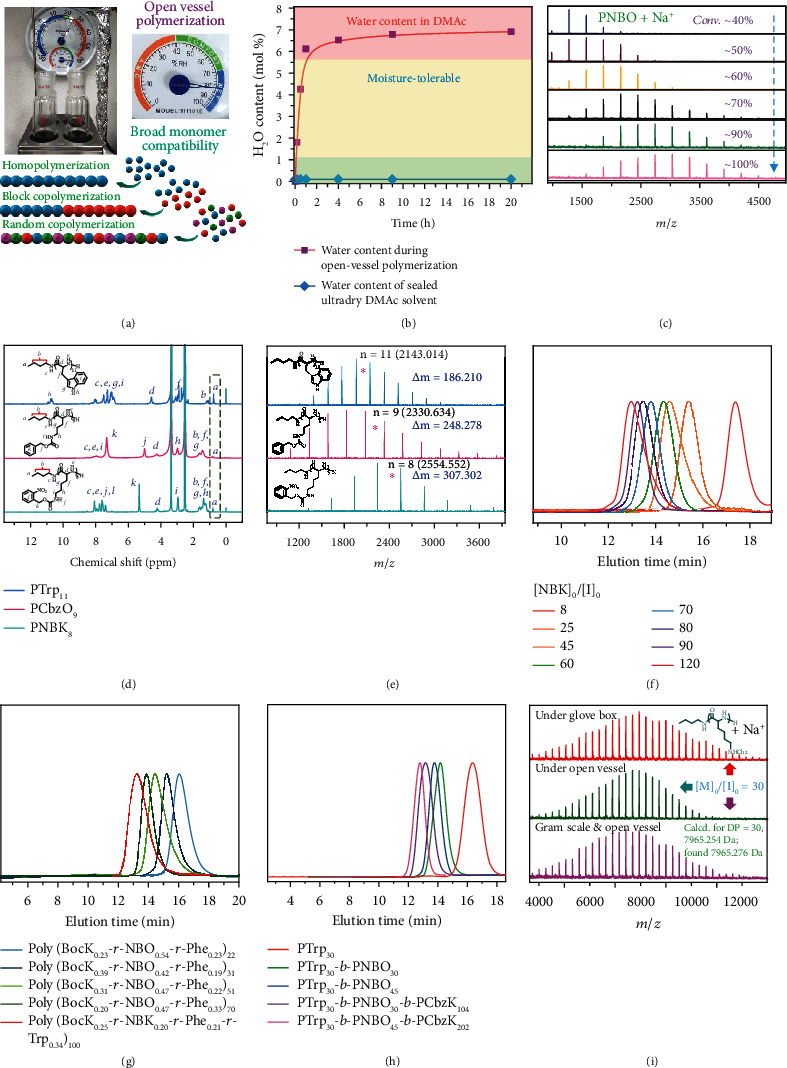
Open-vessel homopolymerization, random, and block copolymerization of NPCA precursors initiated by *n*-BuNH_3_^+^Cl^−^. (a) Schematics of open-vessel homopolymerization, block copolymerization, and random copolymerization of NPCA precursors initiated by *n*-BuNH_3_^+^Cl^−^. (b) Evolution of water content during open-vessel NBO polymerization in DMAc initiated by *n*-BuNH_3_^+^Cl^−^ at [*M*]_0_/[*I*]_0_ = 10. (c) Evolution of MALDI-TOF MS spectra recorded at varying conversions during open-vessel NBO polymerization in DMAc initiated by *n*-BuNH_3_^+^Cl^−^ at [*M*]_0_/[*I*]_0_ = 10, revealing the moisture-tolerant feature. (d) ^1^H NMR spectra recorded for PTrp_11_, PCbzO_9_, and PNBK_8_. The dotted region shows characteristic signals of initiator residues at *C*-terminal, indicating NAM mechanism under open-vessel condition. (e) MALDI-TOF MS spectra recorded for PTrp_11_ ([*M*]_0_/[*I*]_0_ = 12), PCbzO_9_ ([*M*]_0_/[*I*]_0_ = 9), and PNBK_8_ ([*M*]_0_/[*I*]_0_ = 8) synthesized under open-vessel condition. (f) GPC elution traces recorded for PNBK synthesized at varying [*M*]_0_/[*I*]_0_ feed ratios under open-vessel condition. (g) GPC elution traces recorded for P(BocK_*x*_‐*co*‐NBO_*y*_‐*co*‐Phe_1‐*x*‐*y*_)_*n*_ and P(BocK_*x*_‐*co*‐NBK_*y*_‐*co*‐Phe_*z*_‐*co*‐Trp_1‐*x*‐*y*‐*z*_)_*n*_ random copolypeptides synthesized at varying [*M*]_0_/[*I*]_0_ feed ratios under open air. (h) GPC elution traces recorded for diblock and triblock copolypeptides with varying block lengths synthesized under open air. (i) MALDI-TOF MS spectra recorded for PCbzK polypeptides synthesized under inert atmosphere and open air and at gram and tens of milligram scales, respectively. All polymerizations were conducted at [*M*]_0_ = 0.25 M in DMAc and 70°C.

Conventional NCA polymerizations are conducted under inert gas protection using anhydrous solvents and reagents, despite recent progresses of superfast NCA polymerizations [25, 26, 60]. Considering the stability of both primary amine hydrochloride initiators and NPCA precursors and the fact that NPCA polymerization mainly proceeds under NCA monomer-starved condition, we envisaged that it might be feasible to directly conduct NPCA polymerization under open-vessel conditions (Figure 4(a)). Under an environmental humidity of >80%, the water content in DMAc increased and stabilized to ~7 mol% after ~4 h under open-vessel condition (Figures 4(a) and 4(b) and Figure S36). Taking NBO polymerization as an example ([*M*]_0_/[*I*]_0_ = 10), clean MALDI-TOF MS patterns and evolution into higher MWs with increasing NBO conversions revealed the robustness of open-vessel NPCA polymerization initiated by *n*-BuNH_3_^+^Cl^−^ (Figure 4(c)). GPC and ^1^H NMR analysis also revealed controllability of the open-vessel polymerization process (Figure S37), which is also applicable to other types of NPCAs at varying [*M*]_0_/[*I*]_0_ ratios (Figures 4(d)–4(f) and Figures S38-S43; Table 1).

Controlled NPCA polymerization under open-vessel condition could be interpreted according to rationales listed below. First, the pKa of residual water (up to ~7 mol%) in DMAc solvent is estimated to be ~31.4; thus, it is a weaker acid compared to phenol, HCl, and carboxyl moiety of NPCA monomer in the polymerization medium (i.e., DMAc). Note that at the final stage of polymerization, the concentration of released phenol could be up to 0.25 M. This explains the compatibility of residual water with NPCA polymerization under open-vessel condition. On the other hand, though NCA is moisture-sensitive, NPCA polymerization is conducted under NCA monomer-starved condition (Figures 2(c) and 2(d)). The NPCA precursor is stable under open-vessel condition (Figure 1), and newly generated NCA will be quickly consumed. This feature could thus solve water-sensitivity issue associated with NCA monomer during conventional NCA polymerizations [6, 11]. We further verified this feature by using primary amine to directly initiate NPCA polymerization under open-vessel condition, revealing the absence of impurity peaks derived from water molecules (Figure S44).

In addition to homopolymerization, copolymerization of NPCA precursors were also conducted under open-vessel condition. P(BocK_*x*_‐*co*‐NBO_*y*_‐*co*‐Phe_1‐*x*‐*y*_)_*n*_ and P(BocK_*x*_‐*co*‐NBK_*y*_‐*co*‐Phe_*z*_‐*co*‐Trp_1‐*x*‐*y*‐*z*_)_*n*_ random copolypeptides were successfully synthesized, with compositions and chain lengths finely tuned by NPCA feed ratios (Figure 4(g) and Figures S45-S48; Table 1, entries 9-13). To further demonstrate the robustness of primary amine hydrochloride-initiated NPCA polymerizations, we also attempted one-pot synthesis of diblock and triblock copolypeptides under open-vessel conditions. Note that high-fidelity terminal amines of precursor sequences are crucial for successful synthesis of block copolypeptides. Starting from *n*-BuNH_3_^+^Cl^−^ initiator, sequential NPCA polymerizations were conducted in an ordinary fume hood, with the reaction vessel exposed to open air throughout the polymerization process (see inset in Figure 4(a)). Diblock and triblock copolypeptides including PTrp_30_-*b*-PNBO_30_, PTrp_30_-*b*-PNBO_45_, PTrp_30_-*b*-PNBO_30_-*b*-PCbzK_104_, and PTrp_30_-*b*-PNBO_45_-*b*-PCbzK_202_ with a predetermined sequence structure were successfully obtained (Figures S49-S53; Table 1). GPC elution traces after each chain extension revealed successive shift to higher molar masses, with *M*_*n*,NMR_ increasing from 5.7 kDa to 71.8 kDa and *Đ* in the range of 1.03-1.13 (Figure 4(h)). Finally, we demonstrate that polypeptide synthesis via NPCA polymerization initiated by primary amine hydrochloride is facile to scale up, as revealed by PNBO_10_ synthesis at gram scale under open-vessel condition (Figures S54 and S55). Furthermore, parallel synthesis of PCBZK_30_ at 50 mg and 1.5 g scale under either glovebox or open-vessel conditions all afforded well-defined polypeptides with comparable MW, low polydispersity (*Đ* ~1.1), and MALDI-TOF MS data exhibiting clean set of peaks (Figure 4(i) and Figures S56 and 57).

## 3. Conclusion

In conclusion, we developed a new strategy towards controlled polypeptide synthesis solely based on NAM via NPCA polymerization using primary amine hydrochloride as the initiator. Compared to conventional NCA polymerizations and amine-initiated NPCA polymerizations, primary amine hydrochloride-initiated NPCA polymerization possesses several distinct advantages. The polymerization is conducted under NCA monomer-starved condition; thus, AMM-relevant NCA oligomerization is suppressed; protonation/deprotonation equilibrium of peptidic terminal amines suppresses side reactions associated with amine basicity (i.e., NCA^−^ generation) and nucleophilicity (termination with solvents/isocyanate and cyclization). Moreover, released phenol during NPCA transformation into NCA could further eliminate NCA^−^ anions and inhibit the AMM pathway. All the above mentioned features lead to controlled synthesis of polypeptides with predetermined MWs, narrow polydispersity, and high-fidelity terminal functionalities. To illustrate the robustness, we further demonstrate controlled polypeptide synthesis under open-vessel condition, which is applicable for the synthesis of (block) copolypeptides.

## 4. Materials and Methods

### 4.1. Materials

2-Nitrobenzyoxycarbonyl-protected lysine (H-Lys(oNB)-OH) [61], *N*-*α*-carbobenzyloxy-2,4-diaminobutanoic acid (Cbz-Dab-OH) [62], *N*-*α*-carbobenzyloxy-*N*-*γ*-*tert*-butoxycarbonyl-2,4-diaminobutanoic acid (Cbz-Dab(Boc)-OH) [63], *o*-nitrobenzyl chloroformate [64], and (*S*)-1,3-benzothiazol-2-yl-*O*-phenylthiocarbonate [46] were synthesized according to previously reported literature procedures. All other anhydrous solvents were stored over 4 Å molecular sieve in nitrogen glovebox. All other chemicals were purchased from commercial sources and used as received.

### 4.2. Synthesis of Moisture-Stable NPCA Precursors

Synthetic routes employed for the preparation of NBDab, NBO, and NBK NPCA precursors are shown in Schemes S1 and S2. Synthetic routes employed for the preparation of BocDab, BocK, BocO, Phe, Trp, CbzO, and CbzK NPCA precursors are shown in Schemes S3 and S4. Detailed procedures of sample synthesis and relevant characterization data are described in Supplementary Materials (available here). The NPCA synthesis was typically conducted in THF/water mixture and completed within ~2 h with a yield up to ~85%.

Typical procedures for the synthesis of CbzK NPCA precursor are as follows. Into a mixture of H-Lys(Cbz)-OH (10.0 g, 35.67 mmol, 1.0 eq.), deionized water (80 mL), and sodium carbonate (3.78 g, 35.67 mmol, 1.0 eq.) thermostated at 40°C, the solution of (S)-1,3-benzothiazol-2-yl-O-phenylthiocarbonate (11.28 g, 39.24 mmol, 1.1 eq.) in THF (240 mL) was added dropwise. The mixture was vigorously stirred, and the reaction progress was monitored by TLC (EA, *R*_*f*_ = 0.6). After 2 h, the reaction mixture was diluted with 300 mL aqueous sodium bicarbonate (20 wt%). The organic solvent was then removed by rotary evaporation, and the precipitates were filtered off. Next, the aqueous layer was acidified to pH ~3 with 2.0 N HCl and extracted with EA (3 × 300 mL). The organic phase was combined and dried with anhydrous sodium sulfate. After removing all the solvent, the residues were further purified with column chromatography on silica gel using DCM/EA (2/1, *v*/*v*) as the eluent. The obtained crude product was recrystallized from *n*-hexane/EA, affording CbzK precursor as white powder (12.38 g, yield: 86.7%). ^1^H NMR (400 MHz, DMSO-*d*_6_, *δ*, ppm, Figure S8(a)): 12.72 (s, 1H, -COO**H**), 8.09 (s, 1H, -N**H**COOPh), 7.64-7.28 (m, 9H, Ar**H**), 7.21 (t, 1H, -N**H**COOCH_2_Ph), 7.13-7.02 (m, 2H, Ar**H**), 5.01 (s, 2H, -COOC**H**_2_-), 3.94 (m, 1H, -(COOH)C**H**-), 3.00 (m, 2H, -C**H**_2_NHCOO-), 1.27-1.79 (m, 6H, -C**H**_2_C**H**_2_C**H**_2_CH_2_NHCOO-). ^13^C NMR (101 MHz, MeOD, *δ*, ppm, Figure S8(b)): 174.10, 156.56, 154.92, 151.43, 137.73, 129.76, 128.82, 128.20, 125.46, 122.09, 65.59, 54.47, 40.59, 39.34, 30.84, 29.45, 23.38. ESI-MS (m/z): [M+Na]^+^ calcd. for C_21_H_24_N_2_O_6_Na, 423.1532; found: 423.1538 (Figure S8(c)).

### 4.3. Primary Amine Hydrochloride-Initiated NPCA Polymerization

Typical procedures employed for the polymerization of NBO precursor using *n*-BuNH_3_^+^Cl^−^ initiator in a nitrogen-purged glovebox are described below. NBO precursor was placed in a vial, and protonated amine initiator was added at varying [*M*]_0_/[*I*]_0_ molar ratios. Next, DMAc was added to maintain a constant [*M*]_0_ of 0.25 M. The reaction mixture was stirred at 70°C in glovebox for varying time durations. Taking the case of the [*M*]_0_/[*I*]_0_ ratio of 100 as an example, NBO precursor (100 mg, 0.23 mmol, 100 eq.) was placed in a vial and *n*-BuNH_3_^+^Cl^−^ initiator (6 mg/mL in DMAc) (42.4 *μ*L, 0.0023 mmol, 1.0 eq.) was added. DMAc (860 *μ*L) was then added, and the reaction mixture was stirred at 70°C in a glovebox. The NPCA conversion was assayed by ^1^H NMR in DMSO-*d*_6_. After the polymerization reached completion, the solution mixture was precipitated into an excess of cold diethyl ether and dried in a vacuum oven, affording the target PNBO polypeptide.

### 4.4. Kinetics Study of NPCA Polymerization Initiated by *n*-BuNH_3_^+^Cl^−^

In the nitrogen-purged glovebox, NBO precursor (100 mg, 0.23 mmol, 80.0 eq.) was placed in a glass vial and *n*-BuNH_3_^+^Cl^−^ (6 mg/mL in DMAc) (52.9 *μ*L, 0.0029 mmol, 1.0 eq.) was added. DMAc (850 *μ*L) was then added to reach an initial monomer concentration, [*M*]_0_, of 0.25 M. The reaction mixture was stirred at 70°C in the glovebox. During polymerization, 50 *μ*L aliquot of the reaction mixture was sampled out; ~20 *μ*L was immediately diluted with DMSO-*d*_6_ to determine monomer conversion via ^1^H NMR analysis; the remaining portion was diluted with the mobile phase of GPC (DMF), filtered through a 220 nm membrane, and directly subjected to GPC analysis to determine *M*_*n*_ and polydispersity index (*M*_*w*_/*M*_*n*_ or *Đ*) without further purification.

According to ^1^H NMR spectra shown in Figure 2(b) and Figure S11, the kinetics of NPCA polymerization including extents of NPCA consumption, NCA formation, and polypeptide formation could be calculated. Note that peaks *a*, *c*, and *d* in the range of 5.12-5.43 ppm are ascribed to methylene protons of oNB residues in NPCA, NCA, and polypeptide, and their total integration does not change during polymerization and could be used as an internal standard. The appearance of phenol signal (peaks *e*-*h*; peaks *e* and *f* at ~6.7 ppm was used for calculation) indicates the consumption of NPCA monomer and transformation into NCA monomer. Note that NCA will be further polymerized into polypeptide, and the instantaneous NCA concentration, [NCA]_*t*_, could be quantified from peak *b* at ~4.5 ppm. Relevant calculation protocols are as follows, and “*I*” refers to the integration area of the given NMR resonance peaks:

(1) $$\begin{array}{c} \text{Consumed NPCA}={\left[\text{NCA}\right]}_{t}+\text{formed polypeptide},\\ \text{Formed polypeptide}=\text{polymerized NPCA}={\left[\text{polypeptide}\right]}_{t},\\ \text{Consumed NPCA}=\frac{{\left[\text{NPCA}\right]}_{0}-{\left[\text{NPCA}\right]}_{t}}{{\left[\text{NPCA}\right]}_{0}}=\frac{I\left(e+f\right)/3}{I\left(a+c+d\right)/2}=\frac{2\;I\left(e+f\right)}{3\;I\left(a+c+d\right)},\\ \frac{{\left[\text{NCA}\right]}_{t}}{{\left[\text{NPCA}\right]}_{0}}=\frac{2\;I\left(b\right)}{I\left(a+c+d\right)},\\ \frac{{\left[\text{NPCA}\right]}_{t}}{{\left[\text{NPCA}\right]}_{0}}=1-\frac{2\;I\left(e+f\right)}{3\;I\left(a+c+d\right)},\\ {\left[\text{Polypeptide}\right]}_{t}=\text{consumed NPCA}-{\left[\text{NCA}\right]}_{t}=\frac{{\left[\text{NPCA}\right]}_{0}-{\left[\text{NPCA}\right]}_{t}}{{\left[\text{NPCA}\right]}_{0}}-\frac{{\left[\text{NCA}\right]}_{t}}{{\left[\text{NPCA}\right]}_{0}}=\frac{2\;I\left(e+f\right)-6\;I\left(b\right)}{3\;I\left(a+c+d\right)}. \end{array}$$

### 4.5. Polymerization of NPCA Precursors Initiated by *n*-BuNH_3_^+^Cl^−^ in Open Vessels Exposed to Air

The open-vessel polymerization was carried out in a general chemical laboratory with relatively high seasonal humidity (relative humidity > 80%; see the hygrometer in Figure 4(a) for details). The NPCA precursor was placed in a glass vial, and *n*-BuNH_3_^+^Cl^−^ (6 mg/mL in DMAc) was added at varying [*M*]_0_/[*I*]_0_ ratios. DMAc was then added to reach an initial monomer concentration, [*M*]_0_, of 0.25 M. The reaction mixture was directly exposed to air (i.e., no stopper, without inert gas protection) and stirred at 70°C in the fume hood. The extents of NPCA consumption and polypeptide conversion were measured by ^1^H NMR in DMSO-*d*_6_. After completion of polymerization, the reaction mixture was precipitated into an excess of diethyl ether and further drying in a vacuum oven afforded the target polypeptide product.

### 4.6. Synthesis of Diblock and Triblock Copolypeptides in Open Vessels via One-Pot Sequential Monomer Additions

Detailed procedures employed for sequential block copolymerization of NPCAs including Trp, NBO, and CbzK are as follows. NPCA precursors were, respectively, dissolved in DMAc to reach a concentration of 0.25 M and used as stock solution. The NPCA stock solution for the first block and *n*-BuNH_3_^+^Cl^−^ were charged into the reaction flask. The reaction mixture was directly exposed to air (i.e., no stopper, without protection of inert gas atmosphere) and stirred at 70°C in the fume hood. Upon completion of polymerization for the first block, an aliquot of the reaction mixture was sampled out for ^1^H NMR and GPC analysis. The NPCA stock solution for the second block was then added, and the chain extension process was conducted at 70°C under open-vessel condition. Upon completion of diblock and triblock copolymerization, the reaction mixture was precipitated into an excess of diethyl ether and further drying in a vacuum oven afforded the copolypeptide products. Structural parameters of the obtained block copolypeptides are summarized in Table 1.

Additional synthesis, characterization, and data are included in Supplementary Materials (available here).

## Figures and Tables

**Table 1:** Controlled synthesis of polypeptides via open-vessel polymerization of NPCA precursors in DMAc at 70°C using *n*-BuNH_3_^+^Cl^−^ initiator. Note that >99% polypeptide conversion was achieved for all entries.

Entry	Monomer	[*M*]_0_/[*I*]_0_	Time (h)	*M*_*n*,NMR_ (kDa)^a^	DP^a^	*M*_*n*,GPC_^b^ (*M*_*n*,MALDI_^c^) (kDa)	*Đ*_GPC_^d^ (*Đ*_MALDI_^e^)
1	NBO	10	24	2.9	10	2.9 (3.0^c^)	1.07 (1.06^e^)
2	CbzK	30	48	7.9	30	8.4 (7.6^c^)	1.12 (1.10^e^)
3	CbzO	9	24	2.3	9	2.2 (2.1^c^)	1.06 (1.05^e^)
4	Trp	12	24	2.1	10	0.95 (2.2^c^)	1.04 (1.05^e^)
5	NBK	8	24	2.6	8	2.4 (2.5^c^)	1.05 (1.04^e^)
6	NBK	25	36	7.8	25	7.7	1.06
7	NBK	45	36	13.6	44	12.8	1.08
8	NBK	60	48	18.5	60	16.3	1.10
9	NBK	70	60	22.2	72	19.8	1.09
10	NBK	80	72	24.9	81	23.1	1.13
11	NBK	90	72	28.6	93	27.5	1.15
12	NBK	120	72	36.0	117	34.8	1.17
13	NBO-*r*-Phe-*r*-BocK^f^	11/4.4/4.6	24	5.5	22	5.1	1.03
14	NBO-*r*-Phe-*r*-BocK^f^	12/6/12	36	7.5	31	7.3	1.03
15	NBO-*r*-Phe*-r*-BocK^f^	25/10/15	48	12.4	51	10.5	1.07
16	NBO-*r*-Phe*-r*-BocK^f^	35/21/14	60	16.3	70	14.7	1.09
17	NBK-*r*-Phe-*r*-BocK-*r*-Trp^f^	20/20/25/35	72	21.1	99	21.6	1.14
18	Trp	30	36 (block 1)	5.7	30	5.1	1.03
19	Trp-*b*-NBO^g^	30/30	24 (block 2)	14.4	30/30	14.1	1.06
20	Trp-*b*-NBO^g^	30/45	24 (block 2)	18.8	30/45	17.9	1.05
21	Trp-*b*-NBO-*b*-CbzK^g^	30/30/100	48 (block 3)	46.1	30/30/104	31.9	1.13
22	Trp-*b*-NBO-*b*-CbzK^g^	30/45/200	72 (block 3)	71.8	30/45/202	47.5	1.06

^a^Calculated from ^1^H NMR spectra. ^b^Determined by GPC using refractive index (RI) detector (eluent: DMF, 10 mM LiBr; 1 mL/min). ^c^Number-average molecular weight, *M*_*n*,MALDI_, determined by MALDI-TOF MS. ^d^Polydispersity index (*M*_*w*_/*M*_*n*_) determined by GPC unless otherwise noted. ^e^Polydispersity index (*M*_*w*_/*M*_*n*_) determined by MALDI-TOF MS. ^f^Random copolypeptides synthesized by one-pot NPCA copolymerization in open vessel exposed to air. ^g^Diblock and triblock copolypeptides synthesized by sequential NPCA polymerizations in open vessel exposed to air.

## Data Availability

All data needed in the paper are present in the paper and in the supplementary section. Additional data related to this paper may be requested from the authors.

## Supplementary Materials

Supplementary Materials Scheme S1: synthetic routes employed for the preparation of NBDab precursor. Scheme S2: synthetic routes employed for the preparation of NBO and NBK precursors. Scheme S3: synthetic routes employed for the preparation of BocDab precursor. Scheme S4: synthetic routes employed for the preparation of BocK, BocO, Phe, Trp, CbzO, and CbzK precursors. Figure S1: comparison of the mechanisms of NPCA polymerization using protonated primary amine versus conventional primary amine as initiators. Figure S2: characterization of NBDab precursor. Figure S3: characterization of NBO precursor. Figure S4: macroscopic images of moisture-insensitive and air-stable NBO precursor. Figure S5: characterization of NBK precursor. Figure S6: characterization of BocDab precursor. Figure S7: characterization of NBO precursor. Figure S8: characterization of CbzK precursor. Figure S9: (a) GPC elution traces recorded for PNBO synthesized using *n*-BuNH_2_ as initiator. (b) Mn, GPC, and *Đ* of as-synthesized PNBO. Figure S10: GPC elution traces recorded for polypeptides synthesized using *n*-BuNH_2_ as the initiator in the presence of acetic acid. Figure S11: schematics illustrating the calculation of polymerization kinetics. Figure S12: (a) ^1^H NMR, (b) ^13^C NMR, and (c) ^19^F NMR spectra recorded for *n*-BuNH_3_^+^BF_4_^−^. Figure S13. (a) ^1^H NMR, (b) ^13^C NMR, and (c) ^19^F NMR spectra recorded for *n*-BuNH_3_^+^PF_6_^−^. Figure S14: (a) ^1^H NMR and (b) ^13^C NMR spectra recorded for *n*-BuNH_3_^+^ClO_4_^−^. Figure S15: (a) ^1^H NMR and (b) ^13^C NMR spectra recorded for *n*-BuNH_3_^+^Br^−^. Figure S16: evolution of GPC elution traces recorded for NPCA polymerizations under various conditions. Figure S17: comparison of CbzK polymerizations at 70°C (a) with and (b) without HCl addition in the absence of amine or ammonium initiator. Figure S18: comparison of NPCA polymerizations using (a) *n*-BuNH_3_^+^Cl^−^, (b) *n*-BuNH_3_^+^BF_4_^−^, and (c) *n*-BuNH_2_ as initiator ([*M*]_0_/[*I*]_0_ = 30). Figure S19: GPC elution traces recorded for NPCA polymerization at feed ratios of (a) 10, (b) 20, and (c) 30 using *n*-BuNH_3_^+^BF_4_^−^ initiator at 70°C. Figure S20: MALDI-TOF MS spectra recorded for PNBO synthesized at [*M*]_0_/[*I*]_0_ ratios of 5, 7, and 10 using *n*-BuNH_3_^+^Cl^−^ as the initiator. Figure S21: evolution of MALDI-TOF MS spectra with polypeptide conversions recorded for the synthesis of PCbzK in a glovebox. Figure S22: MALDI-TOF MS spectrum recorded for PCbzK_15_ synthesized using *n*-BuNH_3_^+^Cl^−^ as initiator. Figure S23: MALDI-TOF MS spectrum recorded for PCbzK_20_ synthesized using *n*-BuNH_3_+Cl^−^ as initiator. Figure S24: (a) GPC elution trace and (b) MALDI-TOF MS spectrum recorded for PNBO_5_ synthesized using *n*-BuNH_3_^+^Cl^−^ as initiator. Figure S25: (a) GPC elution trace and (b) MALDI-TOF MS spectrum recorded for PNBDab_12_ synthesized using *n*-BuNH_3_^+^Cl^−^ as initiator. Figure S26: (a) GPC elution trace and (b) MALDI-TOF MS spectrum recorded for PNBK_9_ synthesized using *n*-BuNH_3_^+^Cl^−^ as initiator. Figure S27: (a) GPC elution traces of PTrp polypeptides. (b) GPC elution traces of PCbzO polypeptides. (c) ATR-FT-IR spectra recorded for PCbzO_7_, PCbzO_65_, and PCbzO_102_. (d) Circular dichroism (CD) spectra recorded for PCbzO_7_, PCbzO_65_, PCbzO_83_, and PCbzO_102_ in HFIP at 20°C (0.05 mg/mL). Figure S28: GPC elution traces and MALDI-TOF MS spectra recorded for PCbzK synthesized using varying initiators. Figure S29: comparisons of GPC elution traces and CD spectra for CbzK polymerization products at a feed ratio of 7 using *n*-BuNH_3_^+^Cl^−^ and *n*-BuNH_2_ as initiator. Figure S30: MALDI-TOF MS recorded for CbzK polymerizations at a feed ratio of 7 using (a) *n*-BuNH_2_, (b) *n*-BuNH_3_^+^BF_4_^−^, and (c) *n*-BuNH_3_^+^Cl^−^ as initiator. Figure S31: MALDI-TOF MS data recorded for PCbzK polypeptides synthesized at [CbzK]/[*I*] ratios of (a) 7 and (b) 20 using *n*-BuNH_2_, *n*-BuNH_3_^+^BF_4_^−^, and *n*-BuNH_3_^+^Cl^−^ as initiators. Figure S32: MALDI-TOF MS spectra recorded for (a) PTrp, (b) PCbzO, (c) PBocO, and (d) PBocDab polypeptides synthesized using *n*-BuNH_2_ as the initiator. Figure S33: MALDI-TOF MS spectra recorded for (a) PBocO, (b) PTrp, and (c) PNBO synthesized using primary amine initiator in the presence of acetic acid. Figure S34: MALDI-TOF MS recorded for PCbzK synthesized using *n*-BuNH_3_^+^BF_4_^−^ as initiator at a feed ratio of 10. Figure S35: FT-IR spectra recorded for *n*-BuNH_3_^+^Cl^−^ initiator in DMAc solvent at varying temperatures. Figure S36: ^1^H NMR spectra recorded for commercial anhydrous DMAc, AR-grade DMAc, and time-dependent evolution of water contents of DMAc solvent during open-vessel polymerization. Figure S37: (a) GPC elution trace, (b) ^1^H NMR spectrum, and (c) MALDI-TOF MS spectrum recorded for PNBO_10_ synthesized under open-vessel condition. Figure S38: evolution of MALDI-TOF MS spectra recorded at varying conversions for open-vessel polymerization of CbzK precursor using *n*-BuNH_3_^+^Cl^−^ as initiator. Figure S39: MALDI-TOF MS spectra recorded for PCbzK synthesized under open-vessel condition using *n*-BuNH_3_^+^Cl^−^ as initiator at varying [*M*]_0_/[*I*]_0_ ratios. Figure S40: MALDI-TOF MS spectra recorded for PCbzK synthesized under open-vessel condition using *n*-BuNH_3_^+^Cl^−^ as initiator at varying [*M*]_0_/[*I*]_0_ ratios. Figure S41: ^1^H NMR spectrum recorded for PTrp_11_ synthesized under open-vessel condition. Figure S42: ^1^H NMR spectrum recorded for PCbzO_9_ synthesized under open-vessel condition. Figure S43: ^1^H NMR spectrum recorded for PNBK_8_ synthesized under open-vessel condition. Figure S44: MALDI-TOF MS spectrum recorded for CbzK polymerization under open-vessel condition using *n*-BuNH_2_ initiator. Figure S45: ^1^H NMR spectrum recorded for Poly(BocK_0.23_-r-NBO_0.54_-r-Phe_0.23_)_22_ synthesized under open-vessel condition. Figure S46: ^1^H NMR spectrum recorded for Poly(BocK_0.39_-r-NBO_0.42_-r-Phe_0.19_)_31_ synthesized under open-vessel condition. Figure S47: ^1^H NMR spectrum recorded for Poly(BocK_0.31_-r-NBO_0.47_-r-Phe_0.22_)_51_ synthesized under open-vessel condition. Figure S48: ^1^H NMR spectrum recorded for Poly(BocK_0.20_-r-NBO_0.47_-r-Phe_0.33_)_70_ synthesized under open-vessel condition. Figure S49: ^1^H NMR spectrum recorded for PTrp_30_ synthesized in the open vessel exposed to air. Figure S50: ^1^H NMR spectrum recorded for PTrp_30_-b-PNBO_30_ diblock copolypeptide synthesized in the open vessel exposed to air. Figure S51: ^1^H NMR spectrum recorded for PTrp_30_-b-PNBO_45_ diblock copolypeptide synthesized in the open vessel exposed to air. Figure S52: ^1^H NMR spectrum recorded for PTrp_30_-b-PNBO_30_-b-PCbzK_104_ triblock copolypeptide synthesized in the open vessel exposed to air. Figure S53: ^1^H NMR spectrum recorded for PTrp_30_-b-PNBO_45_-b-PCbzK_202_ triblock copolypeptide synthesized in the open vessel exposed to air. Figure S54: (a–c) Schematics illustrating gram scale synthesis of PNBO polypeptide initiated by *n*-BuNH_3_^+^Cl^−^ under open-vessel condition. (d) GPC elution traces recorded for PNBO_10_ and PNBO_100_. (e) MALDI-TOF MS spectrum recorded for PNBO_10_ synthesized via open-vessel polymerization of NBO precursor at gram scale. Figure S55: ^1^H NMR spectrum recorded for PNBO_10_ synthesized via open-vessel polymerization of NBO precursor at gram scale. Figure S56: MALDI-TOF MS spectra and GPC elution traces recorded for PCbzK polypeptide synthesized under different conditions: (a, d) inside glovebox, tens of milligram scale; (b, e) open-vessel, tens of milligram scale; (c, f) open-vessel, gram scale. Figure S57: ^1^H NMR spectrum recorded for PCbzK_30_ synthesized via open-vessel polymerization of CbzK precursor at gram scale. Figures S58-S78: GPC raw data for polypeptides discussed in the main text and Supplementary Materials. Table S1: summary of polypeptides synthesized via polymerization of NPCA precursors initiated by *n*-BuNH_3_^+^Cl^−^. Table S2: summary of polypeptides synthesized via polymerization of NBO precursor under varying conditions.
