# Supplementary material for: Synthesis of Polypeptides with High-Fidelity Terminal Functionalities under NCA Monomer-Starved Conditions
Source: Research (Wash D C). 2021 Nov 17;2021:9826046. doi: 10.34133/2021/9826046 (PMC8617576; doi:10.34133/2021/9826046)
Supplement: Supplementary Materials — Scheme S1: synthetic routes employed for the preparation of NBDab precursor. Scheme S2: synthetic routes employed for the preparation of NBO and NBK precursors. Scheme S3: synthetic routes employed for the preparation of BocDab precursor. Scheme S4: synthetic routes employed for the preparation of BocK, BocO, Phe, Trp, CbzO, and CbzK precursors. Figure S1: comparison of the mechanisms of NPCA polymerization using protonated primary amine versus conventional primary amine as initiators. Figure S2: characterization of NBDab precursor. Figure S3: characterization of NBO precursor. Figure S4: macroscopic images of moisture-insensitive and air-stable NBO precursor. Figure S5: characterization of NBK precursor. Figure S6: characterization of BocDab precursor. Figure S7: characterization of NBO precursor. Figure S8: characterization of CbzK precursor. Figure S9: (a) GPC elution traces recorded for PNBO synthesized using n-BuNH2 as initiator. (b) Mn, GPC, and Đ of as-synthesized PNBO. Figure S10: GPC elution traces recorded for polypeptides synthesized using n-BuNH2 as the initiator in the presence of acetic acid. Figure S11: schematics illustrating the calculation of polymerization kinetics. Figure S12: (a) 1H NMR, (b) 13C NMR, and (c) 19F NMR spectra recorded for n-BuNH3+BF4−. Figure S13. (a) 1H NMR, (b) 13C NMR, and (c) 19F NMR spectra recorded for n-BuNH3+PF6−. Figure S14: (a) 1H NMR and (b) 13C NMR spectra recorded for n-BuNH3+ClO4−. Figure S15: (a) 1H NMR and (b) 13C NMR spectra recorded for n-BuNH3+Br−. Figure S16: evolution of GPC elution traces recorded for NPCA polymerizations under various conditions. Figure S17: comparison of CbzK polymerizations at 70°C (a) with and (b) without HCl addition in the absence of amine or ammonium initiator. Figure S18: comparison of NPCA polymerizations using (a) n-BuNH3+Cl−, (b) n-BuNH3+BF4−, and (c) n-BuNH2 as initiator ([M]0/[I]0 = 30). Figure S19: GPC elution traces recorded for NPCA polymerization at feed ratios of (a) 10, (b) [file 9826046.f1.pdf]

Supplementary Information

Synthesis of Polypeptides with High-Fidelity Terminal Functionalities under NCA Monomer-Starved Conditions

Lei Li,[1] Jie Cen,[1] Wenhao Pan,[1] Yuben Zhang,[1] Xuanxi Leng,[1] Zhengqi Tan,[1] Hao Yin,[2] and Shiyong Liu[1]*

[1]Hefei National Laboratory for Physical Sciences at the Microscale, Department of Polymer Science and Engineering, School of Chemistry and Materials Science, University of Science and Technology of China, Hefei, Anhui 230026, China.

[2]Mass Spectrometry Lab, Hefei National Laboratory for Physical Sciences at the Microscale, University of Science and Technology of China, Hefei, Anhui 230026, China

*Correspondence should be addressed to Shiyong Liu; sliu@ustc.edu.cn

Experimental Section

Materials. Boc-Gln-OH, Cbz-Gln-OH, *L*-phenylalanine, H-Orn(Cbz)-OH, H-Lys(Cbz)-OH, tetrabutylammonium hydroxide (40 wt% in methanol), hydrogen chloride solution in methanol (4.0 M), 4-dimethylaminopyridine (DMAP), and di-*tert*-butyldicarbonate (Boc anhydride) were purchased from Energy Chemical Co., Ltd. and used as received. L-Lysine hydrochloride and hydrobromic acid were purchased from Aladdin Reagent Co., Ltd. and used as received. H-Lys(Boc)-OH and (diacetoxyiodo)benzene (PIDA) were purchased from Shanghai 9Ding Chemical Co., Ltd. and used as received. *L*-Ornithine hydrochloride (Aldrich), H-Orn(Boc)-OH (GL Biochem Co., Ltd.), and tetrafluoroboric acid diethyl ether complex (50-55 wt% HBF_4; J&K Scientific Co., Ltd) were used as received. Anhydrous *N*,*N*-dimethylacetamide (DMAc, extra dry over molecular sieve) was purchased from Thermo Fisher Scientific Inc. and stored in nitrogen glovebox prior to use. *n*-Butylamine was purchased from Sigma-Aldrich and purified by distillation over calcium hydride (CaH_2) prior to use. *n*-Butylamine hydrochloride was purchased from Tokyo Chemical Industry Co., Ltd. and recrystallized from diethyl ether/MeOH prior to use. Lithium bromide (LiBr) was purchased from Sigma-Aldrich. L-Tryptophan, sodium acetate (NaOAc), sodium hydroxide (KOH), potassium carbonate (K_2CO_3), sodium bicarbonate ($NaHCO_3$), sodium sulfate ($NaSO_4$), copper sulfate ($CuSO_4$), 1,4-dioxane, petroleum ether (PE), ethyl acetate (EA), acetonitrile (MeCN), tetrahydrofuran (THF), methanol, *n*-hexane, acetone, ethanol, diethyl ether, *N*,*N*-dimethylformamide (DMF), dimethyl sulfoxide (DMSO), acetic acid, toluene, $CuSO_4{\cdot}5H_2O$, ethylenediaminetetraacetic acid disodium salt (EDTA), pyridine, hexafluorophosphoric acid, perchloric acid, triethylamine (TEA), and all other reagents/solvents were purchased from Sinopharm Chemical Reagent Co., Ltd. and used as received unless otherwise noted. Water was deionized with a Milli-Q SP reagent water system (Millipore) to a specific resistivity of 18.4 MΩ cm. All other anhydrous solvents were stored over 4 Å molecular sieve in nitrogen glovebox.

Methods. Synthetic routes employed for the preparation of NPCA precursors are shown in Schemes S1-S4.

Synthesis of Boc-Dab-OH (Scheme S1). Typical procedures employed for the synthesis of 2-*N*-*tert*-butoxycarbonyl-(*S*)-4-diaminobutanoic acid (Boc-Dab-OH) are described below. Into a solution mixture of Boc-Gln-OH (15.0 g, 60.9 mmol, 1.0 eq.) in THF (320 mL) and water (80 mL) pre-cooled to ~4 °C, PIDA (23.54 g, 73.09 mmol, 1.2 eq.) was added. After stirring for 8 h, the reaction mixture was evaporated to dryness, the residues were dissolved in water (250

mL) and extracted with EA (3 × 60 mL). The organic layer was discarded and the aqueous layer was evaporated to dryness. The residues were washed with cold chloroform for three times and dried under vacuum, the obtained pale yellow solid, Boc-Dab-OH, was directly used for the next step (9.91 g, yield: 74.6%).

Synthesis of Boc-Dab(oNB)-OH (Scheme S1). To a stirred solution of Boc-Dab-OH (8.0 g, 36.7 mmol, 1.0 eq.) and H_2O (100 mL) in an ice-water bath, aqueous NaOH solution (1.0 M) was added dropwise to adjust the solution pH to ~10. Newly synthesized *o*-nitrobenzyl chloroformate (9.48 g, 43.99 mmol, 1.2 eq.) was added and the reaction mixture was stirred overnight in the dark at room temperature. After the reaction was completed, the solution was adjusted to pH ~2-3 with 2.0 N HCl and extracted with EA (3 × 100 mL). The organic phase was evaporated to dryness under reduced pressure and the residues were further purified with column chromatography on silica gel using PE/EA (2/1, v/v) as the eluent. After drying under vacuum, the target product was obtained as a thick solid (9.17 g, yield: 63.0%), which was directly used for the next step.

Synthesis of H-Dab(oNB)-OH (Scheme S1). Into the solution of Boc-Dab(oNB)-OH (8.0 g, 0.02 mol, 1.0 eq.) in dichloromethane (DCM, 100 mL), TFA was slowly added and the reaction mixture was allowed to stir in the dark under ambient temperature. The progress of the reaction was monitored by TLC until the starting material was completely consumed. The solution pH was adjusted to ~9 with saturated aqueous sodium carbonate, and then extracted with DCM (3 × 50 mL); the aqueous phase was retained and the solution pH was adjusted to ~7. After cooling in an ice-water bath, the formed precipitates were collected by filtration. After drying under vacuum, H-Dab(oNB)-OH was obtained as white solid powder (4.85 g, yield: 81.1%). ^{1}H NMR (400 MHz, D_2O, δ, ppm, Figure S2(a)): 7.82 (s, 1H, Ar**H**), 7.46 (s, 1, Ar**H**), 7.28 (m, 2H, Ar**H**), 5.16 (s, 2H, -COOC**H**$_2$-), 3.84 (t, 1H, -(COOH)C**H**-), 3.08 (s, 2H, -C**H**$_2$NHCOO-), 1.89 (s, 2H, C**H**$_2$CH$_2$NHCOO-). ^{13}C NMR (101 MHz, D_2O, δ, ppm, Figure S2(b)): 171.42, 158.06, 134.38, 132.04, 128.98, 128.64, 124.99, 63.81, 50.26, 36.24, 29.72. ESI-MS (m/z): $[M+Na]^+$ calcd. for $C_{12}H_{15}N_3O_6Na$, 320.0853; found: 320.08499 (Figure S2(c)).

Synthesis of NBDab Precursor (Scheme S1). Into a mixture of H-Dab(oNB)-OH (2.0 g, 6.73 mmol, 1.0 eq.), deionized water (8 mL), and sodium carbonate (0.69 g, 6.73 mmol, 1.0 eq.), (*S*)-1,3-benzothiazol-2-yl-*O*-phenylthiocarbonate (2.13 g, 7.40 mmol, 1.1 eq.) solution in THF (24 mL) was added dropwise at 40 °C. The reaction mixture was vigorously stirred and the progress was monitored by TLC (EA eluent, R_f ~0.5). After ~2 h, the mixture was diluted with 30 mL of aqueous sodium bicarbonate (20 wt%). After evaporating off the organic solvent, the mixture was filtered. The remaining aqueous phase was acidified to pH ~3 with 2.0 N HCl

and extracted with EA (3 × 200 mL). The organic layer was dried using anhydrous sodium sulfate and filtered. After evaporating the solvent, the crude product was further purified by column chromatography on silica gel using DCM/EA (2/1, v/v) as the eluent. The product was further purified via recrystallization from *n*-hexane/EA mixture, affording the NBDab precursor as a white powder (2.32 g, yield: 82.5%). ^{1}H NMR (400 MHz, DMSO-d_6, δ, ppm, Figure S2(d)): 12.85 (s, 1H, -COO**H**), 8.22 (m, 2H, Ar**H**), 7.84 (t, 1H, -N**H**COOPh), 7.71 (m, 3H, Ar**H**), 7.44 (t, 2H, Ar**H**), 7.29 (t, 1H, -N**H**COO-), 7.20 (d, 2H, ArH), 5.46 (s, 2H, -COOC**H**$_2$-), 4.13 (m, 1H, -(COOH)C**H**-), 3.24 (m, 2H, -C**H**$_2$NHCOO-), 1.89-2.06 (m, 2H, -C**H**$_2$CH$_2$NHCOO-). ^{13}C NMR (101 MHz, DMSO-d_6, δ, ppm, Figure S2(e)): 173.94, 156.14, 154.88, 151.45, 147.57, 134.60, 133.49, 129.74, 129.36, 129.16, 125.47, 125.23, 122.08, 62.47, 52.04, 39.55, 37.79, 31.09. ESI-MS (m/z): $[M+H]^+$ calcd. for $C_{19}H_{20}N_3O_8$, 418.12504; found: 418.12466 (Figure S2(f)).

Synthesis of H-Orn(oNB)-OH (Scheme S2). Typical procedures employed for the synthesis of 2-nitrobenzyoxycarbonyl-protected ornithine, H-Orn(oNB)-OH, are described below. Into a round-bottom flask, *L*-ornithine hydrochloride (15.0 g, 88.97 mmol, 1.0 eq.) and deionized water (500 mL) were added. Into this mixture, sodium hydroxide (7.11 g, 177.9 mmol, 2.0 eq.) was slowly added at 0 ^{o}C. Next, $CuSO_4{\cdot}5H_2O$ (11.12 g, 44.5 mmol, 0.5 eq.) was added and the reaction mixture was allowed to stir at room temperature for 6 h. The reaction mixture was then cooled to 0 ^{o}C in an ice-water bath, and *o*-nitrobenzyl chloroformate (23.01 g, 106.8 mmol, 1.2 eq.) in THF (80 mL) was added, along with sodium bicarbonate (8.96 g, 106.8 mmol, 1.2 eq.). The reaction mixture was allowed to stir overnight at room temperature. The formed blue solid residues were collected by filtration, washed with water, dried, and used immediately for the subsequent step. Into a vigorously stirred suspension of the above intermediate product dispersed in 700 mL water at 80 ^{o}C, EDTA (25.72 g, 76.51 mmol, 0.86 eq.) was added. The reaction mixture was allowed to stir several minutes until most of the solids dissolved into the solution. The blue suspension maintained at 80 ^{o}C was filtered and insoluble residues were washed with hot water. Upon cooling down to room temperature, the crude product started to recrystallize and precipitate out of the solution. The solid product was collected by suction filtration, and washed with water (3 × 80 mL), THF (3 × 80 mL), and diethyl ether (3 × 80 mL). For further purification, the obtained white solids were dissolved in acidic aqueous media (pH 2.0, 200 mL), and insoluble residues were removed, and the filtrate was adjusted to pH ~7.4; the newly formed white precipitates were filtered, affording H-Orn(oNB)-OH as white powder (23.6 g, yield: 85.2%). ^{1}H NMR (400 MHz, D_2O, δ, ppm, Figure S3(a)): 7.91 (d, 1H, Ar**H**), 7.55 (t, 1H, Ar**H**), 7.43 (m, 2H, Ar**H**), 5.24 (s, 2H, -COOC**H**$_2$-), 3.91 (t, 1H, -(COOH)C**H**-),

3.00 (t, 2H, -C**H**$_2$NHCOO-), 1.76 (m, 2H, -C**H**$_2$CH$_2$CH$_2$NHCOO-), 1.44 (m, 2H, -CH$_2$C**H**$_2$CH$_2$NHCOO-). ^{13}C NMR (101 MHz, D$_2$O, δ, ppm, Figure S3(b)): 171.63, 157.56, 146.52, 134.24, 129.16, 128.27, 125.26, 63.36, 52.33, 39.16, 26.47, 24.58. ESI-MS (m/z): $[M+H]^+$ calcd. for $C_{13}H_{18}N_3O_6$, 312.11956; found: 312.05518 (Figure S3(c)).

Synthesis of NBO Precursor (Scheme S2). Into a mixture of H-Orn(oNB)-OH (10.0 g, 32.12 mmol, 1.0 eq.) in 80 mL of water and sodium carbonate (3.4 g, 32.12 mmol, 1 eq.) thermostated at 40 °C, the solution of (*S*)-1,3-benzothiazol-2-yl-*O*-phenylthiocarbonate (10.15 g, 35.33 mmol, 1.10 eq.) in THF (240 mL) was added dropwise. The mixture was vigorously stirred and the reaction progress was monitored by TLC (EA, R_f = 0.65). After 2 h, the reaction mixture was diluted with 300 mL aqueous sodium bicarbonate (20 wt%). The organic solvent was then removed by rotary evaporation, and the precipitates were filtered off. Next, the aqueous layer was acidified to pH ~3 with 2.0 N HCl and extracted with EA (3 × 300 mL). The organic layer was combined and dried with anhydrous sodium sulfate. After removing all the solvent, the residues were further purified with column chromatography on silica gel using DCM/EA (2/1, v/v) as the eluent. The obtained crude product was recrystallized from *n*-hexane/EA, affording NBO precursor as white powder (11.67 g, yield: 84.2%; see Figure S4 for macroscopic images). ^{1}H NMR (400 MHz, DMSO-d_6, δ, ppm, Figure S3(d)): 8.06 (t, 2H, Ar**H**), 7.78 (t, 1H, Ar**H**), 7.60 (m, 2H, Ar**H**), 7.49 (t, 1H, -N**H**COOPh), 7.38 (t, 2H, Ar**H**), 7.21 (t, 1H, -N**H**COO-), 7.08 (d, 2H, ArH), 5.37 (s, 2H, -COOC**H**$_2$-), 3.96 (m, 1H, -(COOH)C**H**-), 3.04 (m, 2H, -C**H**$_2$NHCOO-), 1.50-1.86 (m, 4H, -C**H**$_2$C**H**$_2$CH$_2$NHCOO-). ^{13}C NMR (101 MHz, CDCl$_3$, δ, ppm, Figure S3(e)): 175.09, 156.35, 154.67, 150.75, 147.37, 133.81, 132.95, 129.33, 128.76, 128.49, 125.57, 124.96, 121.41, 77.20, 63.60, 53.53, 40.47, 29.21, 25.61. ESI-MS (m/z): $[M+Na]^+$ calcd. for $C_{20}H_{21}N_3O_8Na$, 454.12263; found: 454.16290 (Figure S3(f)).

Synthesis of H-Lys(oNB)-OH (Scheme S2). Typical procedures employed for the synthesis of 2-nitrobenzyoxycarbonyl-protected lysine (H-Lys(oNB)-OH) are described below. Into a round-bottom flask, L-lysine hydrochloride (20 g, 109.5 mmol, 1 eq.) and deionized water (700 mL) were added. Into this mixture, sodium hydroxide (8.76 g, 219.0 mmol, 2 eq.) was slowly added at 0 °C. Next, $CuSO_4 \cdot 5H_2O$ (13.69 g, 54.75 mmol, 0.5 eq.) was added and the reaction mixture was allowed to stir at room temperature for 6 h. The reaction mixture was then cooled to 0 °C in an ice-water bath, and *o*-nitrobenzyl chloroformate (28.33 g, 131.4 mmol, 1.2 eq.) in THF (100 mL) was added, along with sodium bicarbonate (11.04 g, 131.4 mmol, 1.2 eq.). The reaction mixture was allowed to stir overnight at room temperature. The formed blue solid residues were collected by filtration, washed with water, dried, and used immediately for the

next step. Into a vigorously stirred suspension of the above intermediate product dispersed in 600 mL water at 80 °C, EDTA (35.05 g, 94.17 mmol, 0.86 eq.) was added. The reaction mixture was allowed to stir for 5 min until most of the solids dissolved into the solution. The blue suspension maintained at 80 °C was filtered and insoluble residues were thoroughly washed with hot water. Upon cooling down to room temperature, the crude product started to recrystallize and precipitate out of the solution. The solid product was collected by suction filtration and successively washed with water (3 × 80 mL), THF (3 × 80 mL), and diethyl ether (3 × 80 mL). For further purification, the obtained white solids were dissolved in acidic aqueous media (pH ~2, 200 mL) and insoluble residues were removed, the obtained filtrate was adjusted to pH ~7.4; the newly formed white precipitates were collected via suction filtration, affording the target product, H-Lys(oNB)-OH, as white powder (28.7 g, yield: 80.6%). ^{1}H NMR (400 MHz, D_2O, δ, ppm, Figure S5(a))：7.92 (d, 1H, Ar**H**), 7.54 (t, 1H, Ar**H**), 7.39 (m, 2H, Ar**H**), 5.19 (s, 2H, -COOC**H**$_2$-), 3.92 (t, 1H, -C**H**-), 2.96 (t, 2H, -C**H**$_2$NHCOO-), 1.79 (m, 2H, -C**H**$_2$CH$_2$CH$_2$CH$_2$NH-), 1.36 (m, 4H, -CH$_2$C**H**$_2$C**H**$_2$CH$_2$NH-). ^{13}C NMR (101 MHz, D_2O, δ, ppm, Figure S5(b)): 171.89, 157.38, 146.18, 134.42, 131.79,127.93, 124.93, 63.42, 52.68, 39.55, 29.20, 27.84, 21.29. ESI-MS (m/z): $[M+H]^+$ calcd. for $C_{14}H_{20}N_3O_6$, 326.13521; found: 326.08991 (Figure S5(c)).

Synthesis of NBK Precursor (Scheme S2). Into a mixture of H-Lys(oNB)-OH (5.0 g, 15.37 mmol, 1.0 eq.), deionized water (40 mL), and sodium carbonate (1.62 g, 15.37 mmol, 1.0 eq.) thermostated at 40 °C, the solution of (*S*)-1,3-benzothiazol-2-yl-*O*-phenylthiocarbonate (4.86 g, 16.91 mmol, 1.1 eq.) in THF (120 mL) was added dropwise. The mixture was vigorously stirred and the reaction progress was monitored by TLC (EA, R_f = 0.66). After 2 h, the reaction mixture was diluted with 150 mL aqueous sodium bicarbonate (20 wt%). The organic solvent was then removed by rotary evaporation and the precipitates were filtered off. Next, the aqueous layer was acidified to pH ~3 with 2.0 N HCl and extracted with EA (3 × 200 mL). The organic phase was combined and dried with anhydrous sodium sulfate. After removing all the solvent, the residues were further purified with column chromatography on silica gel using DCM/EA (2/1, v/v) as the eluent. The obtained crude product was recrystallized from *n*-hexane/EA, affording NBK precursor as white powder (5.84 g, yield: 85.4%). ^{1}H NMR (400 MHz, DMSO-d_6, δ, ppm, Figure S5(d)): 8.10 (m, 2H, Ar**H**), 7.79 (t, 1H, Ar**H**), 7.63 (m, 2H, Ar**H**), 7.47 (t, 1H, -N**H**COOPh), 7.38 (t, 2H, Ar**H**), 7.20 (d, 1H, -N**H**COO-), 7.10 (d, 2H, Ar**H**), 5.36 (s, 2H, -COOC**H**$_2$-), 3.95 (m, 1H, -(COOH)C**H**-), 3.01 (m, 2H, -C**H**$_2$NHCOO-), 1.83-1.31 (m, 6H, -C**H**$_2$C**H**$_2$C**H**$_2$CH$_2$NHCOO-). ^{13}C NMR (101 MHz, MeOD, δ, ppm, Figure S5(e)): 174.28,

156.99, 156.94, 155.74, 155.68, 151.20, 147.42, 133.54, 133.16, 128.92, 128.37, 128.29, 124.99, 124.45, 121.38, 62.67, 54.04, 47.62, 40.13, 30.92, 28.98, 22.44. ESI-MS (m/z): $[M+Na]^+$ calcd. for $C_{21}H_{23}N_3O_8Na$, 468.13828; found: 468.13309 (Figure S5(f)).

Synthesis of Cbz-Dab-OH (Scheme S3). Typical procedures employed for the synthesis of Cbz-Dab-OH are described below. Into a mixture of Cbz-Gln-OH (20.0 g, 58.6 mmol, 1.0 eq.) in THF (400 mL) and water (100 mL) pre-cooled to ~4 ^{o}C, PIDA (22.7 g, 70.32 mmol, 1.2 eq.) was added. After stirring for 8 h, the reaction mixture was evaporated to dryness, the residues were dissolved in water (300 mL) and extracted with EA (3 × 60 mL). The organic layer was discarded and the aqueous layer was evaporated to dryness. The residues were washed with cold chloroform for three times and dried under vacuum, the obtained pale yellow solids were directly used for the next step (15.43 g, yield: 85.73%).

Synthesis of Cbz-Dab(Boc)-OH (Scheme S3). To a stirred solution of Cbz-Dab-OH (15.0 g, 59.46 mmol, 1.0 eq.) and water (300 mL) in an ice-water bath, aqueous NaOH solution (1.0 M) was added dropwise to adjust the solution pH to ~10. Boc anhydride (15.57 g, 71.35 mmol, 1.2 eq.) was added and the reaction mixture was stirred overnight at room temperature. After the reaction reached completion, the solution was adjusted to pH ~3 with 2.0 N HCl and immediately extracted with EA (3 × 200 mL) to avoid possible Boc deprotection. The organic phase was evaporated to dryness under reduced pressure and the residues were further purified with column chromatography on silica gel using PE/EA (2/1, v/v) as the eluent. After drying under vacuum, Cbz-Dab(Boc)-OH was obtained as a thick solid (18.52 g, yield: 88.39%), which was directly used for the next step.

Synthesis of H-Dab(Boc)-OH (Scheme S3). Into the solution of Cbz-Dab(Boc)-OH (15.0 g, 42.57 mmol, 1.0 eq.) in methanol (300 mL), Pd/C (1.5 g) was added. The reaction flask was briefly taken into medium vacuum and charged with N_2, and the vacuuming-charging cycle was repeated for three times to remove residual air in the bottle. Next, the reaction mixture was charged with H_2. The mixture was allowed to stir at room temperature, and the progress of the reaction was monitored by TLC. Upon completion, the mixture was filtered through diatomite filter pad and the filter cake was washed with MeOH (2 × 100 mL). The filtrates were combined and evaporated into dryness; after further drying in a vacuum oven, the target compound, H-Dab(Boc)-OH, was obtained as white powder (7.88 g, yield: 84.8%), which was directly used for the next step.

Synthesis of BocDab Precursor (Scheme S3). Into a mixture of H-Dab(Boc)-OH (3 g, 13.75 mmol, 1 eq.), deionized water (30 mL), and sodium carbonate (1.40 g, 13.75 mmol, 1.0 eq.) thermostated at 40 ^{o}C, the solution of (*S*)-1,3-benzothiazol-2-yl-*O*-phenylthiocarbonate

(4.35 g, 15.13 mmol, 1.1 eq.) in THF (90 mL) was added dropwise. The mixture was vigorously stirred and the reaction progress was monitored by TLC (EA, R_f = 0.65). After 3 h, the reaction mixture was diluted with 100 mL aqueous sodium bicarbonate (20 wt%). The organic solvent was then removed by rotary evaporation and the precipitates were filtered off. Next, the aqueous layer was acidified to pH ~3 with 2.0 N HCl and extracted with EA (3 × 200 mL). The organic phase was combined and dried with anhydrous sodium sulfate. After removing all the solvent, the residues were further purified with column chromatography on silica gel using DCM/EA (2/1, v/v) as the eluent. The obtained crude product was recrystallized from *n*-hexane/EA, affording BocDab precursor as white powder (3.95 g, yield: 84.9%). ^{1}H NMR (400 MHz, D_2O, δ, ppm, Figure S6(a)): 12.72 (s, 1H, -COO**H**), 8.09 (d, 1H, -N**H**COO-), 7.38 (t, 2H, Ar**H**), 7.20 (t, 1H, Ar**H**), 7.11 (d, 2H, Ar**H**), 6.89 (t, 1H, -N**H**COO(CH_3)$_3$), 4.02 (s, 1H, -C**H**-), 3.05 (s, 2H, -CH_2C**H**$_2$NHCOO-), 1.73-1.92 (m, 2H, -C**H**$_2$$CH_2$NHCOO-), 1.38 (s, 9H, -(C**H**$_3$)$_3$). ^{13}C NMR (101 MHz, D_2O, δ, ppm, Figure S6(b)): 174.01, 156.05, 154.86, 151.48, 129.74, 125.45, 122.07, 78.06, 52.14, 39.97, 37.35, 31.26, 28.73. ESI-MS (m/z): $[M+Na]^+$ calcd. for $C_{16}H_{23}N_2O_6Na$, 361.13755; found: 361.05096 (Figure S6(c)).

Synthesis of BocO Precursor (Scheme S4). Into a mixture of H-Orn(Boc)-OH (2 g, 8.61 mmol, 1 eq.), deionized water (20 mL), and sodium carbonate (0.912 g, 8.61 mmol, 1.0 eq.) thermostated at 40 °C, the solution of (S)-1,3-benzothiazol-2-yl-O-phenylthiocarbonate (2.72 g, 9.47 mmol, 1.1 eq.) in THF (60 mL) was added dropwise. The mixture was vigorously stirred and the reaction progress was monitored by TLC (EA, R_f = 0.68). After 3 h, the reaction mixture was diluted with 100 mL aqueous sodium bicarbonate (20 wt%). The organic solvent was then removed by rotary evaporation and the precipitates were filtered off. Next, the aqueous layer was acidified to pH ~3 with 2.0 N HCl and extracted with EA (3 × 200 mL). The organic phase was combined and dried with anhydrous sodium sulfate. After removing all the solvent, the residues were further purified with column chromatography on silica gel using DCM/EA (2/1, v/v) as the eluent. The obtained crude product was recrystallized from *n*-hexane/EA, affording BocO precursor as white powder (2.536 g, yield: 83.6%). ^{1}H NMR (400 MHz, DMSO-d_6, δ, ppm, Figure S7(a)): 12.69 (s, 1H, -COO**H**), 8.08 (d, 1H, -N**H**COO-), 7.38 (t, 2H, Ar**H**), 7.23 (t, 1H, Ar**H**), 7.08 (d, 2H, Ar**H**), 6.84 (t, 1H, -N**H**COO(CH_3)$_3$), 3.96 (m, 1H,-C**H**-), 2.93 (m, 2H, -C**H**$_2$NHCOO-), 1.48-1.75 (m, 4H, -C**H**$_2$C**H**$_2$$CH_2$NH-), 1.38 (s, 9H, -(C**H**$_3$)$_3$). ^{13}C NMR (101 MHz, Methanol-d_4, δ, ppm, Figure S7(b)): 174.12, 157.20, 155.72, 151.14, 128.92, 125.00, 121.39, 78.57, 53.93, 39.45, 28.58, 27.39, 26.14. ESI-MS (m/z): $[M+Na]^+$ calcd. for $C_{17}H_{25}N_2O_6$, 375.15320; found: 375.10407 (Figure S7(c)).

Synthesis of CbzK Precursor (Scheme S4). Into a mixture of H-Lys(Cbz)-OH (10.0 g, 35.67

mmol, 1.0 eq.), deionized water (80 mL), and sodium carbonate (3.78 g, 35.67 mmol, 1.0 eq.) thermostated at 40 ^{o}C, the solution of (S)-1,3-benzothiazol-2-yl-O-phenylthiocarbonate (11.28 g, 39.24 mmol, 1.1 eq.) in THF (240 mL) was added dropwise. The mixture was vigorously stirred and the reaction progress was monitored by TLC (EA, R_f = 0.6). After 2 h, the reaction mixture was diluted with 300 mL aqueous sodium bicarbonate (20 wt%). The organic solvent was then removed by rotary evaporation and the precipitates were filtered off. Next, the aqueous layer was acidified to pH ~3 with 2.0 N HCl and extracted with EA (3 × 300 mL). The organic phase was combined and dried with anhydrous sodium sulfate. After removing all the solvent, the residues were further purified with column chromatography on silica gel using DCM/EA (2/1, v/v) as the eluent. The obtained crude product was recrystallized from *n*-hexane/EA, affording CbzK precursor as white powder (12.38 g, yield: 86.7%). ^{1}H NMR (400 MHz, DMSO-d_6, δ, ppm, Figure S8(a)): 12.72 (s, 1H, -COO**H**), 8.09 (s, 1H, -N**H**COOPh), 7.64-7.28 (m, 9H, Ar**H**), 7.21 (t, 1H, -N**H**COOCH$_2$Ph), 7.13-7.02 (m, 2H, Ar**H**), 5.01 (s, 2H, -COOC**H**$_2$-), 3.94 (m, 1H, -(COOH)C**H**-), 3.00 (m, 2H, -C**H**$_2$NHCOO-), 1.27-1.79 (m, 6H, -C**H**$_2$C**H**$_2$C**H**$_2$CH$_2$NHCOO-). ^{13}C NMR (101 MHz, MeOD, δ, ppm, Figure S8(b)): 174.10, 156.56, 154.92, 151.43, 137.73, 129.76, 128.82, 128.20, 125.46, 122.09, 65.59, 54.47, 40.59, 39.34, 30.84, 29.45, 23.38. ESI-MS (m/z): $[M+Na]^+$ calcd. for $C_{21}H_{24}N_2O_6Na$, 423.1532; found: 423.1538 (Figure S8(c)).

Other NPCA precursors including CbzO (85.1 % yield), Phe (85.3% yield), Trp (81.2% yield), and BocK (82.8% yield) were also synthesized according to similar procedures by using (*S*)-1,3-benzothiazol-2-yl-*O*-phenylthiocarbonate as the key intermediate (Figure 1(b) and Scheme S4).

Synthesis of n-BuNH$_3^+$BF$_4^-$ Initiator. *n*-Butylamine (200 mg, 27.3 mmol, 1.0 eq.) was dissolved in 1 mL diethyl ether, tetrafluoroboric acid diethyl ether complex, $HBF_4{\cdot}Et_2O$ (442.8 mg, 2.73 mmol, 1 eq.) was then added, leading to the formation of a white solid. The crude product was collected by filtration and recrystallized twice from ethyl acetate. After drying under high vacuum, the product was obtained as a white solid (232.9 mg, yield: 53.0%) and stored at -20 °C. ^{1}H NMR (400 MHz, DMSO-d_6, δ, ppm, Figure S12(a)): 7.58 (s, 3H, -CH$_2$N**H**$_3^+$BF$_4^-$), 2.78 (s, 2H, -C**H**$_2$NH$_3^+$BF$_4^-$), 1.50 (m, 2H, -C**H**$_2$CH$_2$CH$_3$), 1.31 (m, 2H, -C**H**$_2$CH$_3$), 0.87 (t, 3H, -C**H**$_3$). ^{13}C NMR (101 MHz, DMSO-d_6, δ, ppm, Figure S12(b)): 39.56, 30.03, 20.02, 14.44. ^{19}F NMR (376 MHz, DMSO-d_6, δ, ppm, Figure S12(c)): -143.48.

Synthesis of n-BuNH$_3^+$PF$_6^-$ Initiator. n-Butylamine (2.0 g, 27.3 mmol, 1.0 eq.) was added into a reaction flask. After cooling in an ice-water bath, HPF_6 (60 wt% in water) (7.97g, 32.76

mmol, 1.2 eq.) was added dropwise. After stirring at room temperature for 2 h, the solvent was removed under reduced pressure, affording the crude product as a white solid. After dissolving in methanol (5 mL), an excess of diethyl ether (40 mL) was added. The mixture was allowed to cool down to -20 ^{o}C. After suction filtration, the filter cake was washed with cold diethyl ether (3 × 20 mL), affording the product as a white solid (4.25 g, yield: 75.3%). ^{1}H NMR (400 MHz, DMSO-*d*$_6$, δ, ppm, Figure S13(a)): 5.43 (broad, 3H, -$CH_2N\mathbf{H}_3^+PF_6^-$), 2.68 (t, 2H, -$C\mathbf{H}_2NH_3^+PF_6^-$), 1.45 (m, 2H, -$C\mathbf{H}_2CH_2CH_3$), 1.30 (m, 2H, -$C\mathbf{H}_2CH_3$), 0.88 (t, 3H, -$C\mathbf{H}_3$). ^{13}C NMR (101 MHz, DMSO-*d*$_6$, δ, ppm, Figure S13(b)): 39.99, 31.24, 19.68, 14.07. ^{19}F NMR (376 MHz, DMSO-*d*$_6$, δ, ppm, Figure S13(c)): -69.74, -71.67.

Synthesis of n-BuNH$_3^+$ClO$_4^-$ Initiator. *n*-Butylamine (2.0 g, 27.3 mmol, 1.0 eq.) was added into a reaction flask. After cooling to 0 ^{o}C in an ice-water bath, $HClO_4$ (70 wt% in water) (4.70 g, 32.76 mmol, 1.2 eq.) was added dropwise. The mixture was stirred at room temperature for 2 h. The solvent was removed under reduced pressure affording the crude product as a white solid. After dissolving in methanol, an excess of diethyl ether was added. The mixture was further cooled to -20 ^{o}C, and subsequent suction filtration and washing with cold diethyl ether afforded the target product as a white solid (3.68 g, yield: 77.6%). ^{1}H NMR (400 MHz, DMSO-*d*$_6$, δ, ppm, Figure S14(a)): 7.58 (s, 3H, -$N\mathbf{H}_3^+ClO_4^-$), 2.78 (t, 2H, $C\mathbf{H}_2NH_3^+ClO_4^-$), 1.50 (m, 2H, -$C\mathbf{H}_2CH_2CH_3$), 1.33 (m, 2H, -$C\mathbf{H}_2CH_3$), 0.89 (t, 3H, -$C\mathbf{H}_3$). ^{13}C NMR (101 MHz, DMSO-*d*$_6$, δ, ppm, Figure S14(b)): 39.03, 29.48, 19.46, 13.89.

Synthesis of n-BuNH$_3^+$Br$^-$ Initiator. *n*-Butylamine (2.0 g, 27.3 mmol, 1.0 eq.) was added into a reaction flask and stirred in an ice-water bath. HBr (2.65 g, 32.76 mmol, 1.0 eq.) was added dropwise. The reaction mixture was then stirred at room temperature for 2 h. After removing all the solvent under reduced pressure, the white crude residues were dissolved in methanol and precipitated into an excess of diethyl ether at -20 ^{o}C. After suction filtration, the filter cake was washed with cold diethyl ether (3 × 20 mL), affording the target product as a white solid (2.5 g, yield: 59.5%). ^{1}H NMR (400 MHz, DMSO-*d*$_6$, δ, ppm, Figure S15(a)): 7.77 (s, 3H, -$N\mathbf{H}_3^+Br^-$), 2.76 (t, 2H, -$C\mathbf{H}_2NH_3^+Br^-$), 1.53 (m, 2, -$C\mathbf{H}_2CH_2CH_3$), 1.32 (m, 2H, -$C\mathbf{H}_2CH_3$), 0.87 (t, 3H, -$C\mathbf{H}_3$). ^{13}C NMR (101 MHz, DMSO-*d*$_6$, δ, ppm, Figure S15(b)): 38.97, 29.44, 19.58, 13.96.

Typical Polymerization Procedures of NPCA Precursors Initiated by Protonated Amine in Glove Box. All NPCA precursors and initiators were vacuum dried at room temperature for at least 24 h and stored in N_2 atmosphere prior to use. As a typical example, procedures employed for the polymerization of NBO precursor using *n*-BuNH$_3^+$Cl$^-$ initiator in a nitrogen-purged glovebox are described below. NBO precursor was placed in a vial and protonated amine

initiator was added at varying $[M]_0/[I]_0$ molar ratios. Next, DMAc was added to maintain a constant $[M]_0$ of 0.25 M. The reaction mixture was stirred at 70 °C in glovebox for varying time durations. Taking the case of $[M]_0/[I]_0$ ratio of 100 as an example, NBO precursor (100 mg, 0.23 mmol, 100 eq.) was placed in a vial and *n*-$BuNH_3^+Cl^-$ initiator (6 mg/mL in DMAc) (42.4 μL, 0.0023 mmol, 1.0 eq.) was added. DMAc (860 μL) was then added and the reaction mixture was stirred for 72 h at 70 °C in glove box. The NPCA conversion was assayed by ^1H NMR in DMSO-d_6. After the polymerization reached completion, the solution mixture was precipitated into an excess of cold diethyl ether and dried in a vacuum oven, affording the target PNBO polypeptide. The DPs of PNBO were calculated based on the ratio of ^1H NMR peak integration of methylene protons of oNB group (~5.1-5.4 ppm) relative to that of terminal methyl protons ascribing to *n*-$BuNH_3^+Cl^-$ initiator (~0.8 ppm; refer to Figure S20(b) for details). The DPs of other polypeptides were calculated according to similar procedures.

Kinetics Study of NPCA Polymerization Initiated by n-$BuNH_3^+Cl^-$. In the nitrogen-purged glovebox, NBO precursor (100 mg, 0.23 mmol, 80.0 eq.) was placed in a glass vial and *n*-$BuNH_3^+Cl^-$ (6 mg/mL in DMAc) (52.9 μL, 0.0029 mmol, 1.0 eq.) was added. DMAc (850 μL) was then added to reach an initial monomer concentration, $[M]_0$, of 0.25 M. The reaction mixture was stirred at 70 °C in the glovebox. During polymerization, 50 μL aliquot of the reaction mixture was sampled out, ~20 μL was immediately diluted with DMSO-d_6 to determine monomer conversion via ^1H NMR analysis; the remaining portion was diluted with the mobile phase of GPC (DMF), filtered through 220 nm membrane, and directly subjected to GPC analysis to determine M_n and polydispersity index (M_w/M_n or $Đ$) without further purification. Typical sampling time points were set as follows: 0 h, 13 h, 15 h, 19 h, 24 h, 27 h, 32 h, 41 h, 48 h, 56 h, 67 h, 72 h, and 96 h.

According to ^1H NMR spectra shown in Figure 2(b) and Figure S11, the kinetics of NPCA polymerization including extents of NPCA consumption, NCA formation, and polypeptide formation could be calculated. Note that peaks a, c, and d in the range of 5.12-5.43 ppm are ascribed to methylene protons of oNB residues in NPCA, NCA, and polypeptide, and their total integration does not change during polymerization and could be used as an internal standard. The appearance of phenol signal (peaks e-h; peaks e and f at ~6.7 ppm was used for calculation) indicates the consumption of NPCA monomer and transformation into NCA monomer. Note that NCA will be further polymerized into polypeptide, and the instantaneous NCA concentration, $[NCA]_t$, could be quantified from peak b at ~4.5 ppm. Relevant calculation protocols are as follows, and "I" refers to the integration area of given NMR resonance peaks.

$$\text{Consumed NPCA} = [\text{NCA}]_t + \text{Formed polypeptide}$$

$$\text{Formed polypeptide} = \text{Polymerized NPCA} = [\text{Polypeptide}]_t$$

$$\text{Consumed NPCA} = \frac{[\text{NPCA}]_0 - [\text{NPCA}]_t}{[\text{NPCA}]_0} = \frac{\text{I(e + f)}/3}{\text{I(a + c + d)}/2} = \frac{2\ \text{I(e + f)}}{3\ \text{I(a + c + d)}}$$

$$\frac{[\text{NCA}]_t}{[\text{NPCA}]_0} = \frac{2\ \text{I(b)}}{\text{I(a + c + d)}}$$

$$\frac{[\text{NPCA}]_t}{[\text{NPCA}]_0} = 1 - \frac{2\ \text{I(e + f)}}{3\ \text{I(a + c + d)}}$$

$$[\text{Polypeptide}]_t = \text{Consumed NPCA} - [\text{NCA}]_t$$

$$= \frac{[\text{NPCA}]_0 - [\text{NPCA}]_t}{[\text{NPCA}]_0} - \frac{[\text{NCA}]_t}{[\text{NPCA}]_0}$$

$$= \frac{2\ \text{I(e + f)} - 6\ \text{I(b)}}{3\ \text{I(a + c + d)}}$$

Comparison of NPCA Polymerizations using n-BuNH$_3^+$Cl$^-$ versus n-BuNH$_2$ as Initiators. All NPCA precursors were vacuum dried at room temperature for at least 24 h and stored in N_2 atmosphere prior to use. In a nitrogen-purged glovebox, NPCA precursor was placed in a glass vial and *n*-BuNH$_3^+$Cl$^-$ (6 mg/mL in DMAc) or *n*-BuNH$_2$ (6 mg/mL in DMAc) was added to obtain varying $[M]_0/[I]_0$ feed ratios. DMAc was then added to reach an $[M]_0$ of 0.25 M. The reaction mixture was stirred at 60 °C for primary amine and 70 °C for primary amine hydrochloride in the glovebox. The extent of NPCA consumption and polypeptide conversion was measured by ^{1}H NMR in DMSO-d_6. After the polymerization was completed, the polypeptide products were obtained by precipitation into an excess of cold diethyl ether and drying in a vacuum oven, which were subjected to further GPC and MALDI-TOF MS analysis.

*Polymerizations of NPCA Precursors using n-BuNH$_2$ Initiator in the Presence of Acetic **Acid.*** All NPCA precursors were vacuum dried at room temperature for at least 24 h and stored in N_2 atmosphere prior to use. In a nitrogen-purged glove box, NPCA precursor was placed in a glass vial and *n*-BuNH$_2$ (6 mg/mL in DMAc) was added to obtain varying $[M]_0/[I]_0$ ratios. Acetic acid (AA) was added to maintain a molar ratio of [AA]/[*n*-BuNH$_2$] = 10/1. DMAc was then added to reach an $[M]_0$ of 0.25 M. The reaction mixture was stirred at 60 °C in a glove box. The extents of NPCA consumption and polypeptide conversion were measured by ^{1}H NMR in DMSO-d_6. After the polymerization was completed, the polypeptide product was obtained by precipitation into an excess of diethyl ether and dried in a vacuum oven, which was subjected to further GPC and MALDI-TOF MS analysis. Note that the initiator, *n*-BuNH$_2$, is in the form of deprotonated primary amine, which is unstable in open air and prone to deteriorate. Thus, the polymerization procedures need to be conducted under inert gas atmosphere.

Polymerization of NPCA Precursors Initiated by n-BuNH$_3^+$Cl$^-$ in Open Vessel Exposed to

Air. The open-vessel polymerization was carried out in a general chemical laboratory with relatively high seasonal humidity (relative humidity >80%; see the hygrometer in Figure 4(a) for details). NPCA precursor was placed in a glass vial and *n*-$BuNH_3^+Cl^-$ (6 mg/mL in DMAc) was added at varying $[M]_0/[I]_0$ ratios. DMAc was then added to reach an initial monomer concentration, $[M]_0$, of 0.25 M. The reaction mixture was directly exposed to air (i.e., no stopper, without inert gas protection) and stirred at 70 ^oC in the fume hood. The extents of NPCA consumption and polypeptide conversion were measured by ^1H NMR in DMSO-d_6. After completion of polymerization, the reaction mixture was precipitated into an excess of diethyl ether and further drying in a vacuum oven afforded the target polypeptide product.

Analysis of the Evolution of Water Contents during Open-Vessel Polymerization. The analysis of water contents during open-vessel NBO polymerization was further conducted to verify that primary amine hydrochloride-initiated NPCA polymerization is moisture-tolerant to some extent and could avoid the use of strictly anhydrous solvents and reagents. NBO (100 mg, 0.2318 mmol, 10 eq.) was placed in a glass vial and *n*-$BuNH_3^+Cl^-$ (2.54 mg, 0.02318 mmol, 1 eq.; 6 mg/mL in DMAc) was added. DMAc was then added to reach an initial monomer concentration, $[M]_0$, of 0.25 M. The reaction mixture was directly exposed to air (i.e., no stopper, without protection of inert gas atmosphere) and stirred at 70 ^oC for 24 h in the fume hood. During polymerization, an aliquot of 60 μL was taken at specified time intervals (0 min, 10 min, 30 min, 1 h, 4 h, 9 h, and 20 h) into NMR tube, sealed, and immediately placed inside N_2-purged glove box. 600 μL DMSO-d_6 was added into the NMR tube and sealed for further ^1H NMR test. The quality and water content in DMSO-d_6 solvent (0.6 mL package sealed in individual ampules) were verified by ^1H NMR before measurements. At specified time intervals, another aliquot of the reaction mixture (~60 μL) was taken, precipitated into diethyl ether, dried under reduced pressure, and subjected for further MALDI-TOF MS analysis. After completion of polymerization, the reaction mixture was precipitated into an excess of diethyl ether and further dried in a vacuum oven, affording the target polypeptide product as solids.

Tracking the Evolution of n-$BuNH_3^+Cl^-$-Initiated NPCA Polymerization under Glove Box and Open-Vessel Conditions using MALDI-TOF MS. Detailed procedures of CbzK polymerization under open-vessel conditions are as follows. Inside the fume hood, CbzK precursor (150 mg, 0.3746 mmol, 35 eq.) was placed in a glass vial and *n*-$BuNH_3^+Cl^-$ (1.174 mg, 0.0107 mmol, 1 eq.; 6 mg/mL in DMAc) was added. DMAc solvent was added to target an initial monomer concentration, $[M]_0$, of 0.25 M. The reaction mixture was directly exposed to air (i.e., no stopper, without protection of inert gas atmosphere) and stirred at 70 ^oC. During polymerization, an aliquot of 60 μL at specific time interval (12 h, 15 h, 18 h, 20 h, 24 h, 30 h,

and 36 h) was taken, precipitated into diethyl ether, and dried under reduced pressure for further MALDI-TOF MS analysis. According to similar procedures, the above polymerization protocols were also conducted inside a nitrogen-purged glove box, and aliquots of the reaction mixture were taken at specified time intervals, precipitated into diethyl ether, dried under reduced pressure, and subjected to further MALDI-TOF MS analysis.

One-Pot Synthesis of Random Copolypeptides under Open-Vessel Conditions using n-BuNH$_3^+$Cl$^-$ Initiator. Typical procedures for random copolymerization of NPCAs including NBO, NBK, Phe, CbzK, and BocK are as follows. NPCA precursors were placed in a glass vial and *n*-BuNH$_3^+$Cl$^-$ (6 mg/mL in DMAc) were added to reach varying $[M]_0/[I]_0$ ratios. DMAc was added to reach a total monomer concentration, $[M]_0$, of 0.25 M. The reaction mixture was directly exposed to air (i.e., no stopper, without any protection of inert gas) and stirred at 70 ^oC in the fume hood. The extents of NPCA consumption and polypeptide formation were measured by ^1H NMR in DMSO-d_6. After completion of polymerization, the reaction mixture was precipitated into an excess of diethyl ether and further drying in a vacuum oven afforded the target copolypeptide product.

For the synthesis of poly(BocK$_{0.23}$-*r*-NBO$_{0.54}$-*r*-Phe$_{0.23}$)$_{22}$, NBO (50 mg, 0.1159 mmol, 11 eq.), Phe (13.83 mg, 0.04847 mmol, 4.6 eq.), and BocK (16.987 mg, 0.04636 mmol, 4.4 eq.) were added into the reaction vial. *n*-BuNH$_3^+$Cl$^-$ (1.155 mg, 0.01053 mmol, 1 eq.; 6 mg/mL in DMAc) and DMAc were then added to reach a total monomer concentration, $[M]_0$, of 0.25 M. The reaction mixture was directly exposed to air and stirred at 70 ^oC for 24 h in the fume hood. After completion of polymerization, the reaction mixture was precipitated into an excess of diethyl ether and further drying in a vacuum oven afforded the target polypeptide product.

According similar procedures, poly(BocK$_{0.39}$-*r*-NBO$_{0.42}$-*r*-Phe$_{0.19}$)$_{31}$ and poly(BocK$_{0.31}$-*r*-NBO$_{0.47}$-*r*-Phe$_{0.22}$)$_{51}$, Poly(BocK$_{0.20}$-*r*-NBO$_{0.47}$-*r*-Phe$_{0.33}$)$_{70}$, and poly(BocK$_{0.25}$-*r*-NBK$_{0.2}$-*r*-Phe$_{0.21}$-*r*-Trp$_{0.34}$)$_{100}$ copolypeptides were also synthesized.

Preparation of Diblock and Triblock Copolypeptides by Sequential NPCA Polymerizations under Open-Vessel Condition using n-BuNH$_3^+$Cl$^-$ Initiator. Detailed procedures employed for sequential block copolymerization of NPCAs including Trp, NBO, and CbzK are as follows. NPCA precursors were respectively dissolved in DMAc to reach a concentration of 0.25 M and used as stock solution. The NPCA stock solution for the first block and *n*-BuNH$_3^+$Cl$^-$ were charged into the reaction flask. The reaction mixture was directly exposed to air (i.e., no stopper, without protection of inert gas atmosphere) and stirred at 70 ^oC in the fume hood. Upon completion of polymerization for the first block, an aliquot of the reaction mixture was sampled out for ^1H NMR and GPC analysis. The NPCA stock solution for the second block was then

added, and the chain extension process was conducted at 70 ^{o}C under open-vessel condition. Upon completion of diblock and triblock copolymerization, the reaction mixture was precipitated into an excess of diethyl ether and further drying in a vacuum oven afforded the copolypeptide products. Polymerization times, conversions, and structural parameters of the obtained block copolypeptides are summarized in Table 1.

Gram Scale Synthesis of Polypeptide under Open-Vessel Condition using n-BuNH$_3^+$Cl$^-$ Initiator. Taking the synthesis of PCbzK$_{30}$ as a typical example, CbzK precursor (1.5 g, 3.74 mmol, 30 eq.) was charged into a glass vial and *n*-BuNH$_3^+$Cl$^-$ (2.28 mL, 6 mg/mL in DMAc, 0.1249 mmol, 1 eq.) was added. DMAc (12.68 mL) was then added to reach a monomer concentration, $[M]_0$, of 0.25 M. The reaction mixture was directly exposed to air and stirred at 70 ^{o}C in the fume hood. The extents of NPCA consumption and polypeptide formation were measured by ^{1}H NMR in DMSO-d_6. After completion of polymerization, the reaction mixture was precipitated into an excess of diethyl ether and further drying in a vacuum oven afforded the polypeptide product (1.3 g, yield: 86.7%). The obtained polypeptide, PCbzK$_{30}$, was characterized by ^{1}H NMR, GPC, and MALDI-TOF MS measurements.

Characterization

All nuclear magnetic resonance (NMR) spectra were recorded on a Bruker AV400 NMR (400 MHz) spectrometer operated in the Fourier transform mode. Deuterated DMSO (DMSO-d_6), deuterium oxide (D_2O), deuterated chloroform ($CDCl_3$), and methanol-d_4 (CD_3OD) were used as the solvents. GPC measurements were conducted by using both differential refractive index (RI) detector and multi-angle laser light scattering (MALLS) detector. Molecular weights and molecular weight distributions were determined by GPC/MALLS using a SSI pump connected to a Wyatt DAWN HELEOS II light scattering detector and Wyatt Optilab T-rEX RI detector. DMF containing LiBr (10 mM) was used as eluent at a flow rate of 1.0 mL/min. A series of low polydispersity polystyrene standards were employed for calibration. Refractive index increment (dn/dc) values were measured using Wyatt Optilab T-rEX interferometric refractometer operated at a wavelength of 658 nm. Raw data were processed with the Astra V software (Wyatt Technology). The molecular weight and polydispersity index (*Đ*) of PNBO synthesized at $[M]_0/[I]_0$ feed ratios of 200, 400, and 800 were characterized using the MALLS detector, and absolute molecular weights were determined using ASTRA 6.1.1.17 software (Wyatt Technology) assuming 100% mass recovery. The dn/dc value of 0.140 mL·g^{-1} was used for PNBO polypeptide. Other polypeptide samples were characterized using the differential RI detector, unless otherwise noted. Electrospray ionization mass spectrometry (ESI-MS)

experiments were performed on a Thermo Scientific LTQ Orbitrap Mass Spectrometer equipped with an electrospray interface. Matrix-assisted laser desorption/ionization time-of-flight (MALDI-TOF) mass spectra were acquired on an UltrafleXtreme MALDI-TOF mass spectrometer equipped with a 150 kHz smart beam-II laser. All spectra were recorded in positive reflector mode unless otherwise noted. The solution of trans-2-[3-(4-tert-butyl-phenyl)-2-methyl-2-propenylidene]-malononitrile (DCTB, Aldrich, >98%) in $CHCl_3$ at a concentration of 20 mg/mL was used as the matrix. The cationizing agent, sodium trifluoroacetate, was dissolved in ethanol at a concentration of 10 mg/mL. The matrix and cationizing salt solutions were mixed at a ratio of 10/1 (v/v). All polypeptide samples were dissolved in HFIP at a concentration of 10 mg/mL. The "sandwich" technique was used for MALDI-TOF MS sample preparation. 0.5 μL matrix solution was deposited on the wells of a 384-well ground-steel plate; upon brief drying, 0.5 μL of sample solution was deposited on top of the dried matrix; upon solvent evaporation, another 0.5 μL of DCTB solution was deposited. After sample preparation and complete solvent evaporation, the plate was inserted into the MALDI-TOF mass spectrometer. The extent of laser attenuation was adjusted to minimize undesired polymer fragmentation and maximize detection sensitivity. Data analyses were conducted with Bruker's flexAnalysis software. Fourier-transform infrared (FT-IR) spectra were recorded on a Bruker Tensor II IR spectrometer. The spectra were collected over 64 scans with a spectral resolution of 4 cm^{-1}. Circular dichroism (CD) measurements were performed on a Jasco J-1500 circular dichroism spectrometer equipped with a PFD-425S/15 Peltier-type temperature controller, and the temperature was set at 20 ^{o}C. The samples were dissolved in HFIP and diluted to a concentration of 0.05 mg/mL.

Table S1. Controlled synthesis of polypeptides via polymerization of NPCA precursors at 70 ^{o}C initiated by *n*-$BuNH_3^+Cl^-$.

Entry	Monomer	$[M]_0/[I]_0$	Time (h)	$M_{n,NMR}$ (kDa) [a]	Conv. (%) [a]	DP [a]	$M_{n,GPC}$ [b] ($M_{n,MALDI}$ [d]) kDa	$Đ_{GPC}$ [e] ($Đ_{MALDI}$ [f])
**1**	NBO	5	24	1.5	>99	5	1.8 (1.7 [d])	1.03 (1.04 [f])
**2**	NBO	7	24	2.1	>99	7	2.0 (2.2 [d])	1.05 (1.07 [f])
**3**	NBO	10	24	3.0	>99	10	2.8 (2.9 [d])	1.04 (1.03 [f])
**4**	NBO	30	36	9.1	>99	31	8.8	1.09
**5**	NBO	60	60	17.7	>99	60	16.6	1.15
**6**	NBO	80	72	23.5	>99	80	21.5	1.14
**7**	NBO	100	72	29.7	>99	101	27.8	1.13
**8**	NBO	200	72	-	>99	206 [c]	60.4 [c]	1.14
**9**	NBO	400	120	-	>99	406 [c]	119.1 [c]	1.12
**10**	NBO	800	144	-	>99	767 [c]	224.8 [c]	1.15
**11**	CbzO	7	24	2.0	>99	7	1.8 (1.9 [d])	1.04 (1.06 [f])
**12**	CbzO	65	72	16.2	>99	65	15.1	1.12
**13**	CbzO	80	72	20.7	>99	83	19.8	1.16
**14**	CbzO	100	72	25.4	>99	102	23.8	1.12
**15**	Trp	14	24	2.5	92.9	13	0.98 (2.7 [d])	1.03 (1.05 [f])
**16**	Trp	30	36	5.3	93.3	28	4.7	1.14
**17**	Trp	45	48	8.8	>99	47	5.7	1.11
**18**	Trp	90	72	17.4	>99	93	13.6	1.12
**19**	Trp	200	72	37.3	>99	200	27.8	1.09
**20**	BocDab	5	24	1.1	>99	5	1.0 (1.1 [d])	1.03 (1.05 [f])
**21**	BocO	7	24	1.6	>99	7	1.7 (1.6 [d])	1.02 (1.03 [f])
**22**	NBK	10	24	2.8	90.7	9	2.6 (2.8 [d])	1.03 (1.08 [f])
**23**	NBDab	13	24	3.4	93.7	12	3.3 (3.8 [d])	1.04 (1.05 [f])
**24**	NBDab	20	48	5.7	>99	20	5.3	1.04

[a] Calculated from ^1H NMR spectra. [b] Number-average molecular weight, $M_{n,GPC}$, determined by GPC using a refractive index (RI) detector (eluent: DMF, 10 mM LiBr; 1 mL/min). [c] Determined by MALLS-GPC using a light scattering detector, the dn/dc value of 0.140 $mL \cdot g^{-1}$ was used for PNBO polypeptides. [d] Number-average molecular weight, $M_{n,MALDI}$, determined by MALDI-TOF MS. [e] Polydispersity index (M_w/M_n) determined by GPC. [f] Polydispersity index (M_w/M_n) determined by MALDI-TOF MS.

Table S2. Controlled synthesis of polypeptides via polymerization of NBO precursor under varying conditions.

Entry	Initiator	$[M]_0/[I]_0$	Temp. (°C)	Reaction Time (h)	$M_{n,NMR}$ (kDa) [a]	Conv. (%) [a]	$M_{n,GPC}$ (kDa) [b]	$Đ$ [c]
**1**	n-$BuNH_3^+Cl^-$	100	30	72	/[d]	/[d]	/[d]	/[d]
**2**	n-$BuNH_3^+Cl^-$	100	40	72	/[d]	/[d]	/[d]	/[d]
**3**	n-$BuNH_3^+Cl^-$	100	50	72	24.4	82.7	19.5	1.18
**4**	n-$BuNH_3^+Cl^-$	100	60	72	26.7	90.4	21.7	1.27
**5**	n-$BuNH_3^+Cl^-$	100	70	72	29.7	>99	27.8	1.13
**6**	n-$BuNH_3^+Br^-$	100	70	72	15.9	53.9	14.3	1.24
**7**	n-$BuNH_3^+BF_4^-$	100	70	72	28.3	96.0	25.7	1.22
**8**	n-$BuNH_3^+PF_6^-$	100	70	72	27.9	94.8	24.5	1.35
**9**	n-$BuNH_3^+ClO_4^-$	100	70	72	27.1	91.9	24.9	1.27

[a] Calculated from ^{1}H NMR spectra. [b] Determined by GPC using refractive index (RI) detector (eluent: DMF, 10 mM LiBr; 1 mL/min). [c] Polydispersity index (M_w/M_n) determined by GPC. [d] No polypeptide formed under this polymerization temperature.

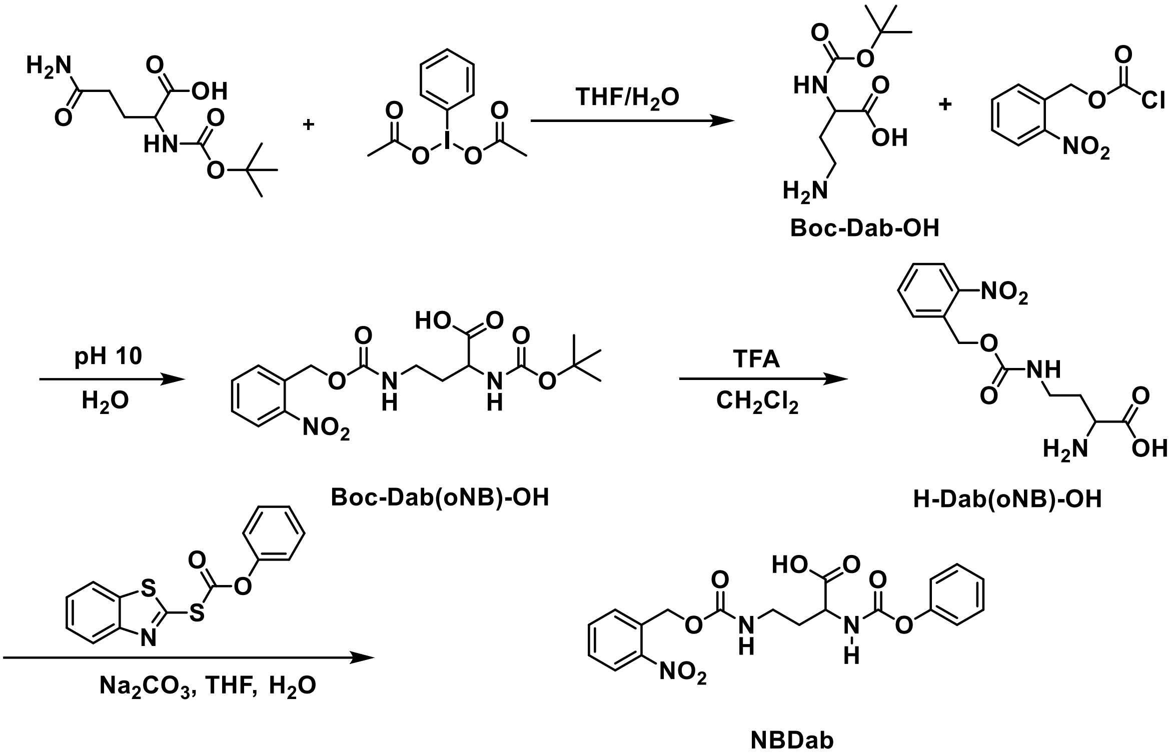

Scheme S1. Synthetic routes employed for the preparation of *N*-phenyloxycarbonyl-functionalized α-amino acid, NBDab.

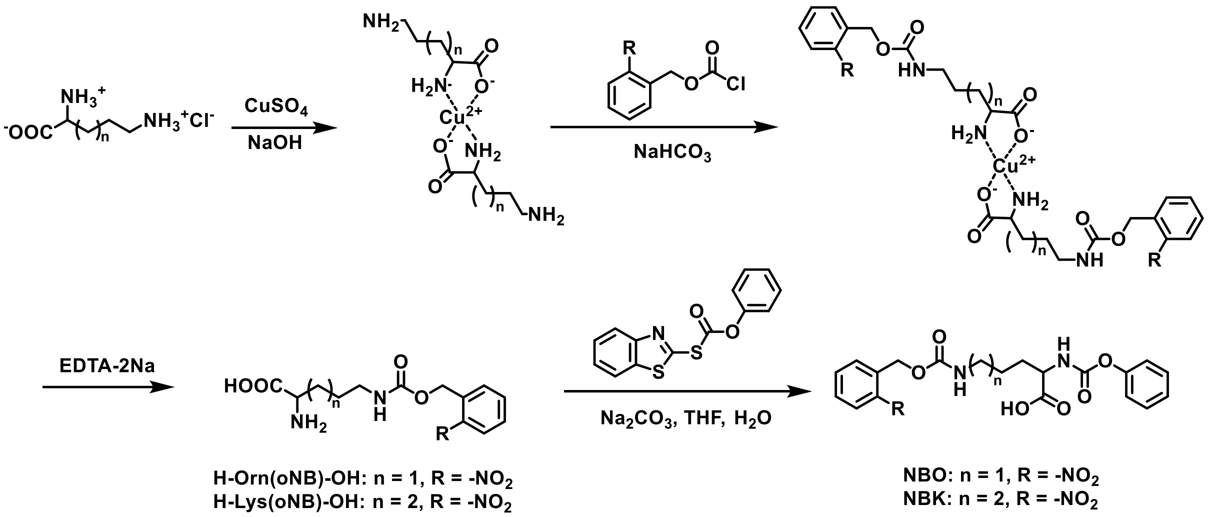

Scheme S2. Synthetic routes employed for the preparation of *N*-phenyloxycarbonyl-functionalized α-amino acids, NBO and NBK.

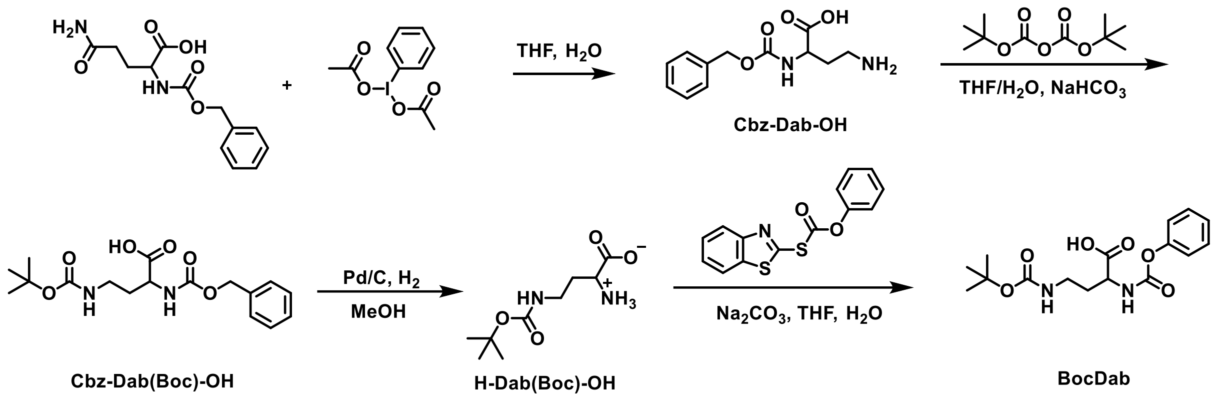

Scheme S3. Synthetic routes employed for the preparation of *N*-phenyloxycarbonyl-functionalized α-amino acid, BocDab.

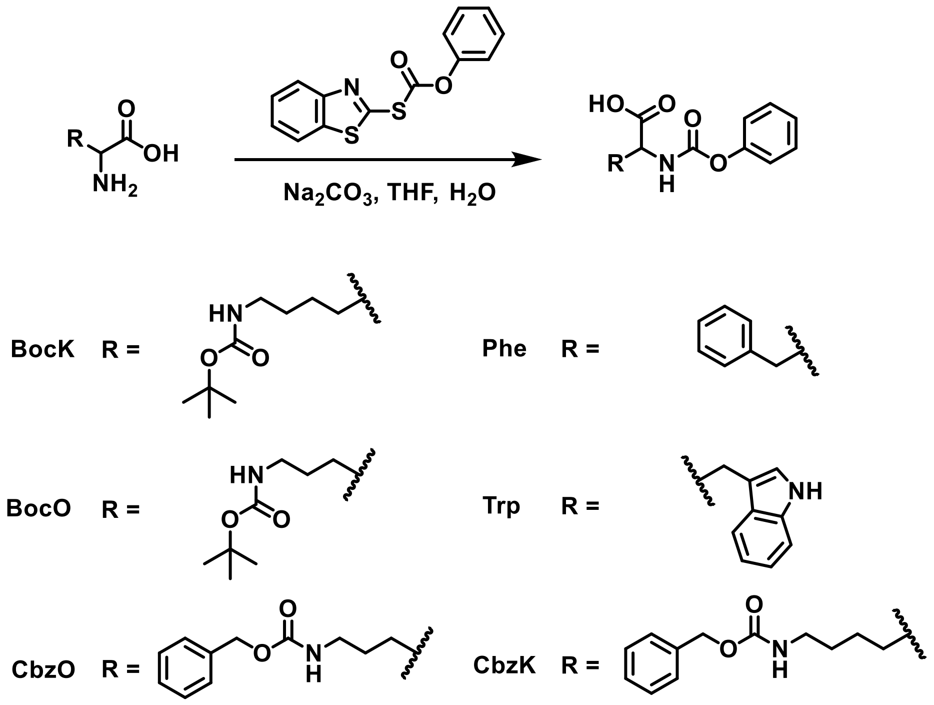

Scheme S4. Synthetic routes employed for the preparation of *N*-phenyloxycarbonyl-functionalized α-amino acids including BocK, BocO, Phe, Trp, CbzO, and CbzK.

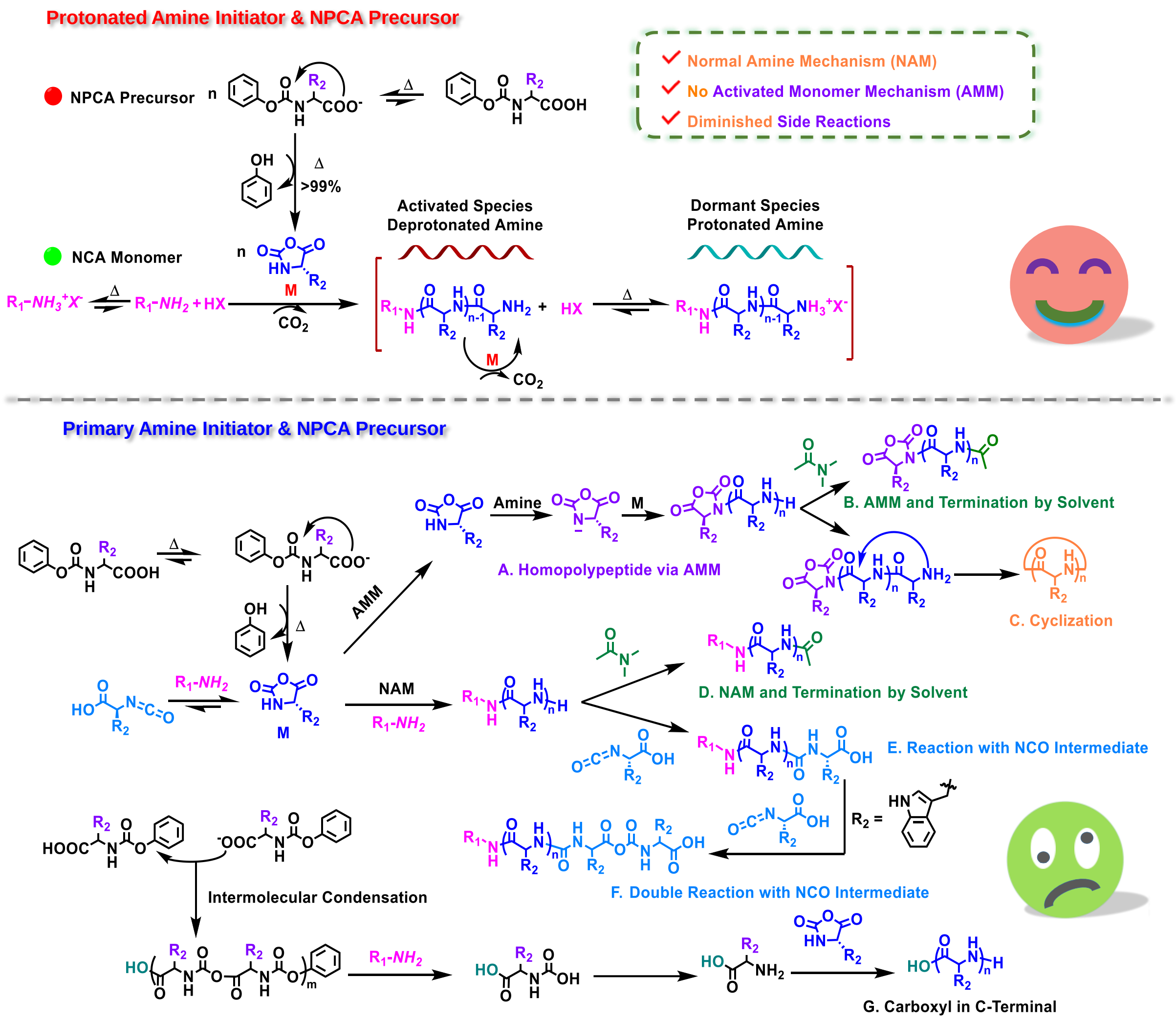

Figure S1. Comparison of the mechanisms of NPCA polymerization using protonated primary amine *versus* conventional primary amine as initiators. Also illustrated are possible side reactions associated with the use of conventional primary amine as initiator.

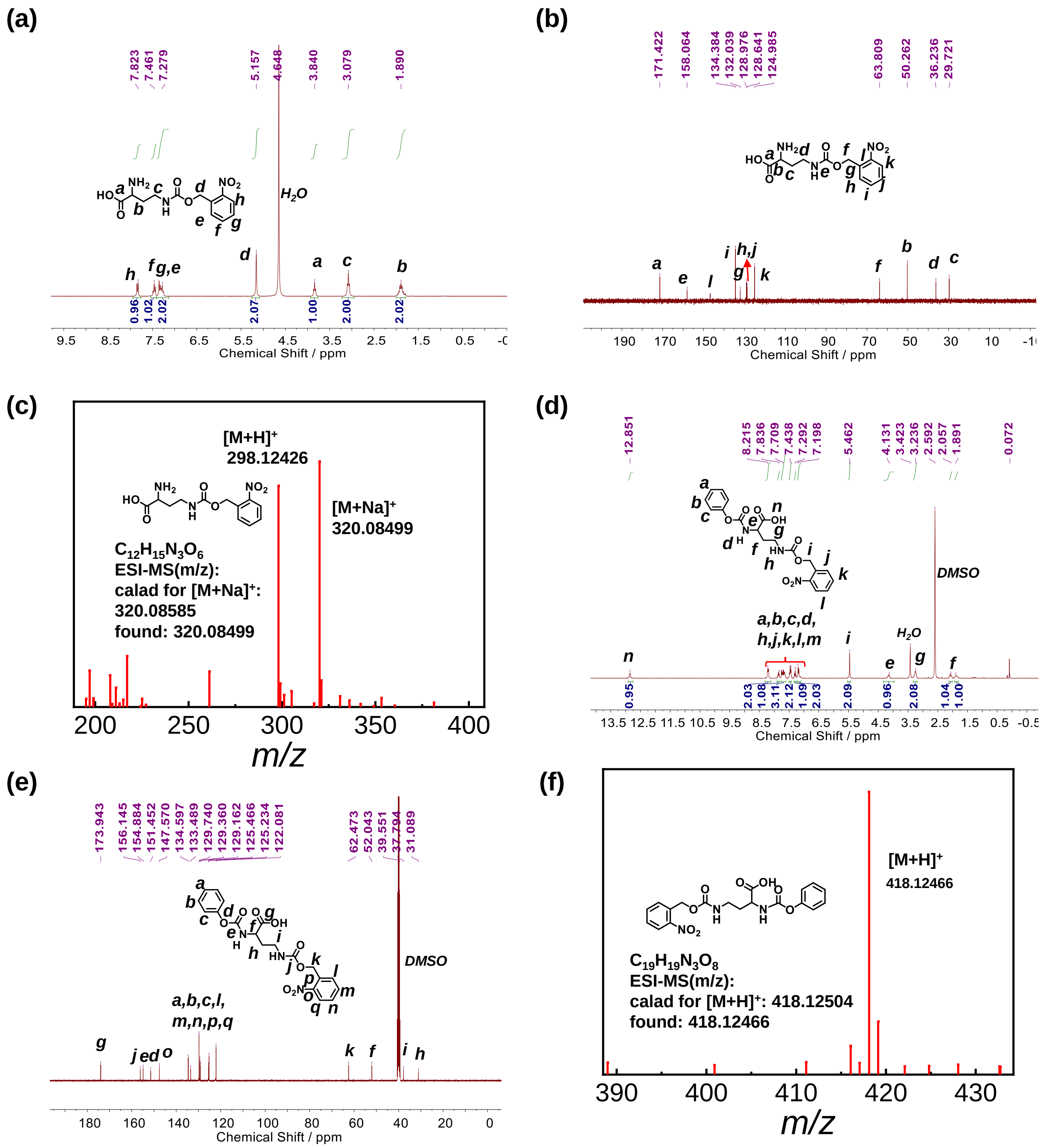

Figure S2. (a) ^{1}H NMR and (b) ^{13}C NMR spectra recorded in D_2O for H-Dab(oNB)-OH. (c) ESI-MS spectrum recorded for H-Dab(oNB)-OH. (d) ^{1}H and (e) ^{13}C NMR spectra recorded in DMSO-d_6 for NBDab. (f) ESI-MS spectrum recorded for NBDab.

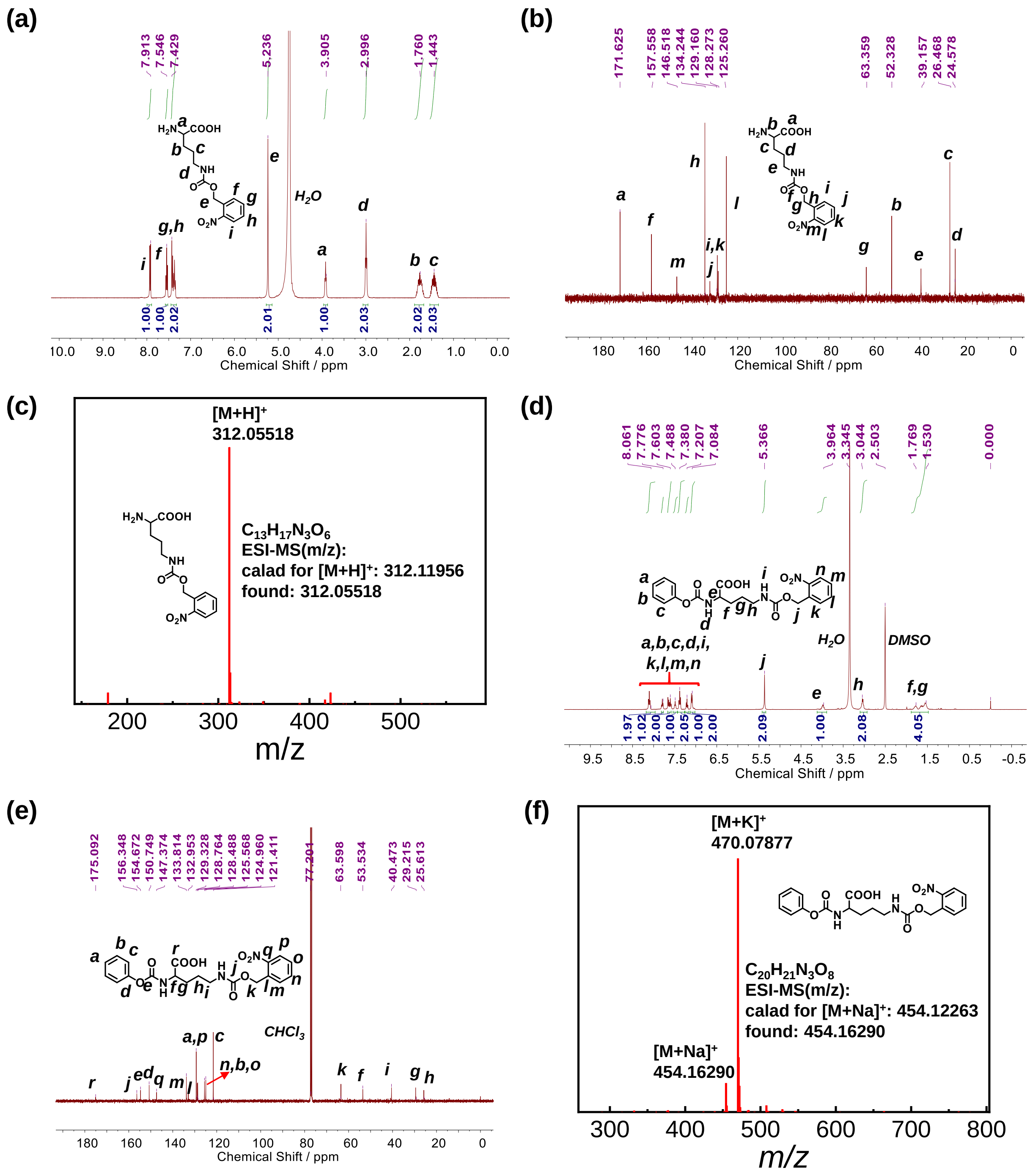

Figure S3. (a) ^{1}H NMR and (b) ^{13}C NMR spectra recorded in D_2O for H-Orn(oNB)-OH. (c) ESI-MS spectrum recorded for H-Orn(oNB)-OH. (d) ^{1}H NMR spectrum recorded in DMSO-d_6 and (e) ^{13}C NMR spectrum recorded in $CDCl_3$ for NBO precursor. (f) ESI-MS spectrum recorded for NBO precursor.

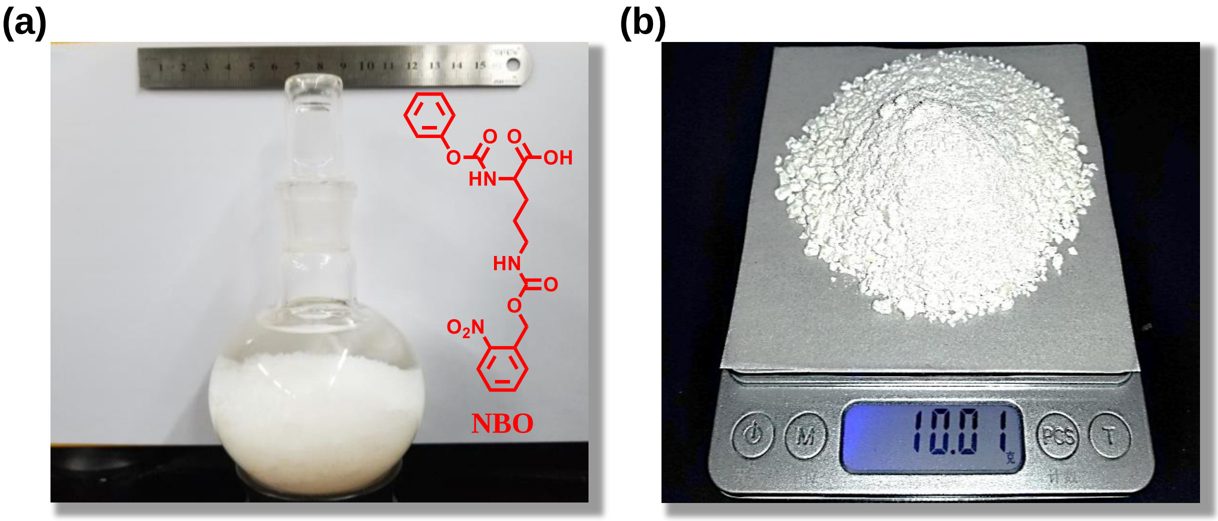

Figure S4. Macroscopic images of moisture-insensitive and air-stable NPCA precursor of functionalized ornithine, NBO, which was synthesized at ~10 g scale and further purified via recrystallization from *n*-hexane/EA mixture.

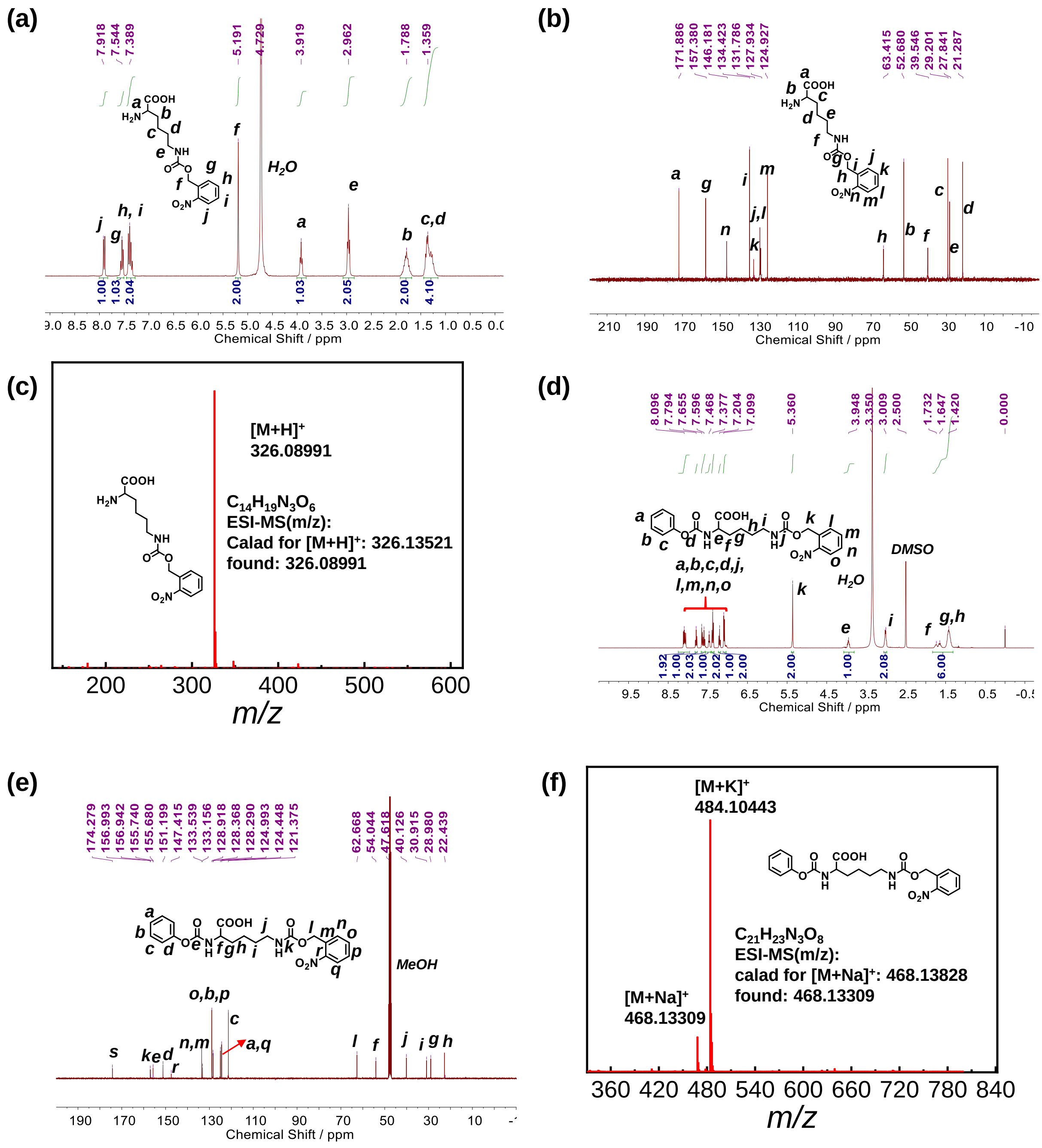

Figure S5. (a) ^{1}H NMR and (b) ^{13}C NMR spectra recorded in D_2O for H-Lys(oNB)-OH. (c) ESI-MS spectrum recorded for H-Lys(oNB)-OH. (d) ^{1}H NMR spectrum recorded in DMSO-d_6 and (e) ^{13}C NMR spectrum recorded in MeOD for NBK precursor. (f) ESI-MS spectrum recorded for NBK precursor.

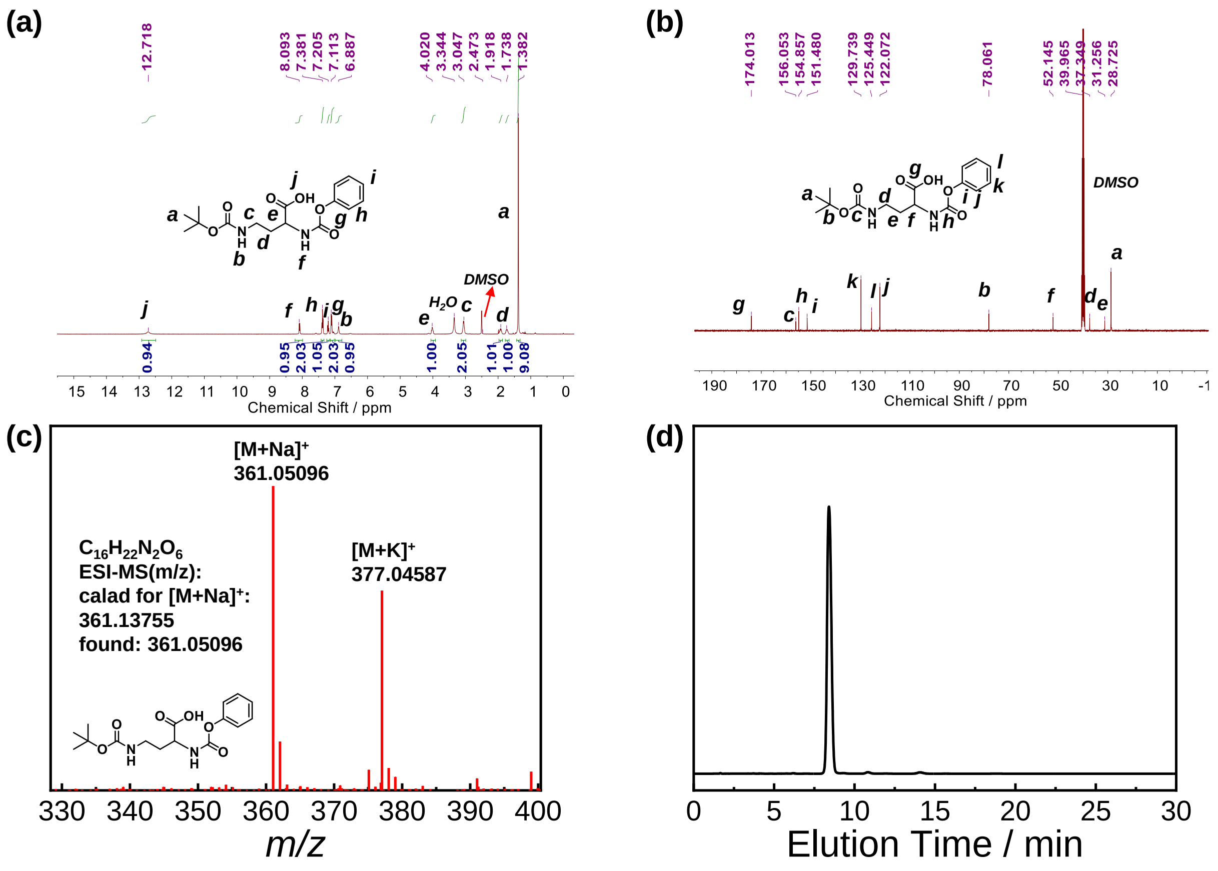

Figure S6. (a) ^{1}H NMR and (b) ^{13}C NMR spectra recorded in DMSO-d_6 for BocDab precursor. (c) ESI-MS spectrum recorded for BocDab precursor. (d) HPLC profile (MeCN/H_2O, 4/6 v/v; 0.1% TFA) recorded for BocDab precursor (274 nm absorbance).

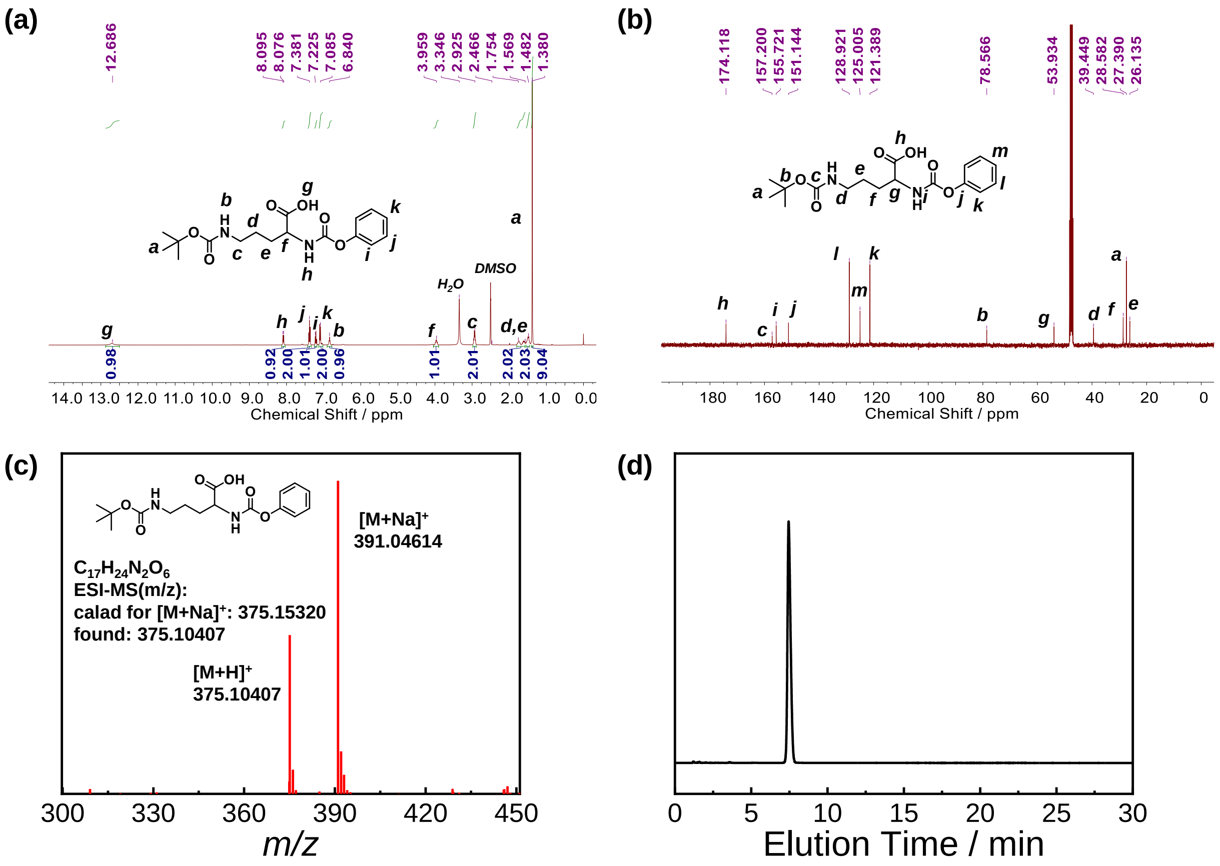

Figure S7. (a) ^{1}H NMR and (b) ^{13}C NMR spectra recorded in DMSO-d_6 for BocO precursor. (c) ESI-MS spectrum recorded for BocO precursor. (d) HPLC profile (MeCN/H_2O, 4/6 v/v; 0.1% TFA) recorded for BocO precursor (254 nm absorbance).

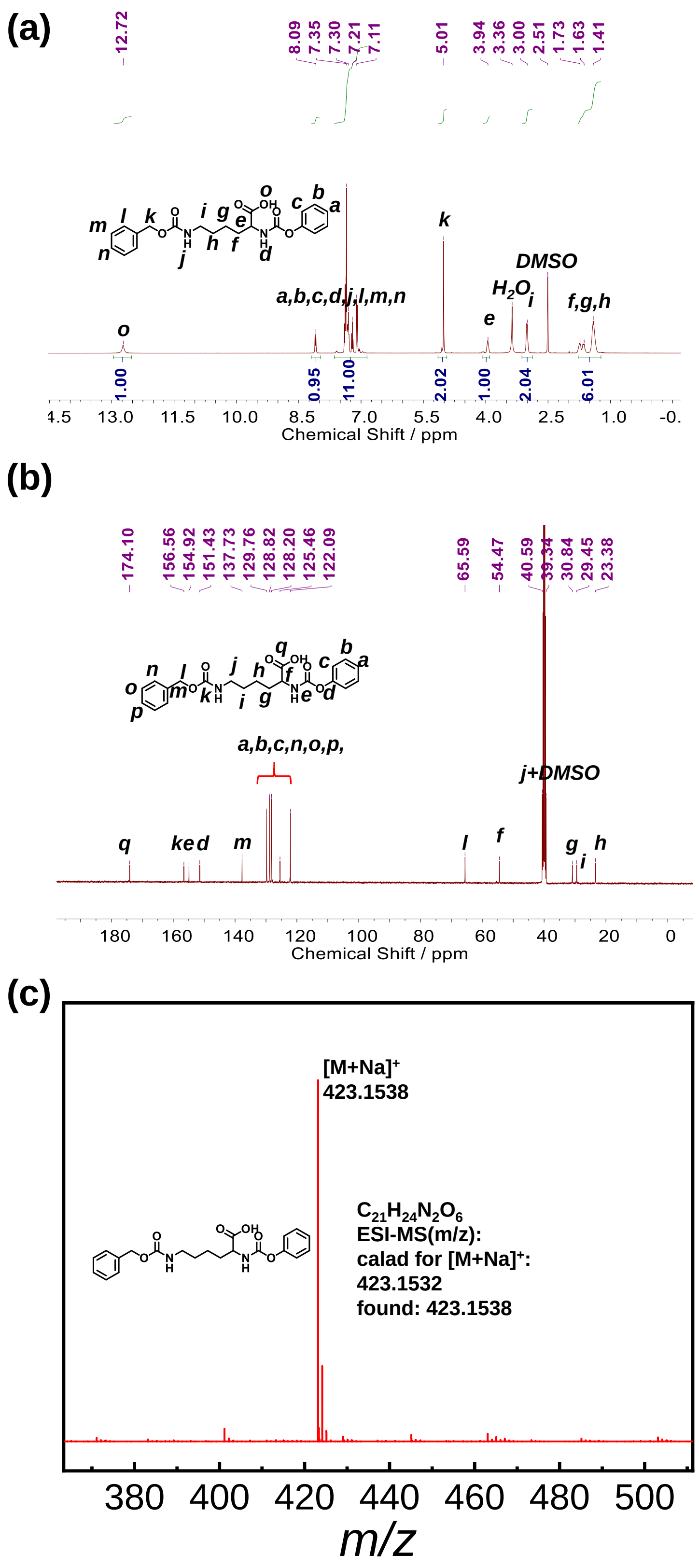

Figure S8. (a) ^{1}H NMR and (b) ^{13}C NMR spectra recorded in DMSO-d_6 for CbzK precursor. (c) ESI-MS spectrum recorded for CbzK precursor.

(a)

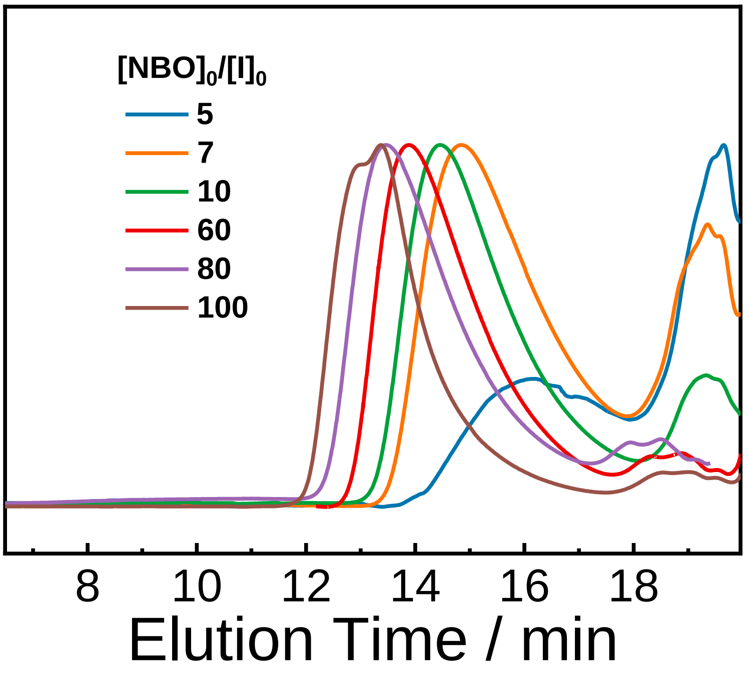

(b)

$[NBO]_0/[I]_0$	$M_{n,GPC}$ (kDa)	$Đ$
5	4.1	1.63
7	6.7	1.89
10	8.2	1.45
60	16.7	1.38
80	22.3	1.35
100	25.3	1.36

Figure S9. (a) GPC elution traces recorded for PNBO synthesized via polymerization of NBO precursor ($[M]_0$ = 0.25 M, DMAc, 60 °C) using *n*-BuNH$_2$ as the initiator. (b) $M_{n,GPC}$ and $Đ$ of as-synthesized PNBO.

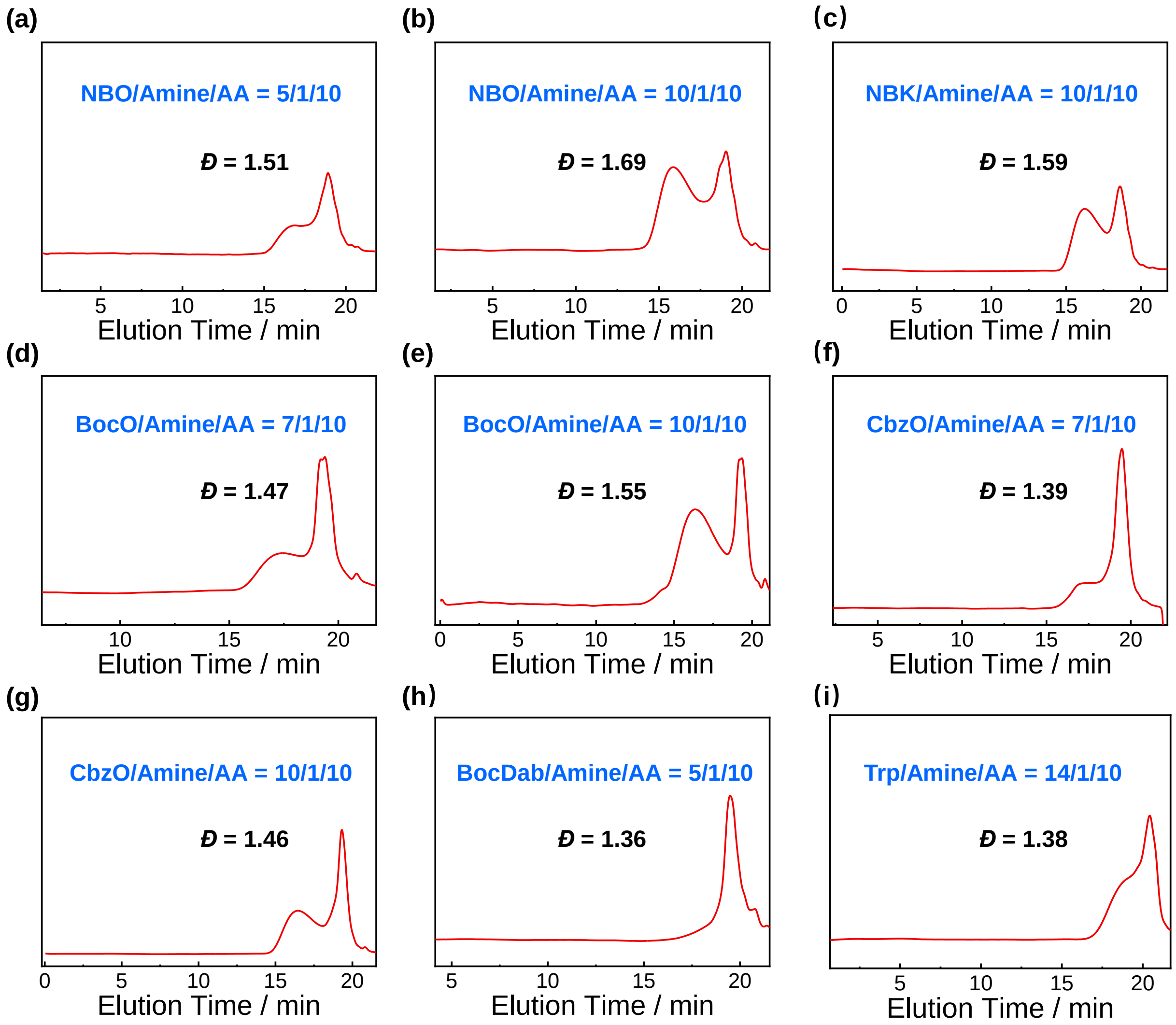

Figure S10. GPC elution traces recorded for polypeptides synthesized via polymerization of a variety of NPCA precursors using *n*-$BuNH_2$ as initiator in the presence of acetic acid ([AA]/[I] = 10/1, $[M]_0$ = 0.25 M, DMAc, 60 °C), revealing bimodal GPC elution traces for polypeptides synthesized at $[M]_0/[I]_0$ feed ratios in the range of 5-14.

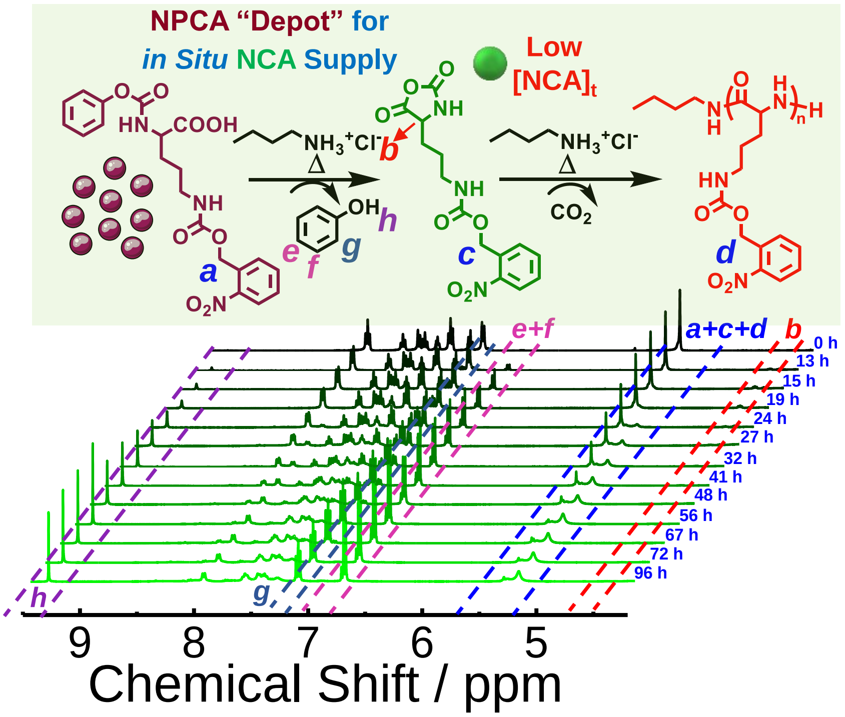

$$\text{Consumed NPCA} = [\text{NCA}]_t + \text{Formed polypeptide}$$

$$\text{Formed polypeptide} = \text{Polymerized NPCA} = [\text{Polypeptide}]_t$$

$$\text{Consumed NPCA} = \frac{[\text{NPCA}]_0 - [\text{NPCA}]_t}{[\text{NPCA}]_0} = \frac{\text{I}(\text{e}+\text{f})/3}{\text{I}(\text{a}+\text{c}+\text{d})/2} = \frac{2\ \ \text{I}(\text{e}+\text{f})}{3\ \ \text{I}(\text{a}+\text{c}+\text{d})}$$

$$\frac{[\text{NCA}]_t}{[\text{NPCA}]_0} = \frac{2\ \ \text{I}(\text{b})}{\text{I}(\text{a}+\text{c}+\text{d})}$$

$$\frac{[\text{NPCA}]_t}{[\text{NPCA}]_0} = 1 - \frac{2\ \ \text{I}(\text{e}+\text{f})}{3\ \ \text{I}(\text{a}+\text{c}+\text{d})}$$

$$[\text{Polypeptide}]_t = \text{Consumed NPCA} - [\text{NCA}]_t$$

$$= \frac{[\text{NPCA}]_0 - [\text{NPCA}]_t}{[\text{NPCA}]_0} - \frac{[\text{NCA}]_t}{[\text{NPCA}]_0}$$

$$= \frac{2\ \ \text{I}(\text{e}+\text{f}) - 6\ \ \text{I}(\text{b})}{3\ \ \text{I}(\text{a}+\text{c}+\text{d})}$$

“I” refers to integration area of given NMR resonance peaks

Figure S11. Schematics illustrating the calculation of polymerization kinetics based on time-dependent ^{1}H NMR data.

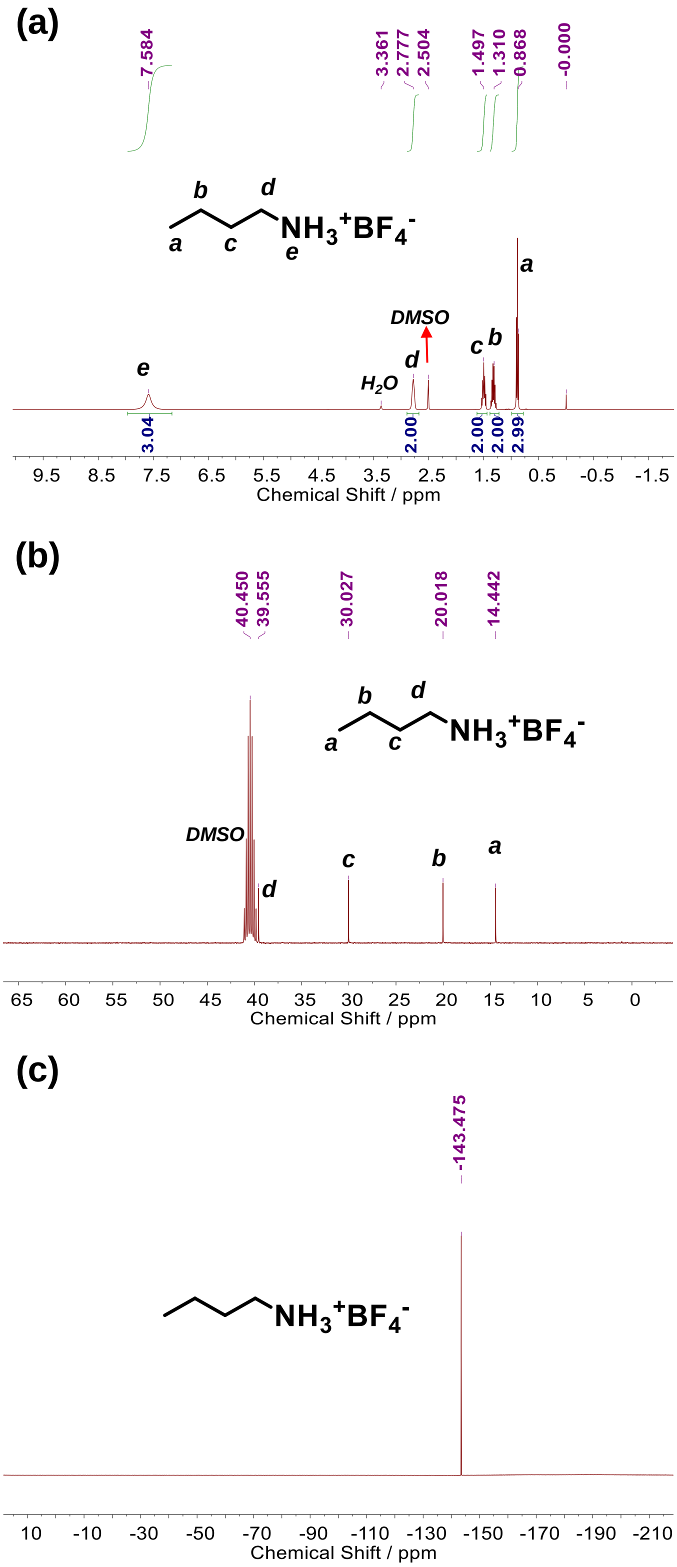

Figure S12. (a) ^{1}H NMR, (b) ^{13}C NMR, and (c) ^{19}F NMR spectra recorded in DMSO-d_6 for *n*-$BuNH_3^+BF_4^-$.

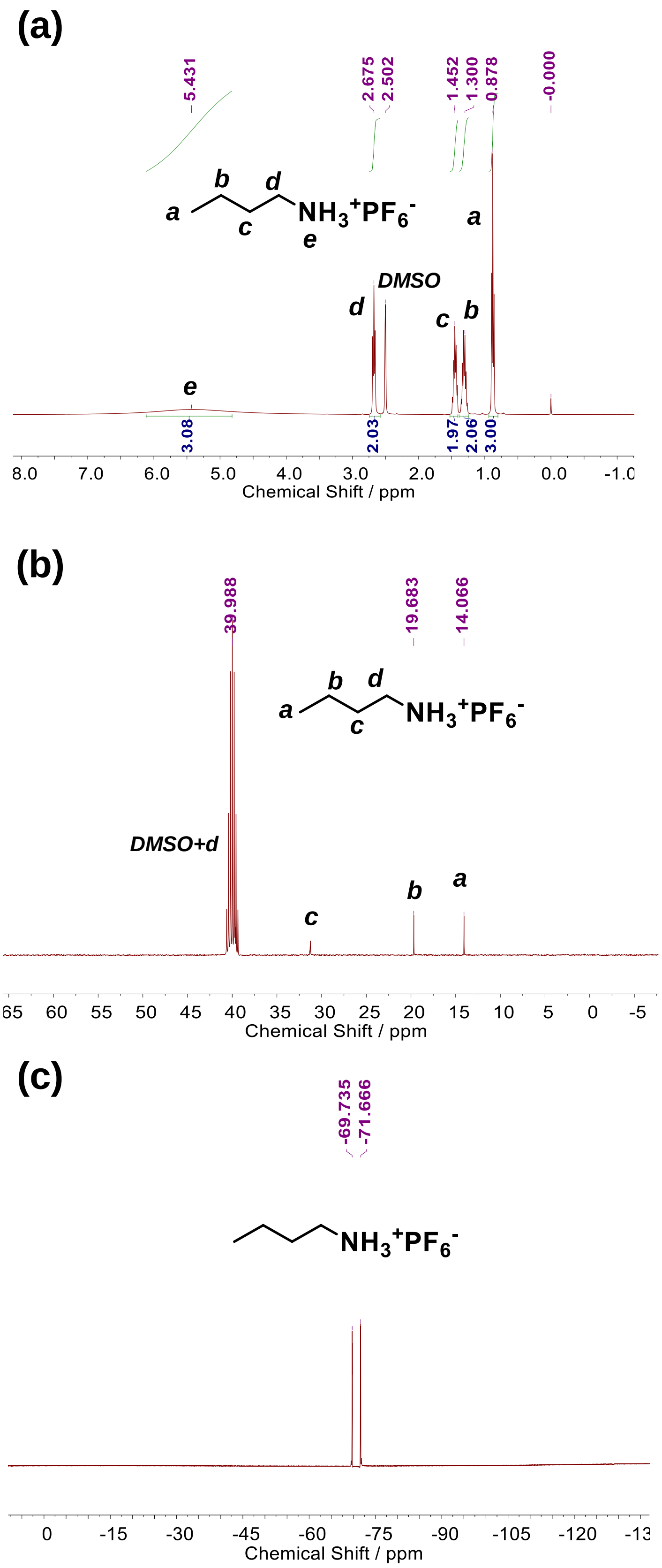

Figure S13. (a) ^{1}H NMR, (b) ^{13}C NMR, and (c) ^{19}F NMR spectra recorded in DMSO-d_6 for *n*-$BuNH_3^{+}PF_6^{-}$.

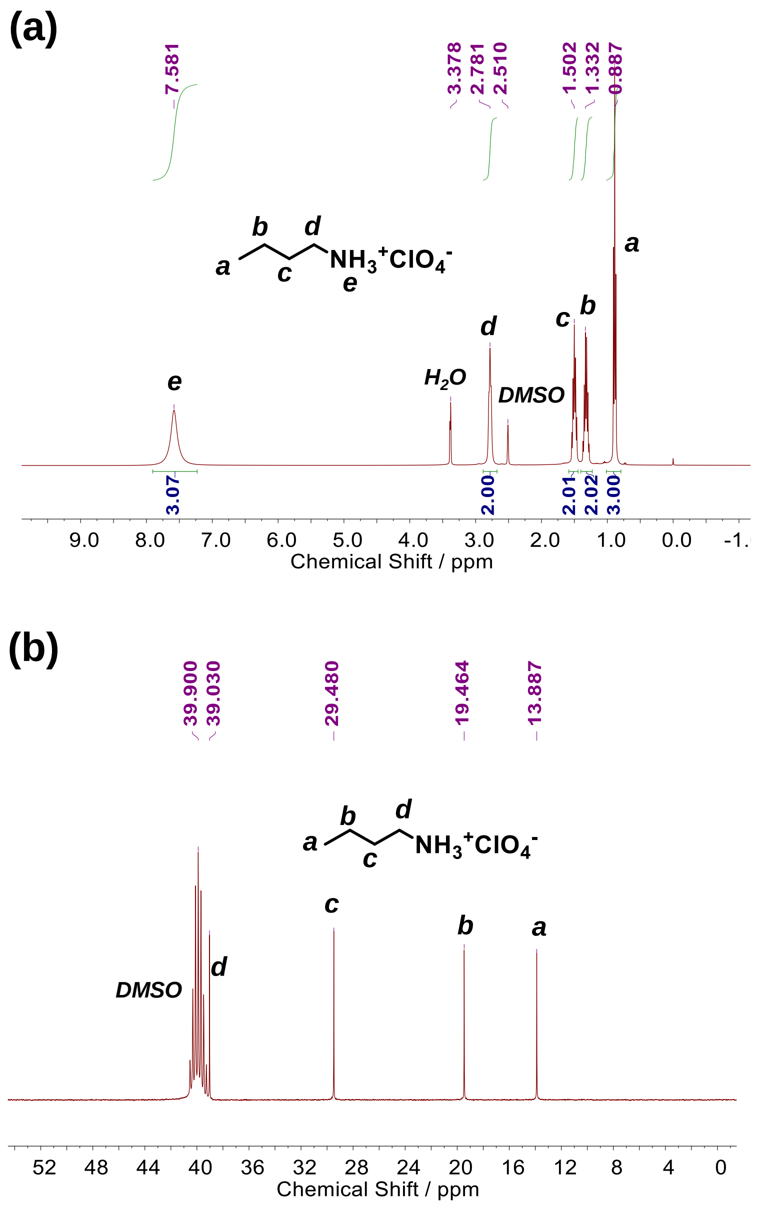

Figure S14. (a) ^{1}H NMR and (b) ^{13}C NMR spectra recorded in DMSO-d_6 for *n*-$BuNH_3^+ClO_4^-$.

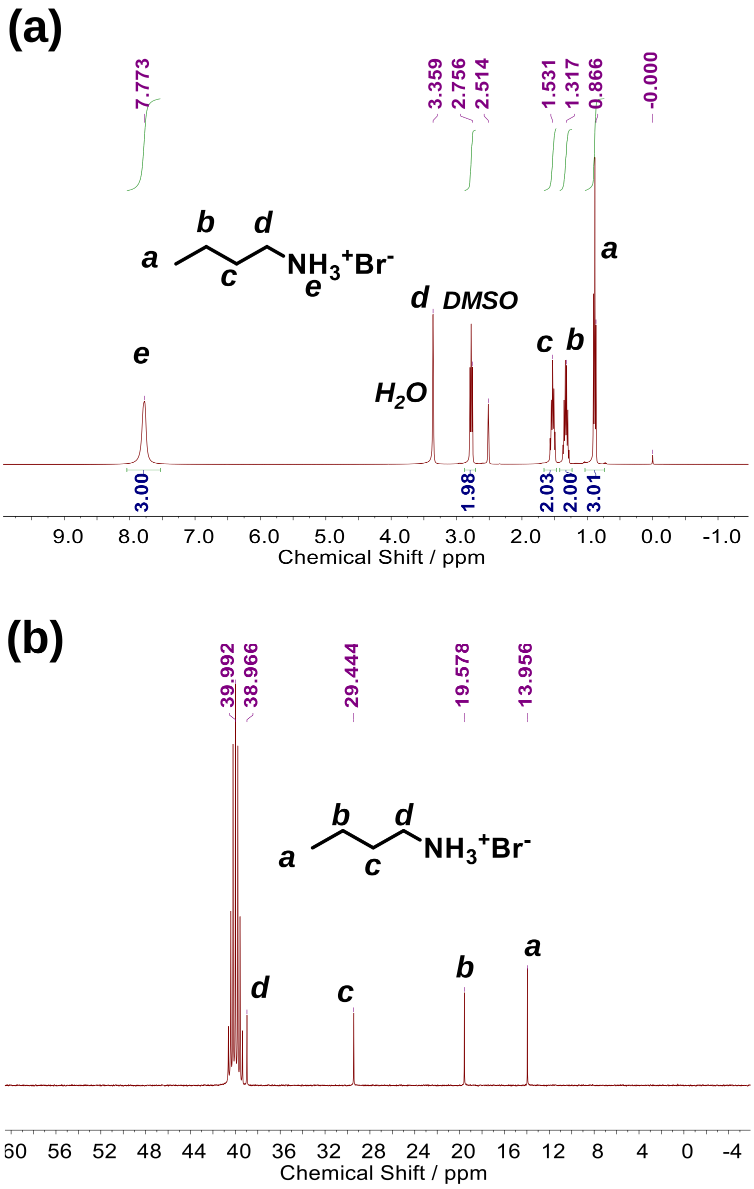

Figure S15. (a) ^{1}H NMR and (b) ^{13}C NMR spectra recorded in DMSO-d_6 for *n*-$BuNH_3^+Br^-$.

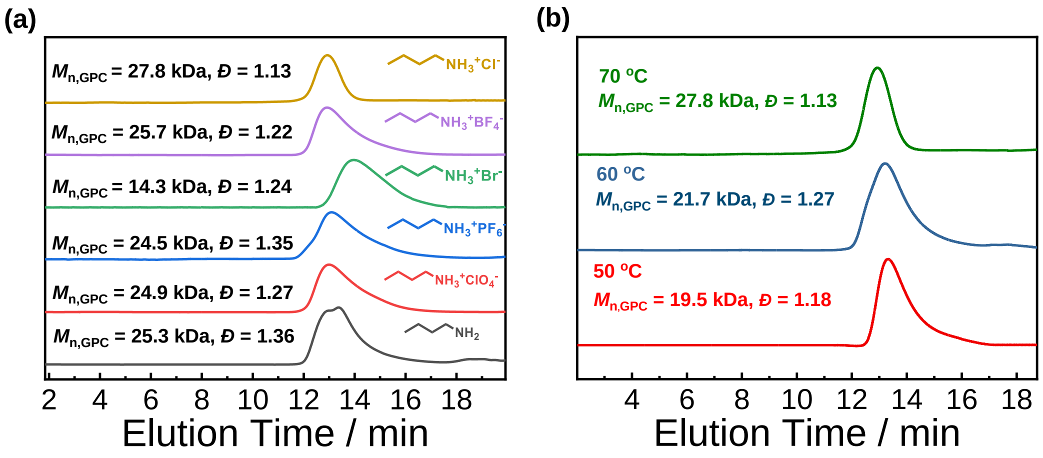

Figure S16. Evolution of GPC elution traces recorded for NPCA polymerizations under various conditions. (a) GPC elution traces of PNBO polypeptides synthesized using *n*-$BuNH_3^+X^-$ with varying counter ions (X^- refers to Cl^-, BF_4^-, Br^-, PF_6^-, and ClO_4^-) and *n*-$BuNH_2$ as initiator ($[M]_0/[I]_0$ = 100). (b) GPC elution traces of PNBO synthesized using *n*-$BuNH_3^+Cl^-$ initiator at varying temperatures (50 ºC, 60 ºC, and 70 ºC; $[M]_0/[I]_0$ = 100). All polymerizations were conducted at $[M]_0$ = 0.25 M in DMAc and 70 ºC, unless otherwise noted.

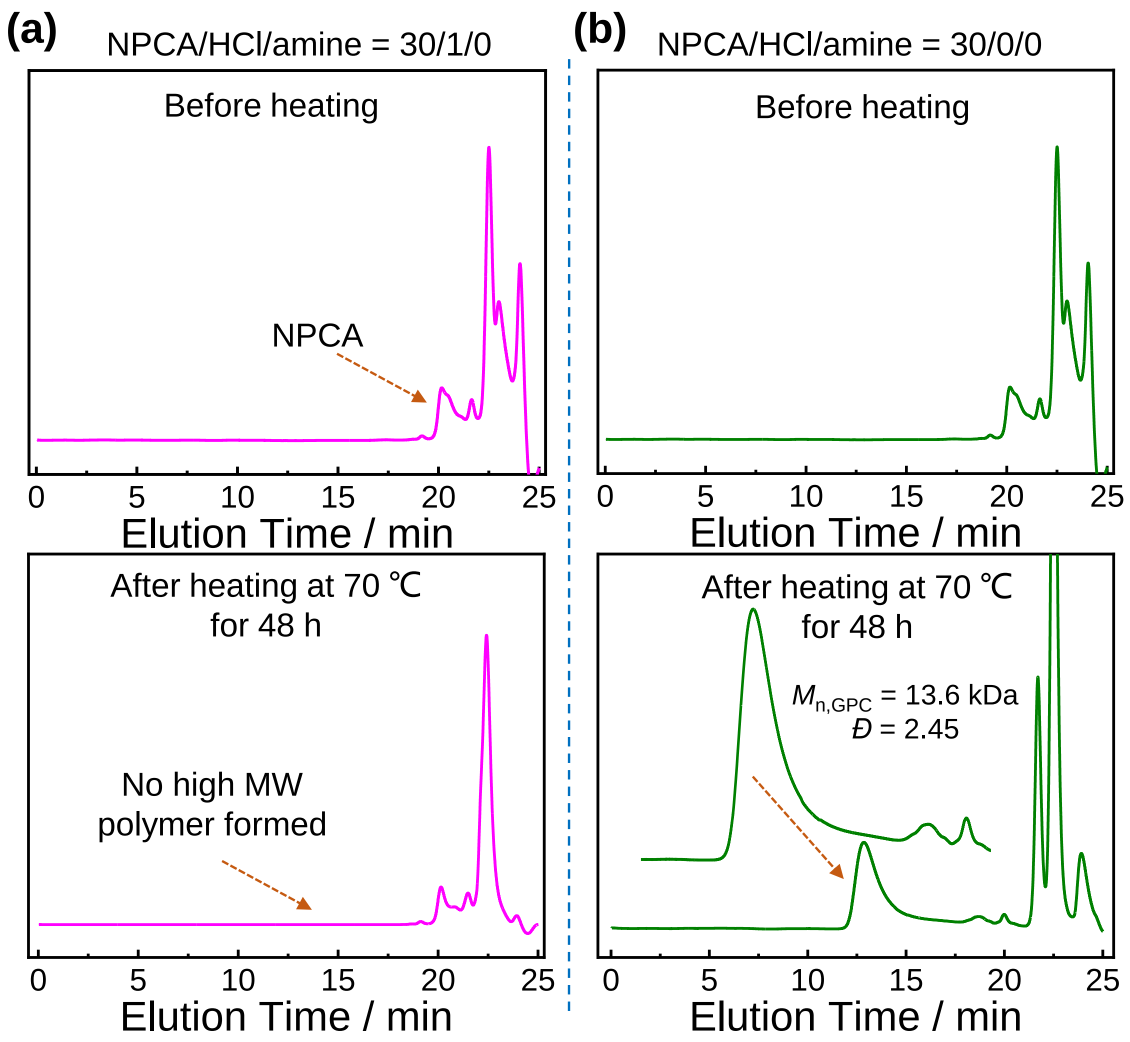

Figure S17. Comparison of CbzK polymerizations in DMAc (0.25 M) (a) with (b) without HCl, which was introduced as hydrogen chloride solution in 1,4-dioxane (4.0 M). No primary ammonium or amine initiator was added.

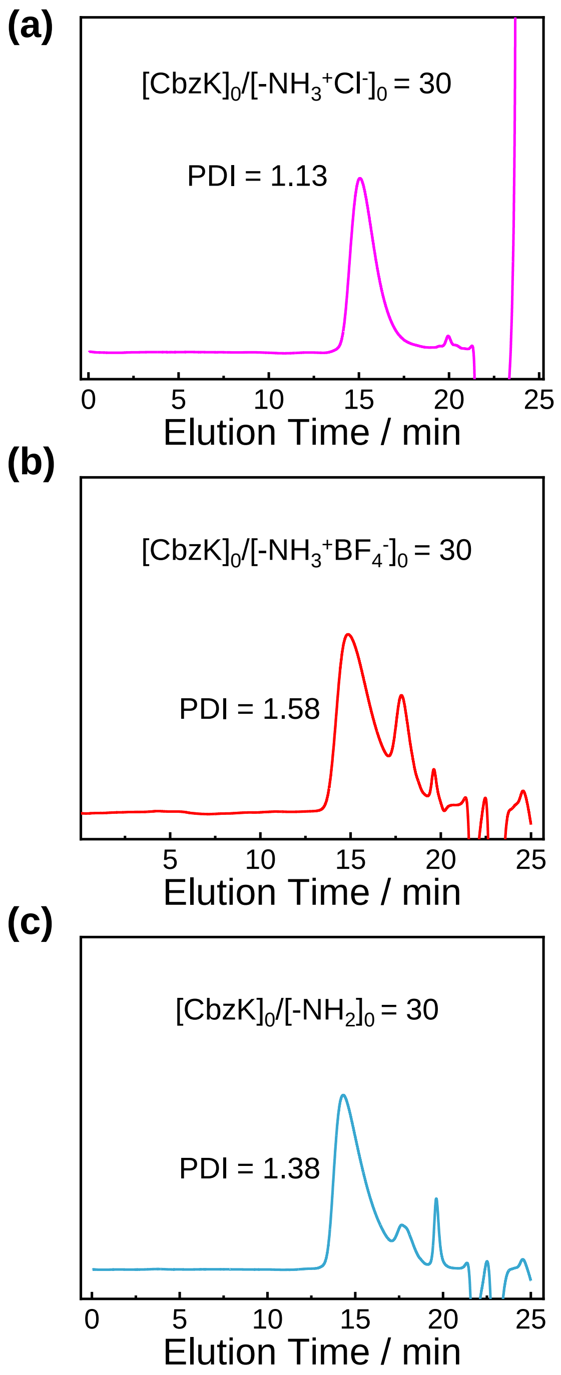

Figure S18. GPC elution traces recorded for CbzK polymerizations using (a) *n*-$BuNH_3^+Cl^-$, (b) *n*-$BuNH_3^+BF_4^-$, and (c) *n*-$BuNH_2$ as initiators at $[M]_0/[I]_0$ = 30. All polymerizations were conducted at $[M]_0$ = 0.25 M in DMAc.

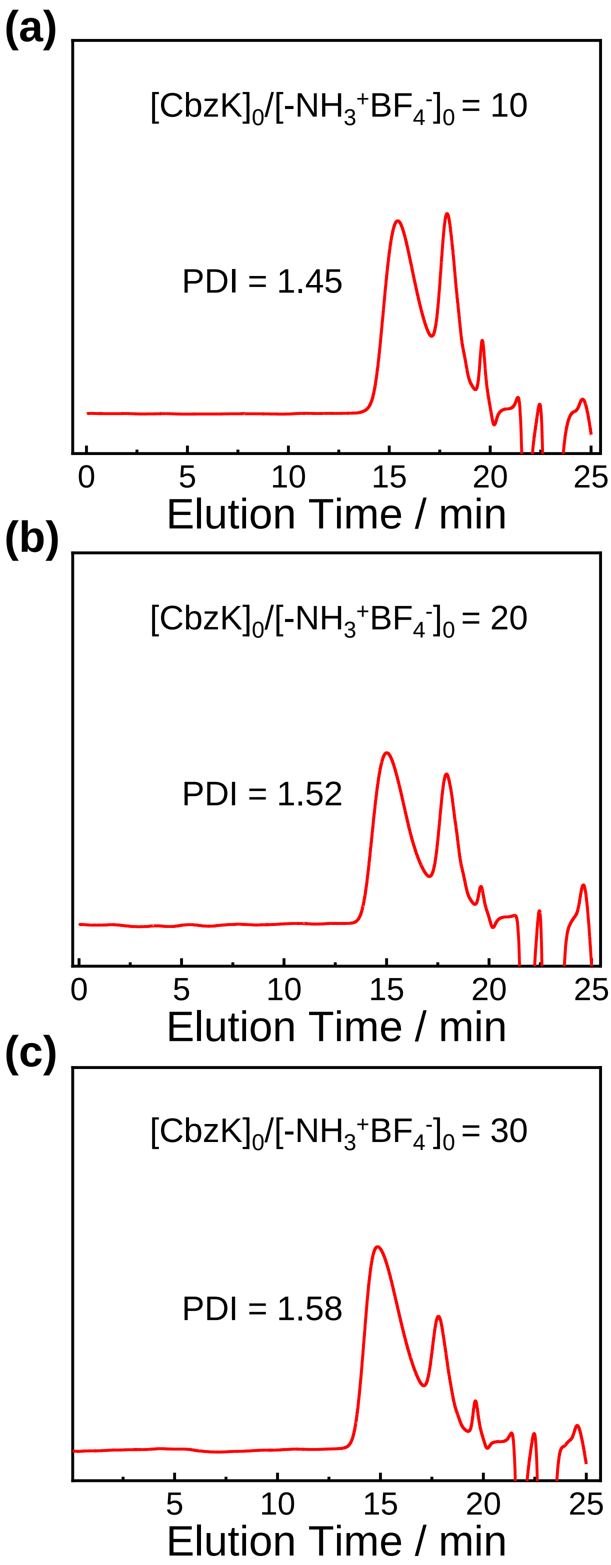

Figure S19. GPC elution traces recorded for CbzK polymerization using *n*-$BuNH_3^+BF_4^-$ as initiator at varying $[M]_0/[I]_0$ ratios (10, 20, and 30). All polymerizations were conducted in DMAc at $[M]_0$ = 0.25 M and 70 °C.

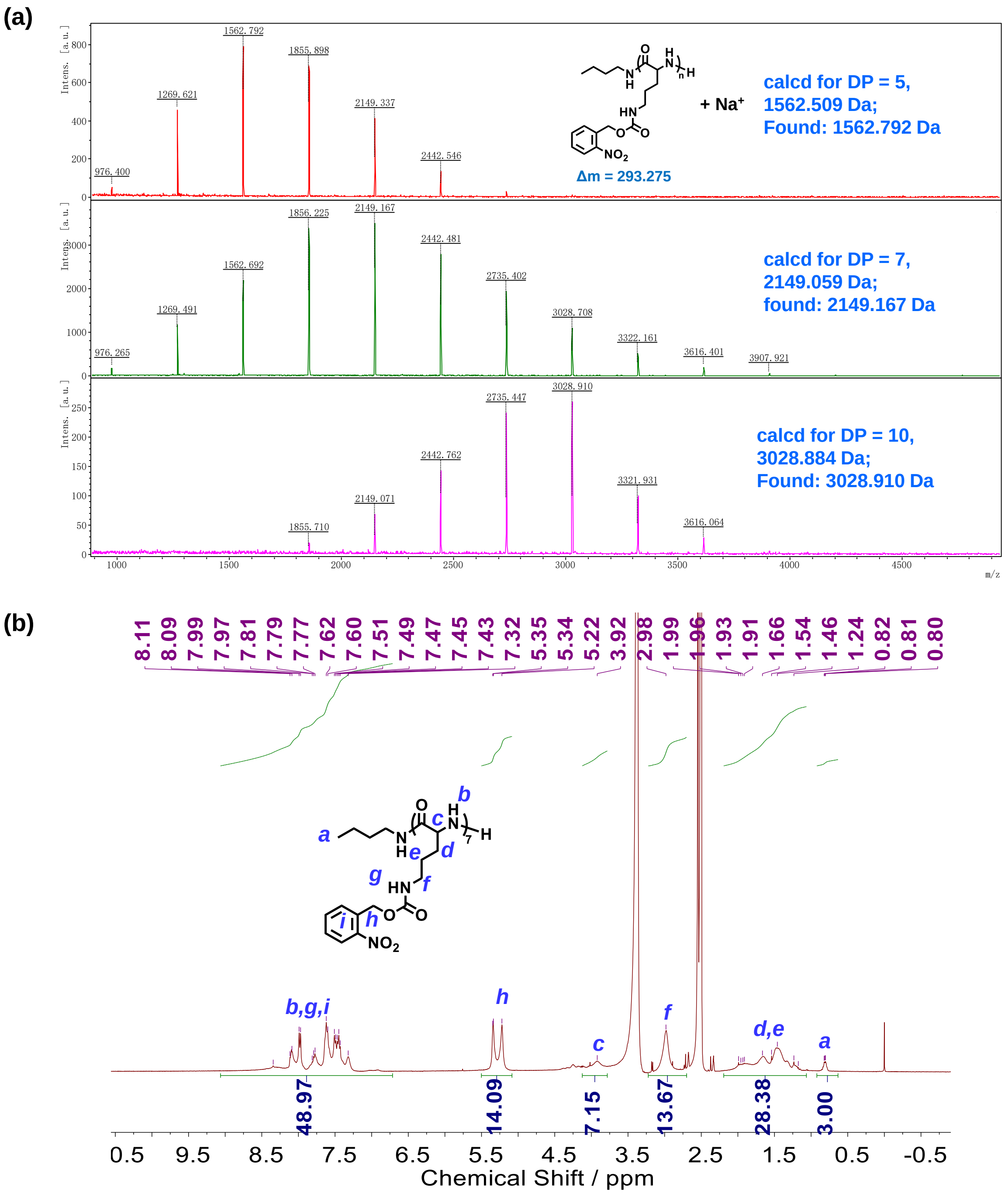

Figure S20. (a) MALDI-TOF MS spectra recorded for PNBO polypeptides synthesized via polymerization of NBO precursor at $[M]_0/[I]_0$ ratios of 5, 7, and 10. ($[M]_0$ = 0.25 M, DMAc, 70 ^{o}C) using n-$BuNH_3^+Cl^-$ as the initiator. (b) Corresponding ^{1}H NMR spectrum recorded $PNBO_7$ in DMSO-d_6.

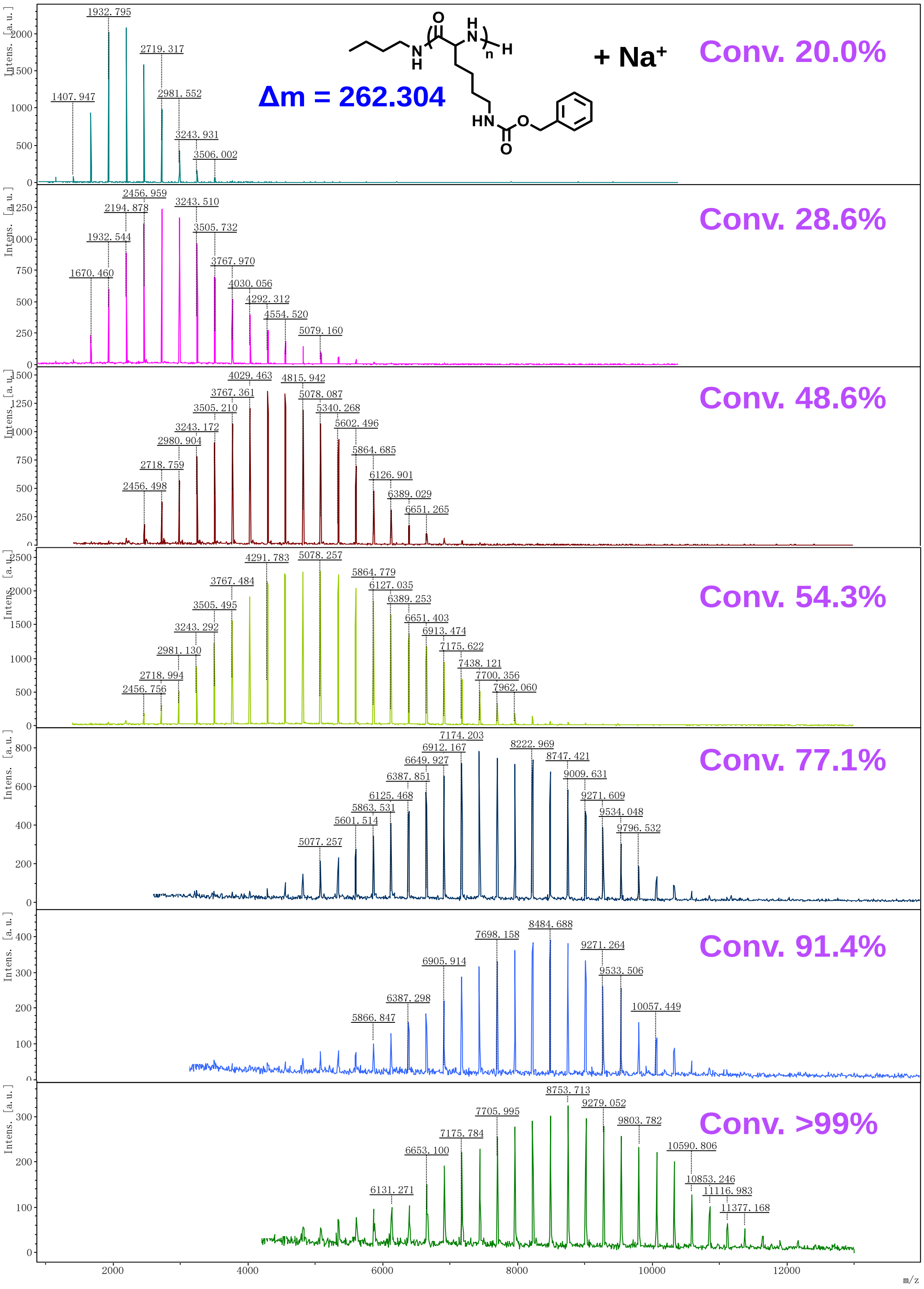

Figure S21. Evolution of MALDI-TOF MS spectra with polypeptide conversions recorded for the synthesis of PCbzK in a glove box ($[M]_0/[I]_0 = 35$, $[M]_0 = 0.25$ M, DMAc, 70 °C).

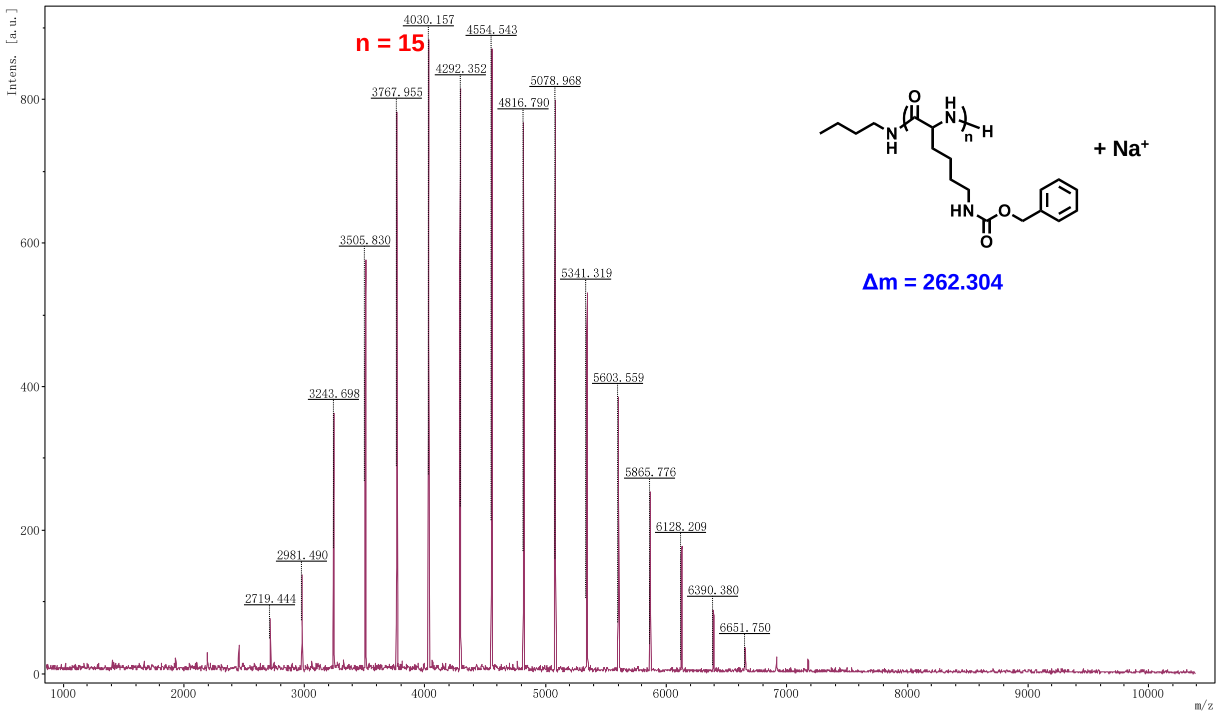

Figure S22. MALDI-TOF MS spectrum recorded for PCbzK$_{15}$ synthesized using *n*-BuNH$_3^+$Cl$^-$ as the initiator ($[M]_0$ = 0.25 M, DMAc, 70 ^oC).

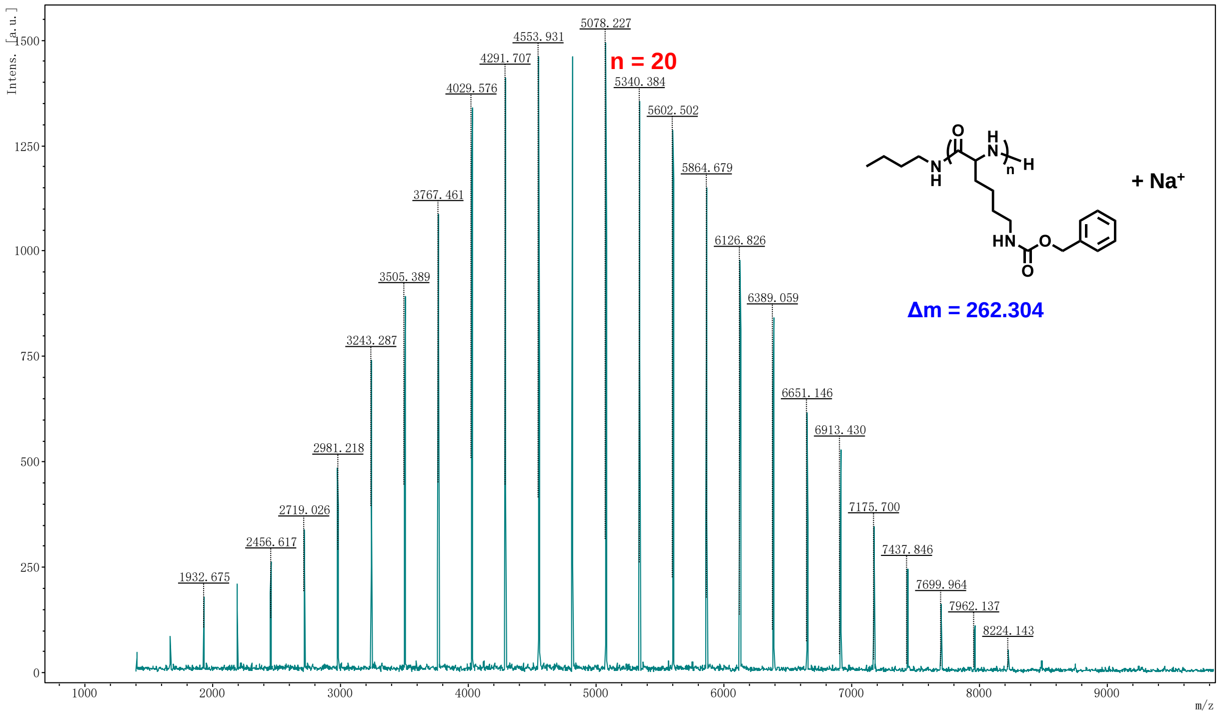

Figure S23. MALDI-TOF MS spectrum recorded for $PCbzK_{20}$ synthesized using *n*-$BuNH_3^+Cl^-$ as the initiator ($[M]_0$ = 0.25 M, DMAc, 70 °C).

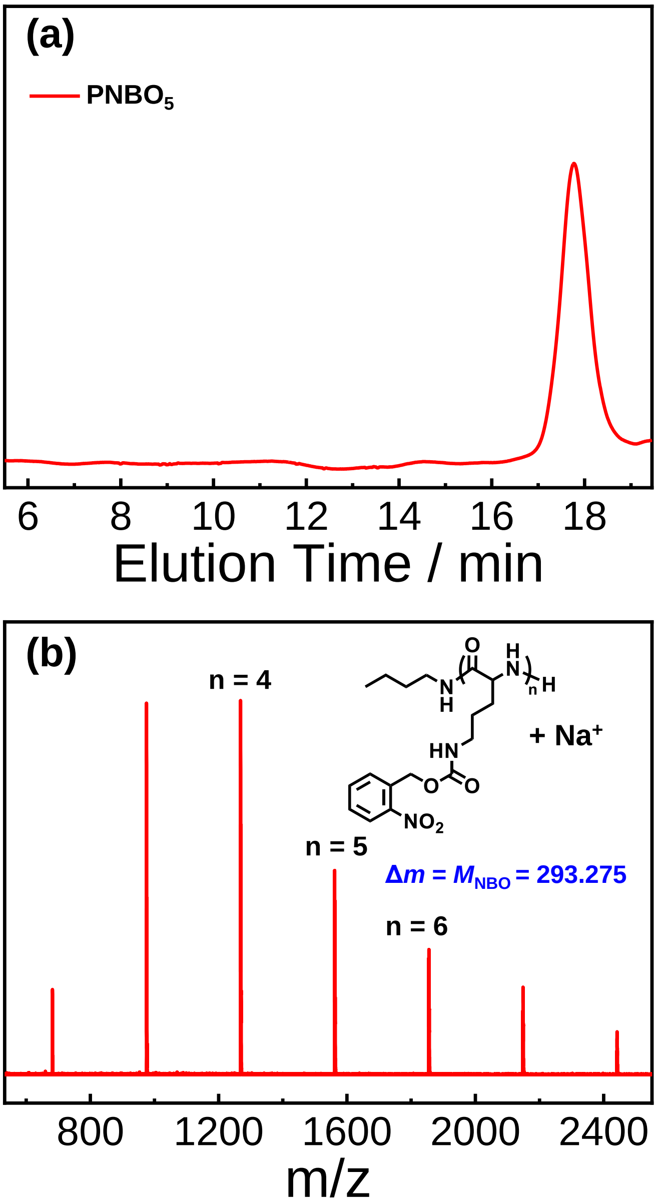

Figure S24. (a) GPC elution trace and (b) MALDI-TOF MS spectrum recorded for $PNBO_5$ synthesized via polymerization of NBO precursor ($[M]_0$ = 0.25 M, DMAc, 70 ^oC) using *n*-$BuNH_3^+Cl^-$ as the initiator.

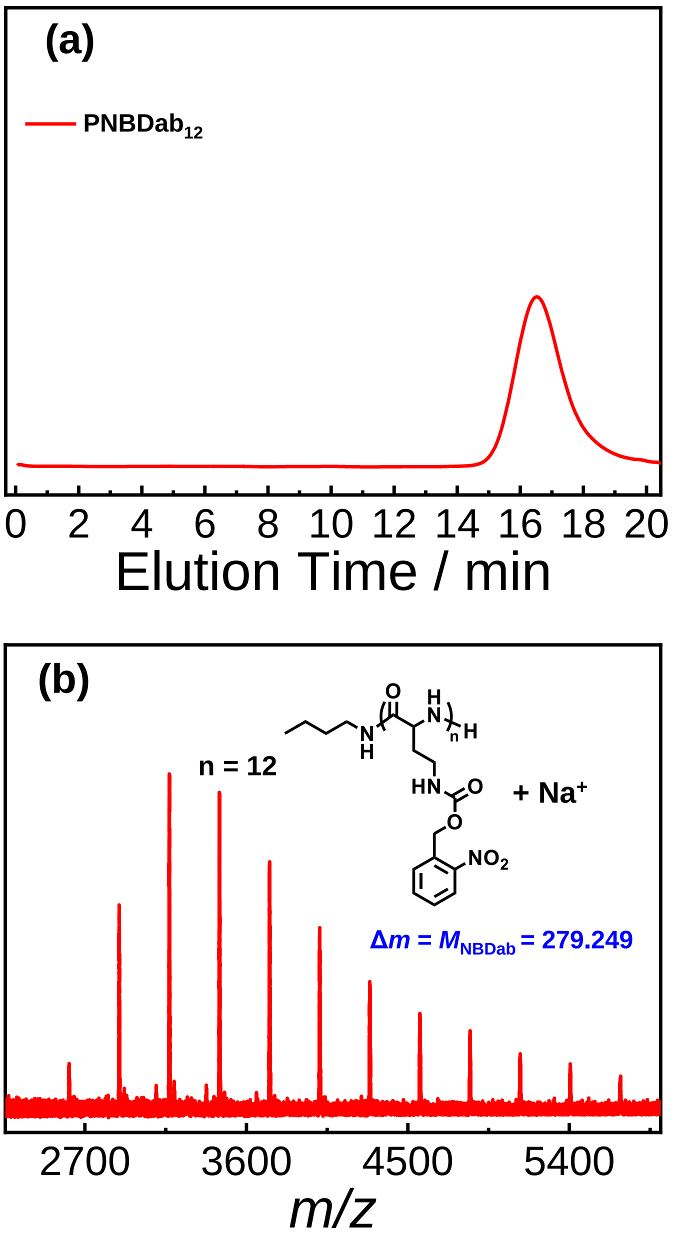

Figure S25. (a) GPC elution trace and (b) MALDI-TOF MS spectrum recorded for $PNBDab_{12}$ synthesized via polymerization of NBDab precursor ($[M]_0$ = 0.25 M, DMAc, 70 °C) using *n*-$BuNH_3^+Cl^-$ as the initiator.

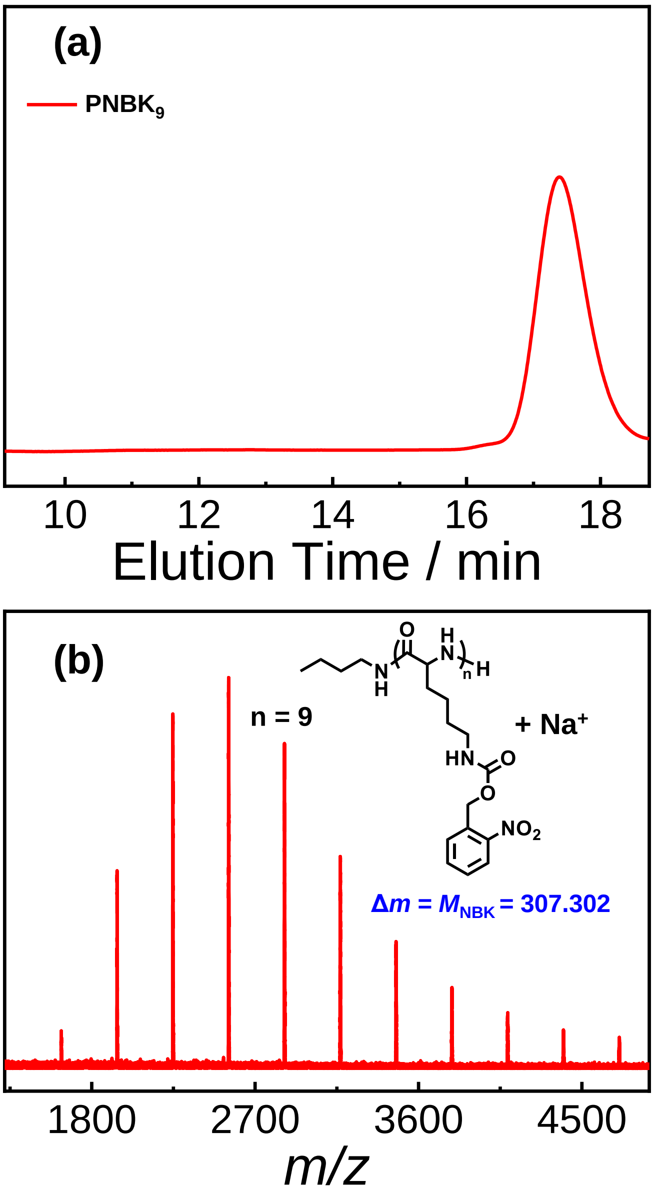

Figure S26. (a) GPC elution trace and (b) MALDI-TOF MS spectrum recorded for $PNBK_9$ synthesized via polymerization of NBK precursor ($[M]_0$ = 0.25 M, DMAc, 70 °C) using *n*-$BuNH_3^+Cl^-$ as the initiator.

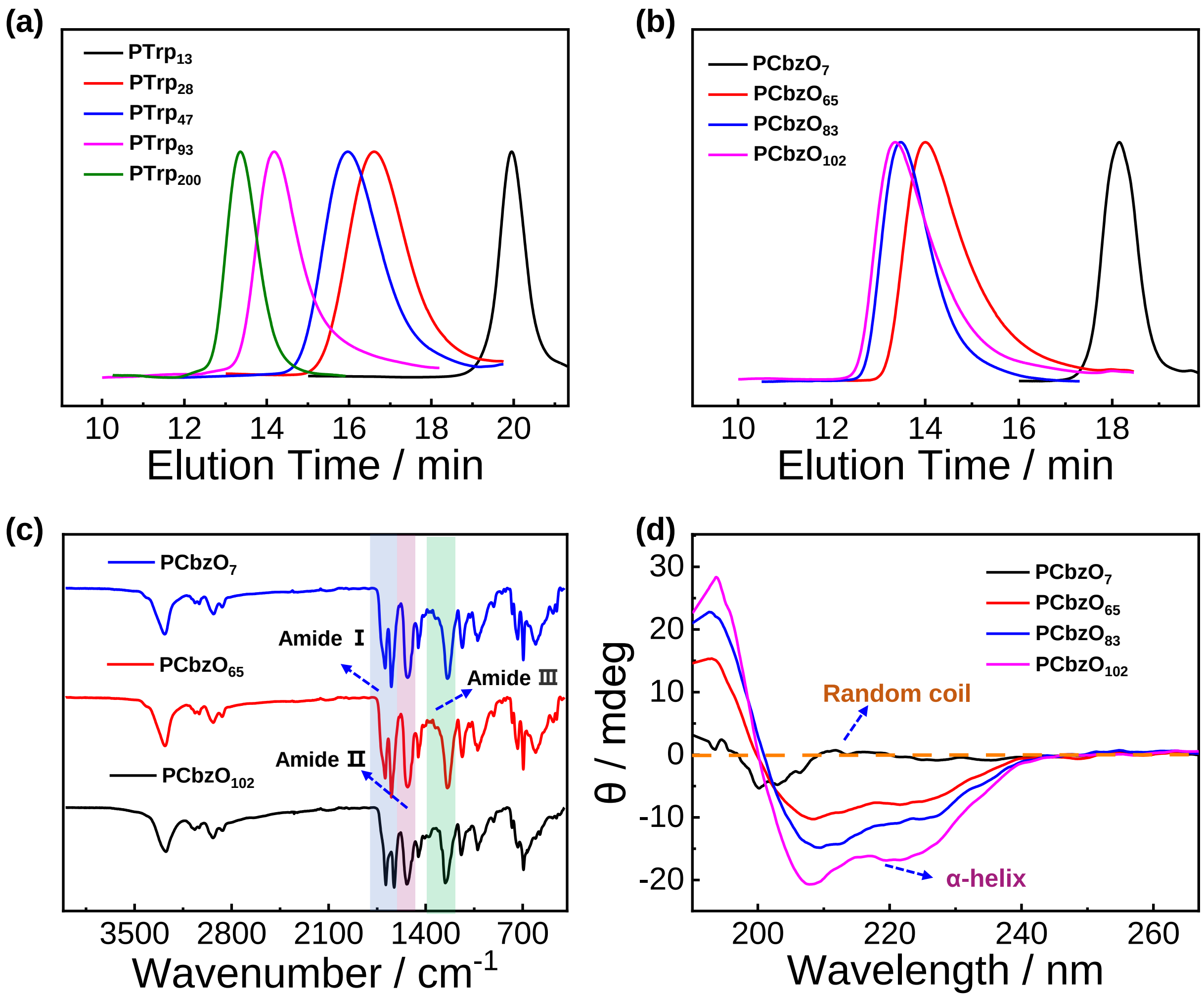

Figure S27. (a) GPC elution traces of PTrp polypeptides synthesized at varying $[M]_0/[I]_0$ feed ratios using *n*-$BuNH_3^+Cl^-$ as initiator. (b) GPC elution traces of PCbzO polypeptides synthesized at varying $[M]_0/[I]_0$ feed ratios using *n*-$BuNH_3^+Cl^-$ as the initiator. (c) ATR-FTIR spectra recorded for $PCbzO_7$, $PCbzO_{65}$, and $PCbzO_{102}$. (d) Circular dichroism (CD) spectra recorded for $PCbzO_7$, $PCbzO_{65}$, $PCbzO_{83}$, and $PCbzO_{102}$ in HFIP at 20 °C (0.05 mg/mL). All polymerizations were conducted at $[M]_0$ = 0.25 M in DMAc and 70 °C.

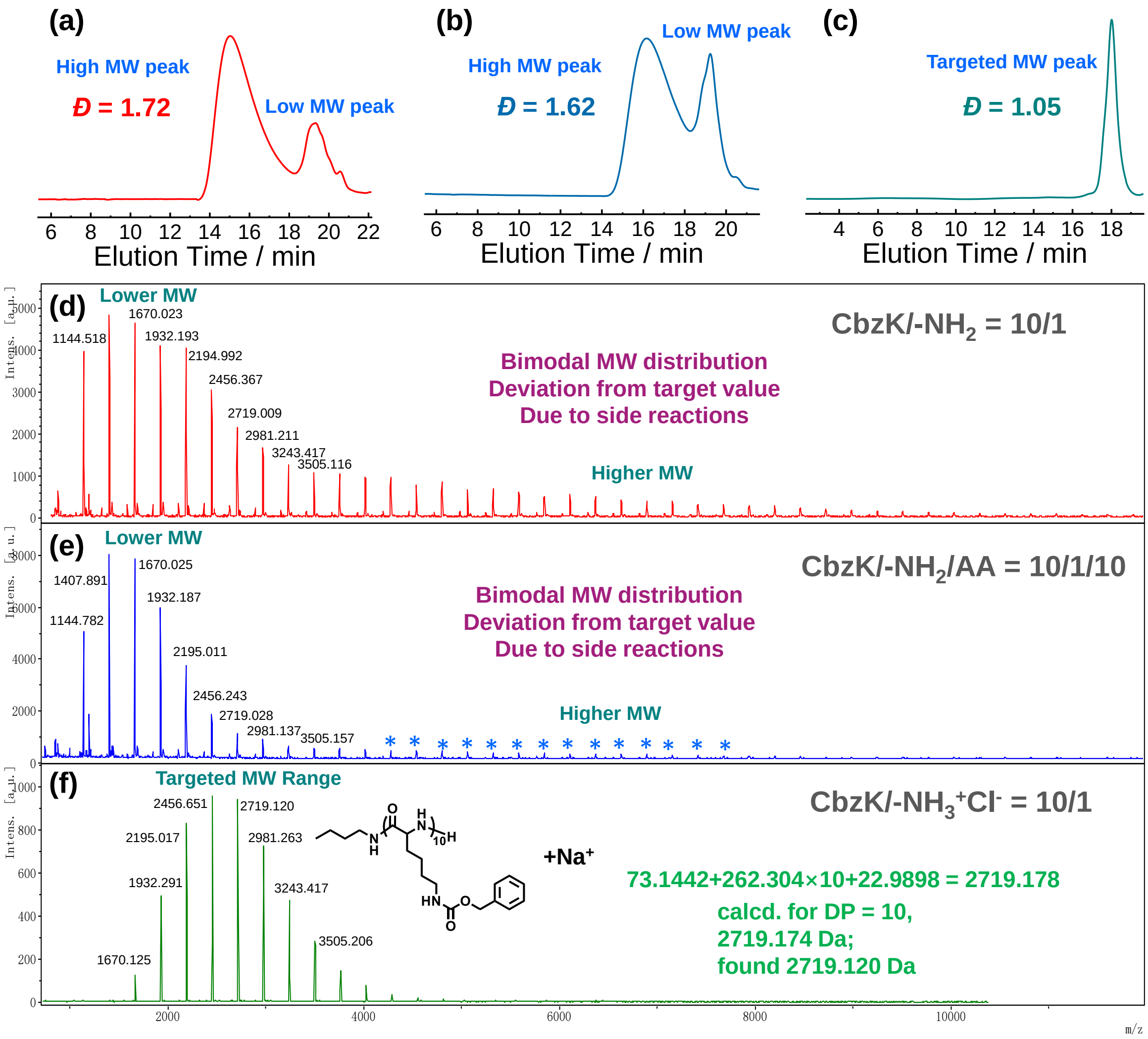

Figure S28. GPC elution traces and MALDI-TOF MS spectra recorded for PCbzK synthesized using varying initiators: (a, d) *n*-BuNH$_2$ ($[M]_0/[I]_0$ = 10/1), (b, e) *n*-BuNH$_2$ in combination with acetic acid ($[M]_0/[AA]/[I]_0$ = 10/10/1), and (c, f) *n*-BuNH$_3^+$Cl$^-$ ($[M]_0/[I]_0$ = 10/1).

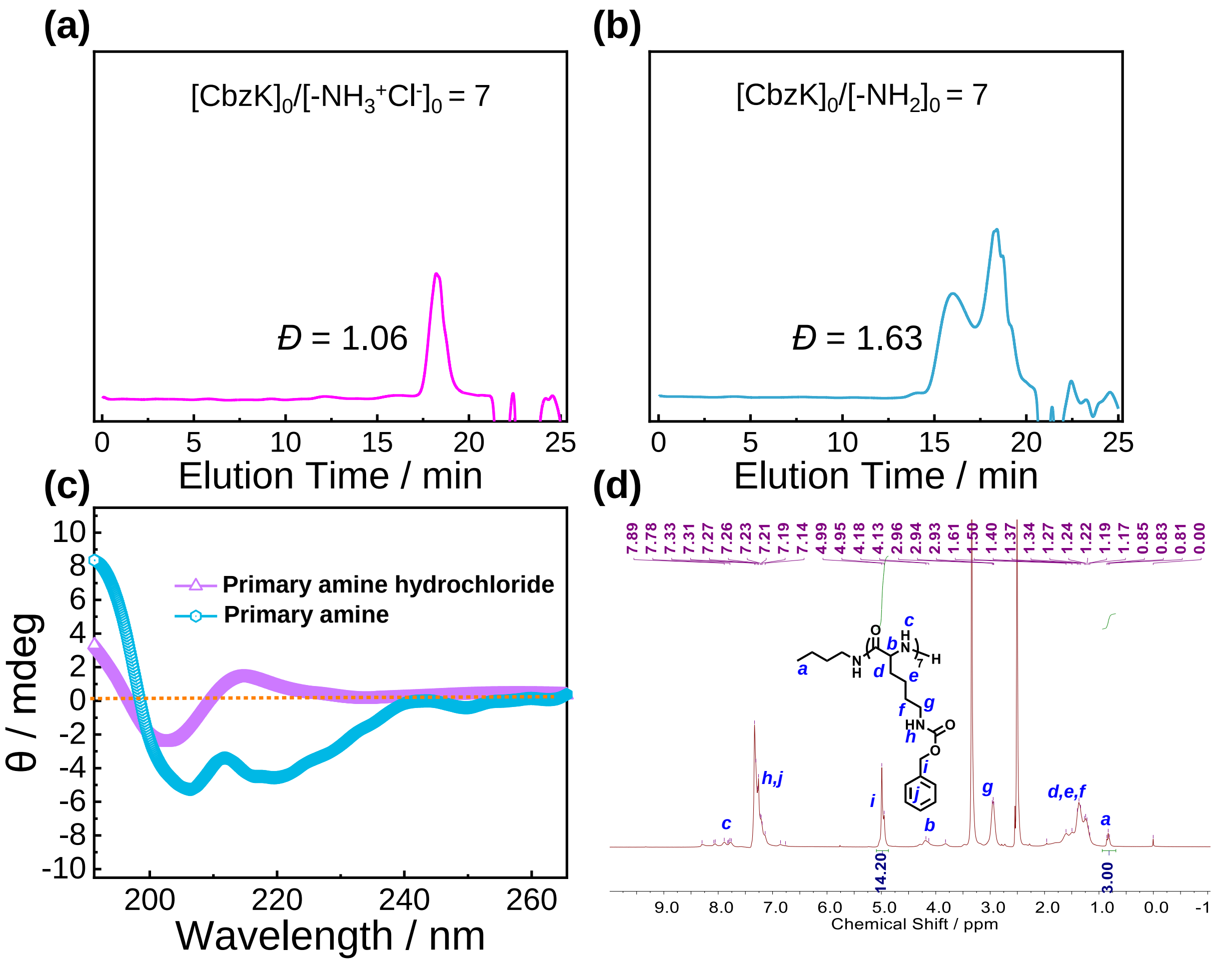

Figure S29. (a, b) GPC elution traces and (c) CD spectra recorded for NPCA polymerization products ($[M]_0/[I]_0$ = 7/1) using (a) *n*-$BuNH_3^+Cl^-$ and (b) *n*-$BuNH_2$ as initiator. (c) ^1H NMR spectrum recorded in DMSO-d_6 for $PCbzK_7$ synthesized via polymerization of CbzK precursor ($[M]_0$ = 0.25 M, DMAc) using *n*-$BuNH_2$ initiator.

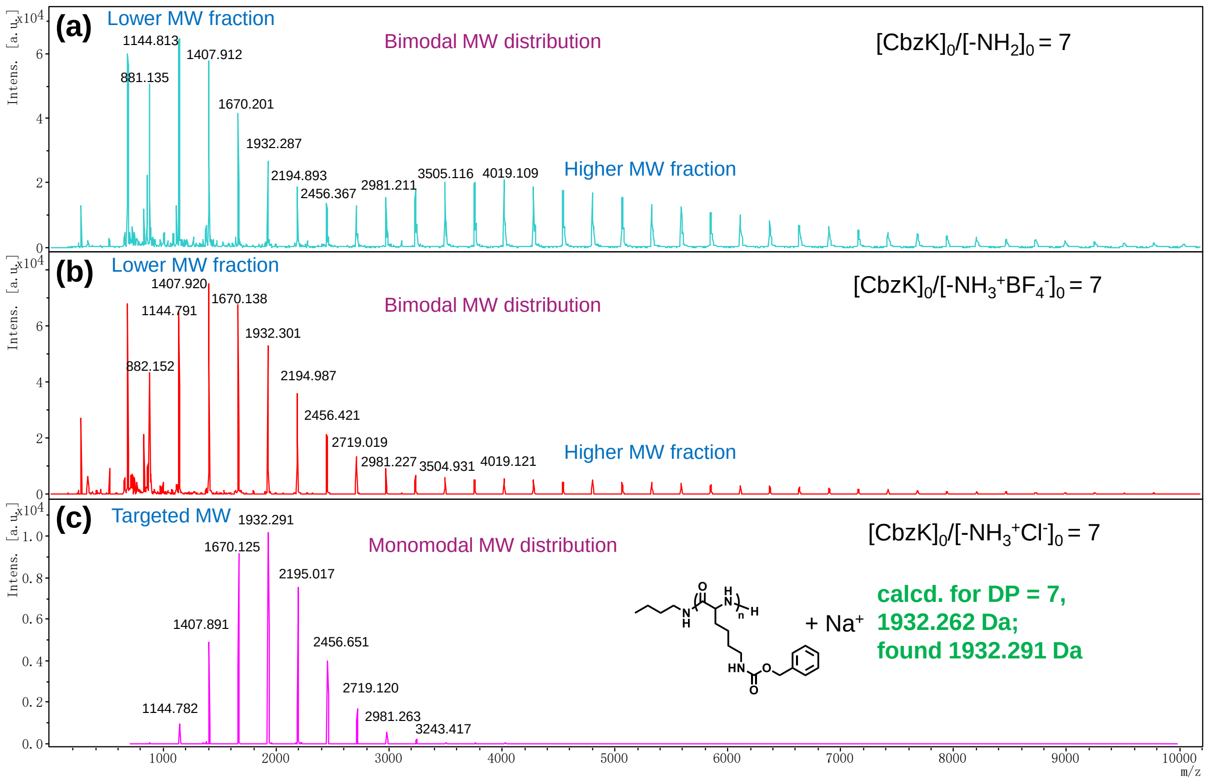

Figure S30. MALDI-TOF MS recorded for PCbzK polypeptides synthesized at $[M]_0/[I]_0 = 7$ using (a) *n*-$BuNH_2$, (b) *n*-$BuNH_3^+BF_4^-$, and (c) *n*-$BuNH_3^+Cl^-$ as initiator.

(a)

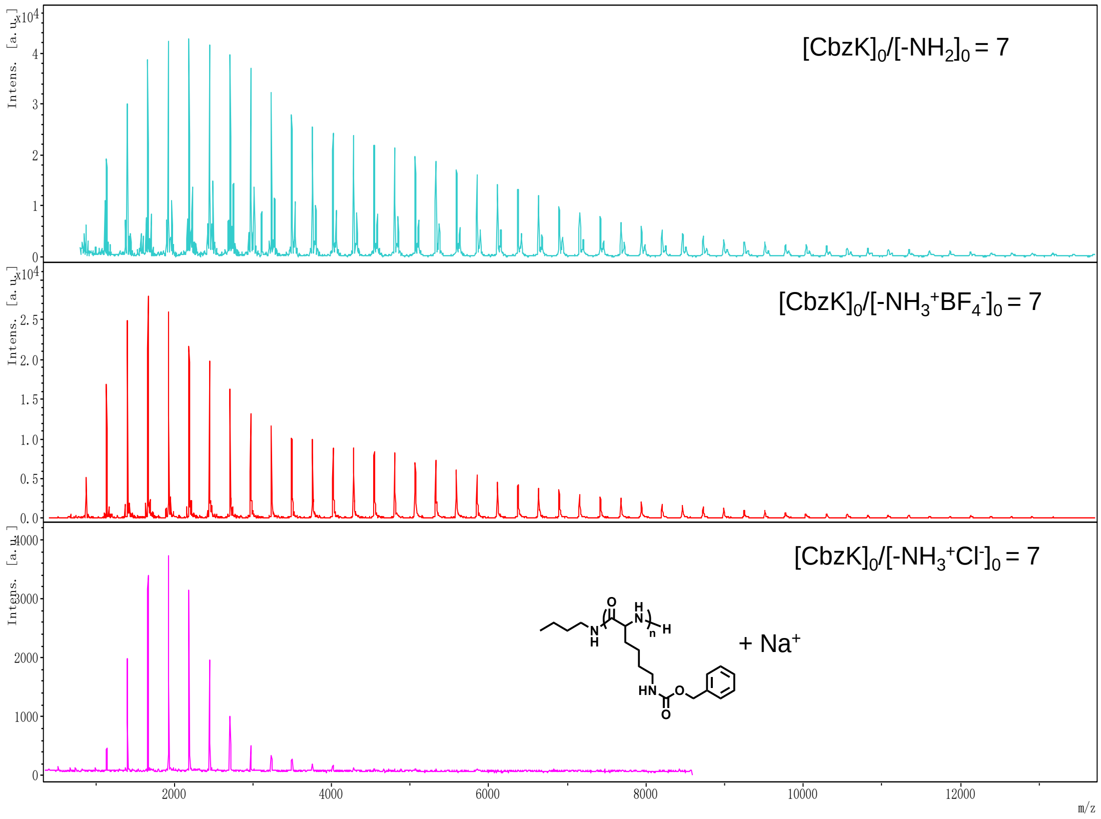

(b)

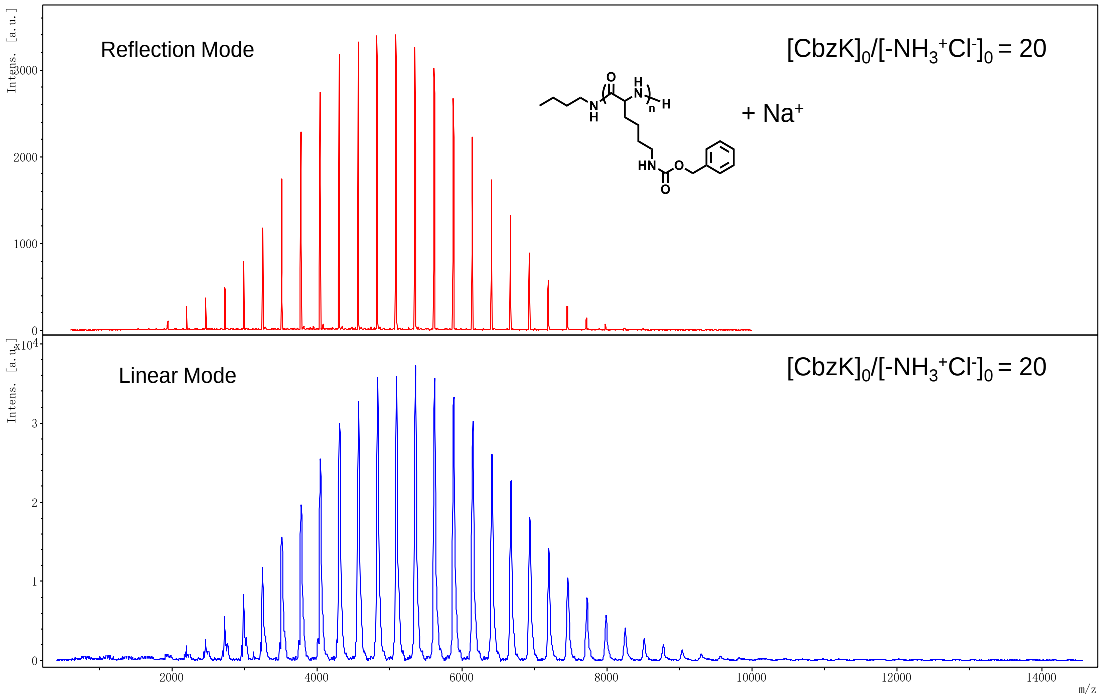

Figure S31. MALDI-TOF MS data recorded for PCbzK polypeptides synthesized at [CbzK]/[I] ratios of (a) 7 and (b) 20 using *n*-BuNH$_2$, *n*-BuNH$_3$$^+BF_4$$^-$, and *n*-BuNH$_3$$^+Cl^-$ as initiators, respectively. All MALDI spectra were recorded in positive linear mode unless otherwise noted.

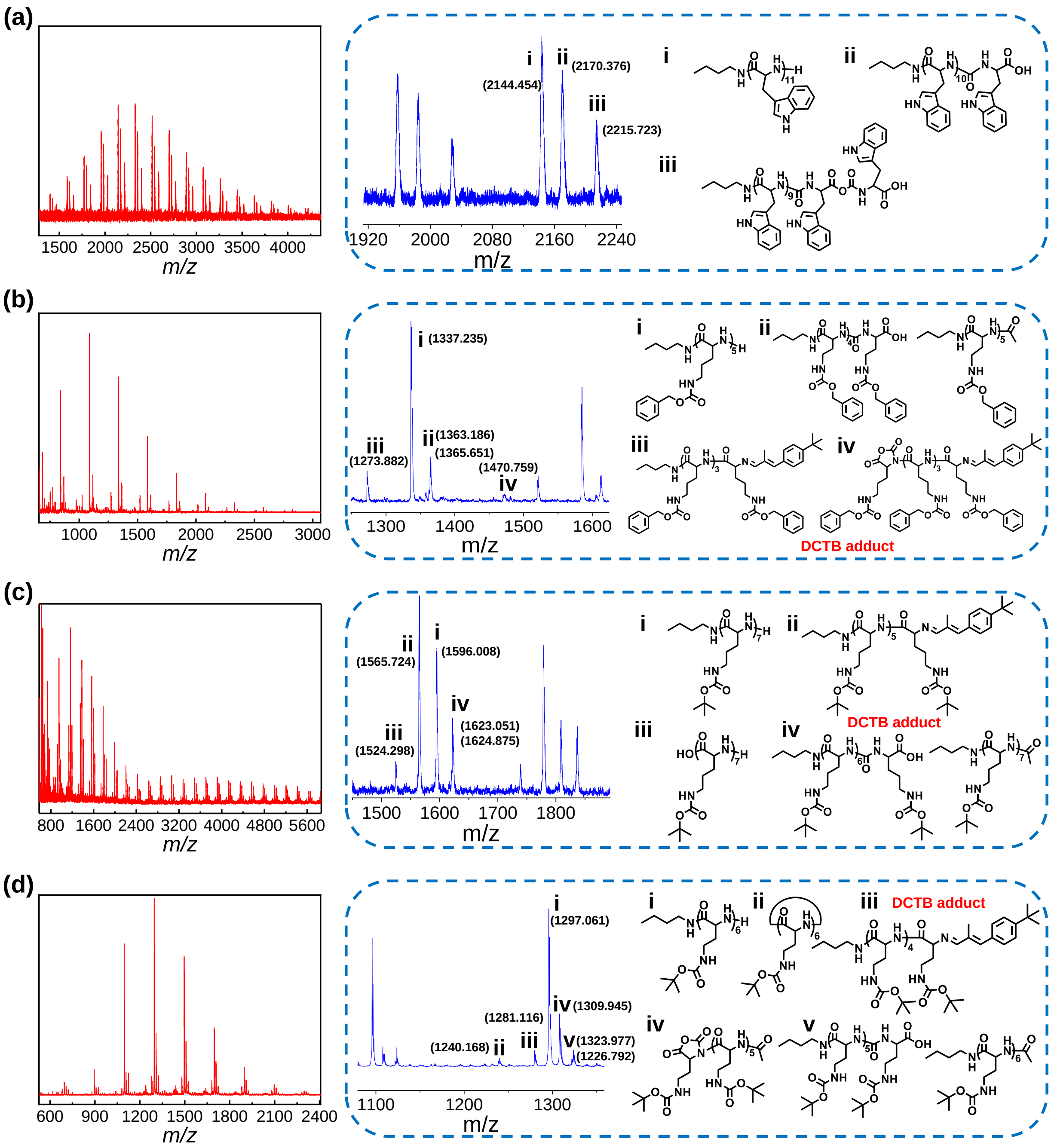

Figure S32. MALDI-TOF MS spectra recorded for (a) PTrp, (b) PCbzO, (c) PBocO, and (d) PBocDab polypeptides synthesized by using *n*-BuNH$_2$ as the initiator, exhibiting prominent impurity peaks corresponding to side reactions occurred during polymerization. All polymerizations were conducted at $[M]_0$ = 0.25 M in DMAc and 60 ^{o}C.

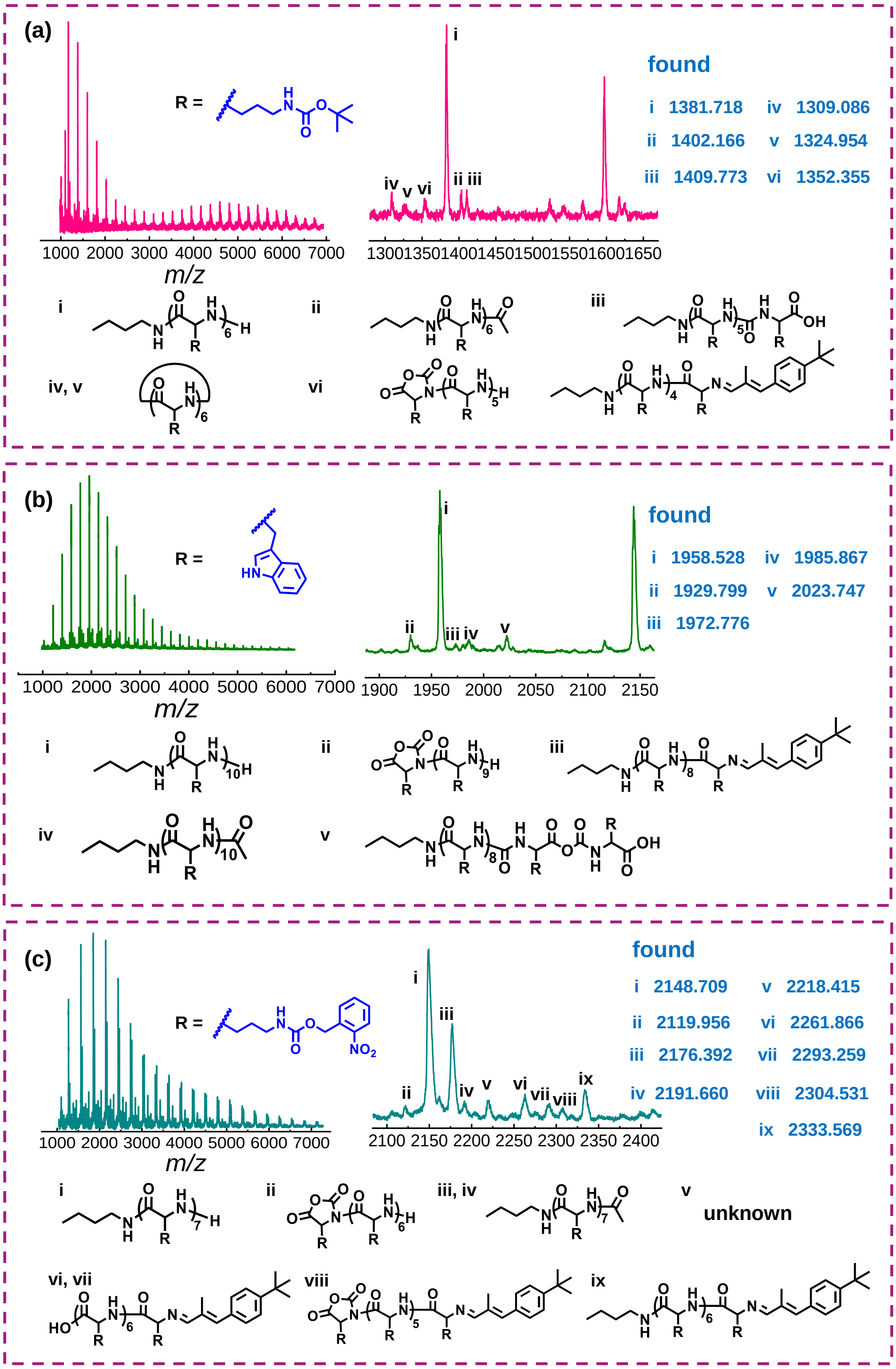

Figure S33. MALDI-TOF MS spectra recorded for (a) PBocO ($[M]_0/[AA]/[I]_0$ = 7/10/1), (b) PTrp ($[M]_0/[AA]/[I]_0$ = 14/10/1), and (c) PNBO ($[M]_0/[AA]/[I]_0$ = 10/10/1) synthesized via polymerization of corresponding NPCA precursors initiated by primary amine in the presence of acetic acid. All polymerizations were conducted at $[M]_0$ = 0.25 M in DMAc at 60 ^{o}C.

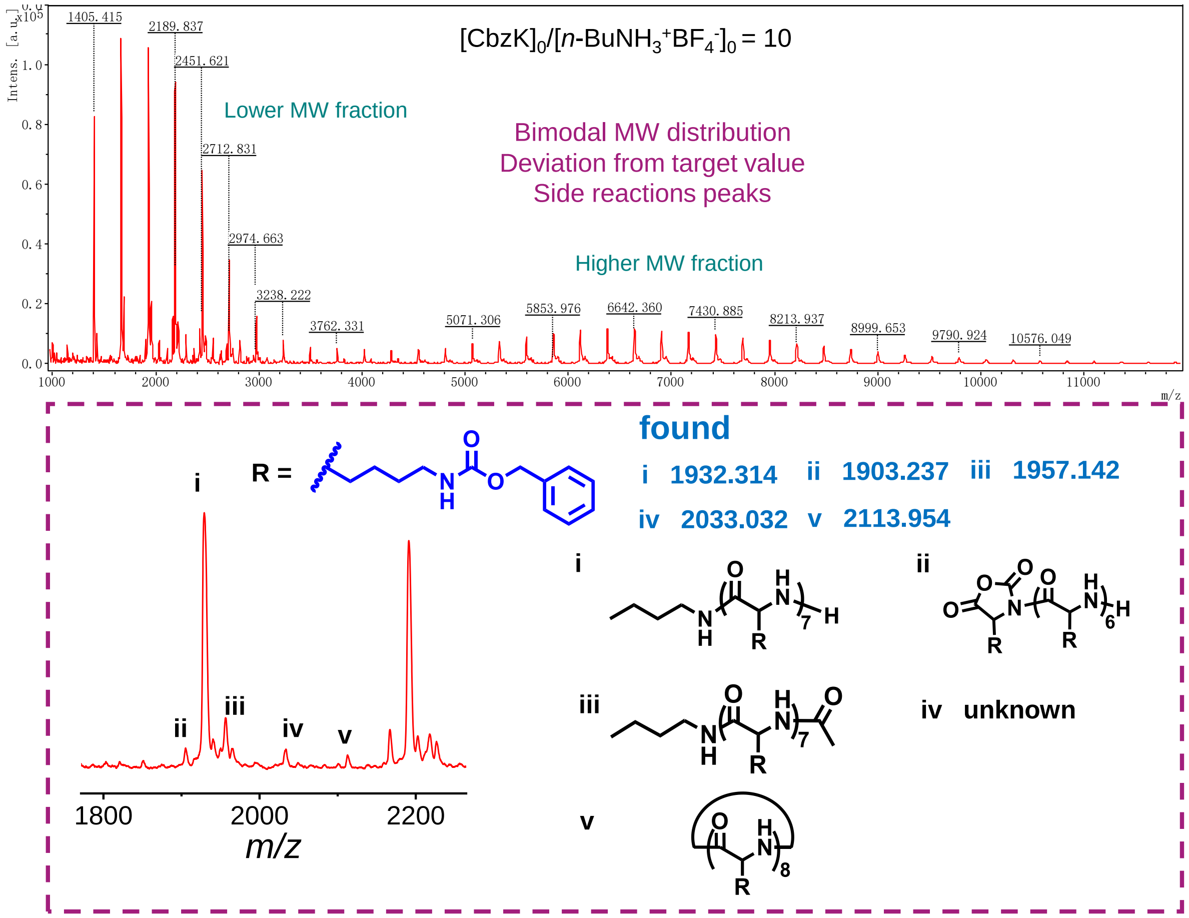

Figure S34. MALDI-TOF MS recorded for PCbzK using n-BuNH$_3^+$BF$_4^-$ as the initiator at a feed ratio of 10. The polymerization was conducted at $[M]_0 = 0.25$ M in DMAc at 70 °C.

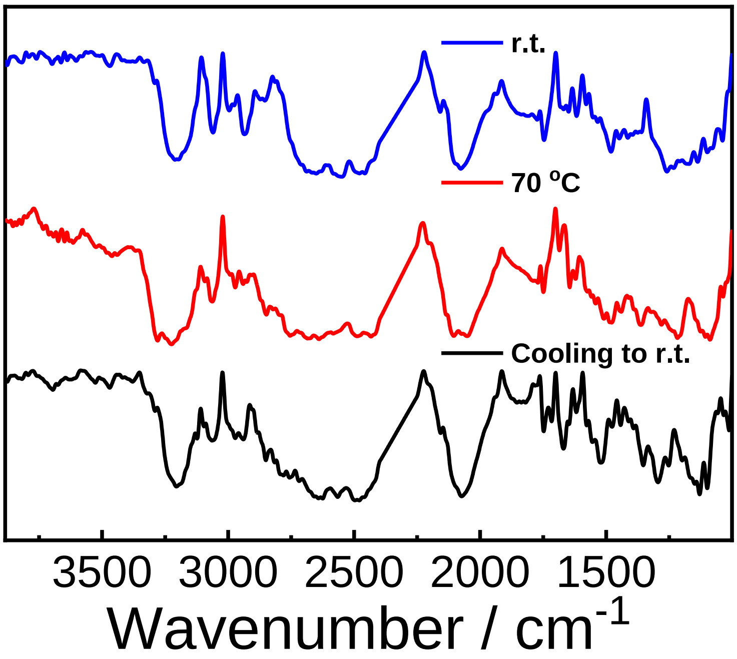

Figure S35. FT-IR spectra recorded for *n*-$BuNH_3^+Cl^-$ initiator in DMAc solvent at varying temperatures.

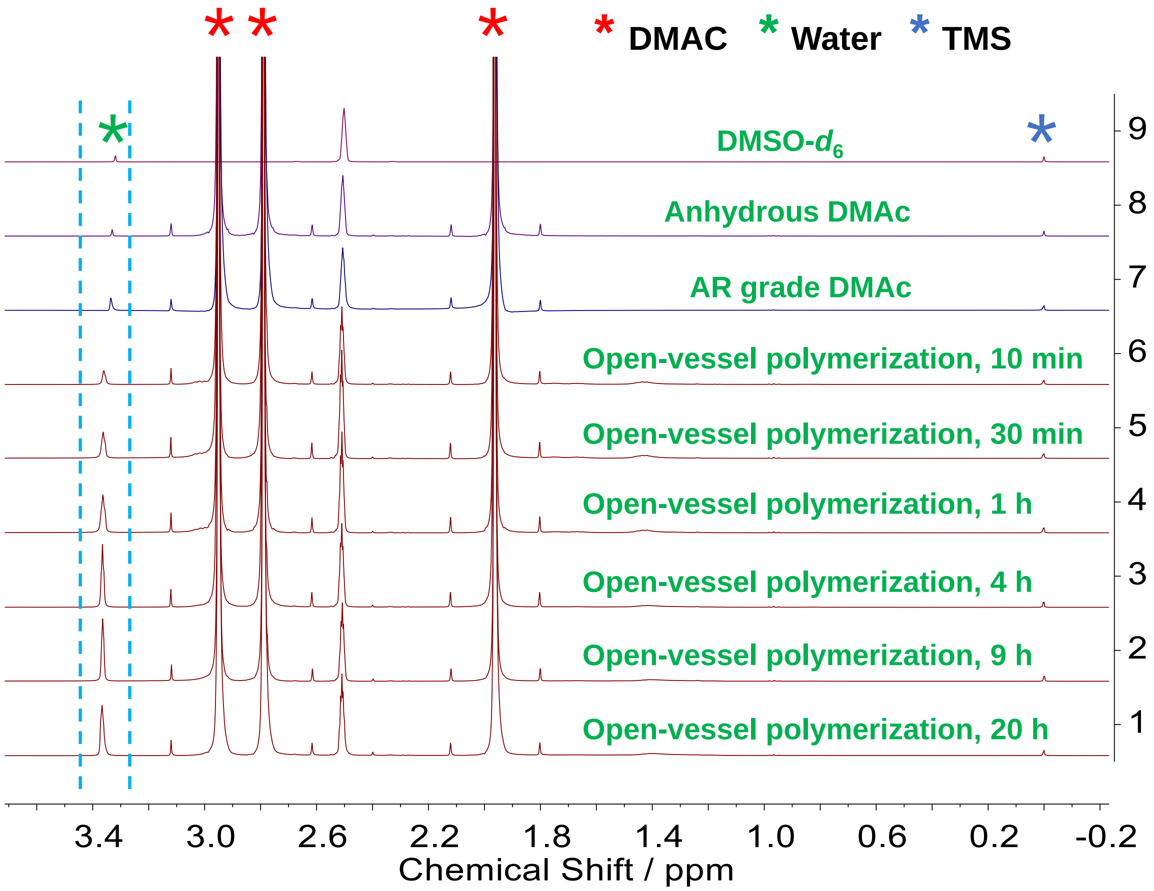

Figure S36. ^{1}H NMR spectra recorded in DMSO-d_6 for commercial anhydrous DMAc, AR grade DMAc, and time-dependent evolution of water contents of DMAc solvent during open-vessel polymerization using n-$BuNH_3^+Cl^-$ as initiator.

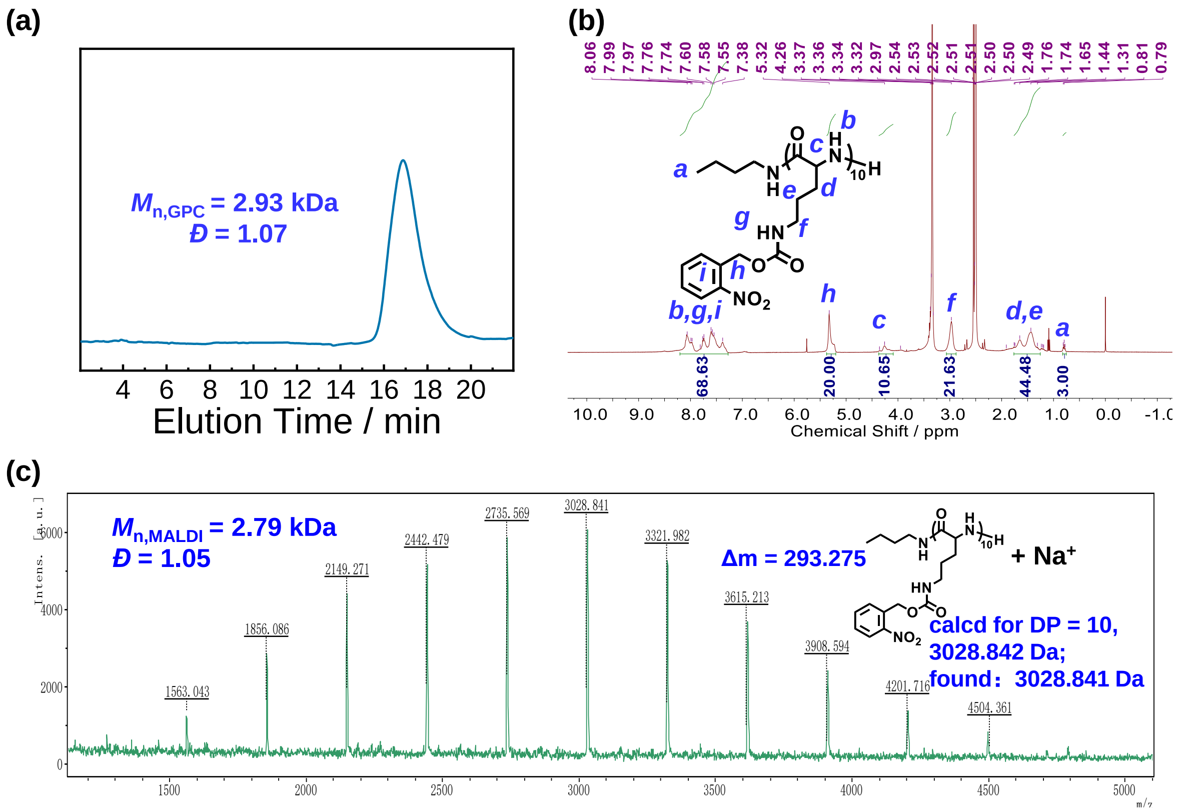

Figure S37. (a) GPC elution trace, (b) ^{1}H NMR spectrum, and (c) MALDI-TOF MS spectrum recorded for $PNBO_{10}$ synthesized in DMAc under open-vessel condition.

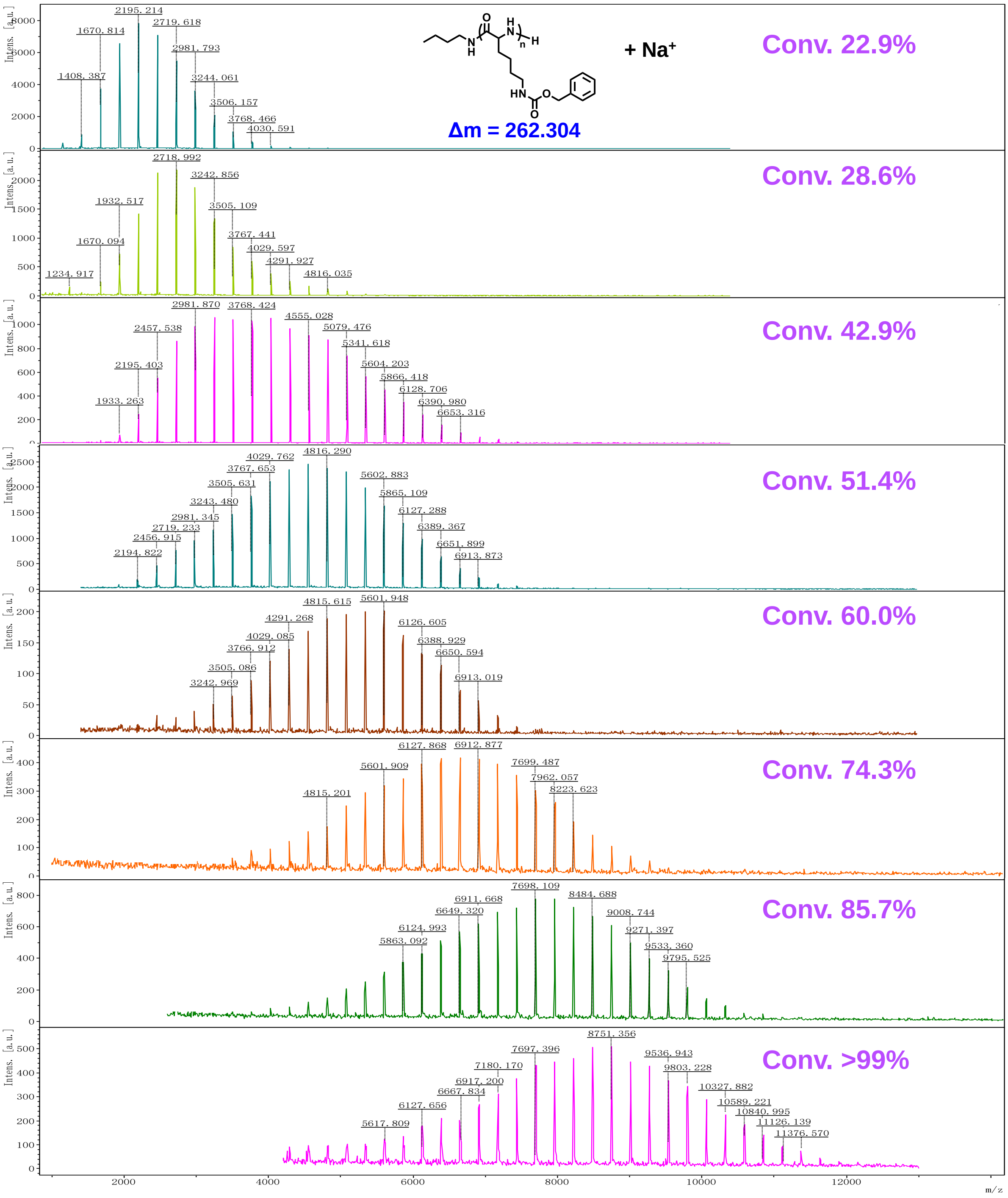

Figure S38. Evolution of MALDI-TOF MS spectra recorded at varying conversions for open-vessel polymerization of CbzK precursor using *n*-$BuNH_3^+Cl^-$ as initiator ($[M]_0/[I]_0 = 35$, $[M]_0 = 0.25$ M, DMAc, 70 °C), revealing the moisture-tolerant nature of primary amine hydrochloride-initiated NPCA polymerization.

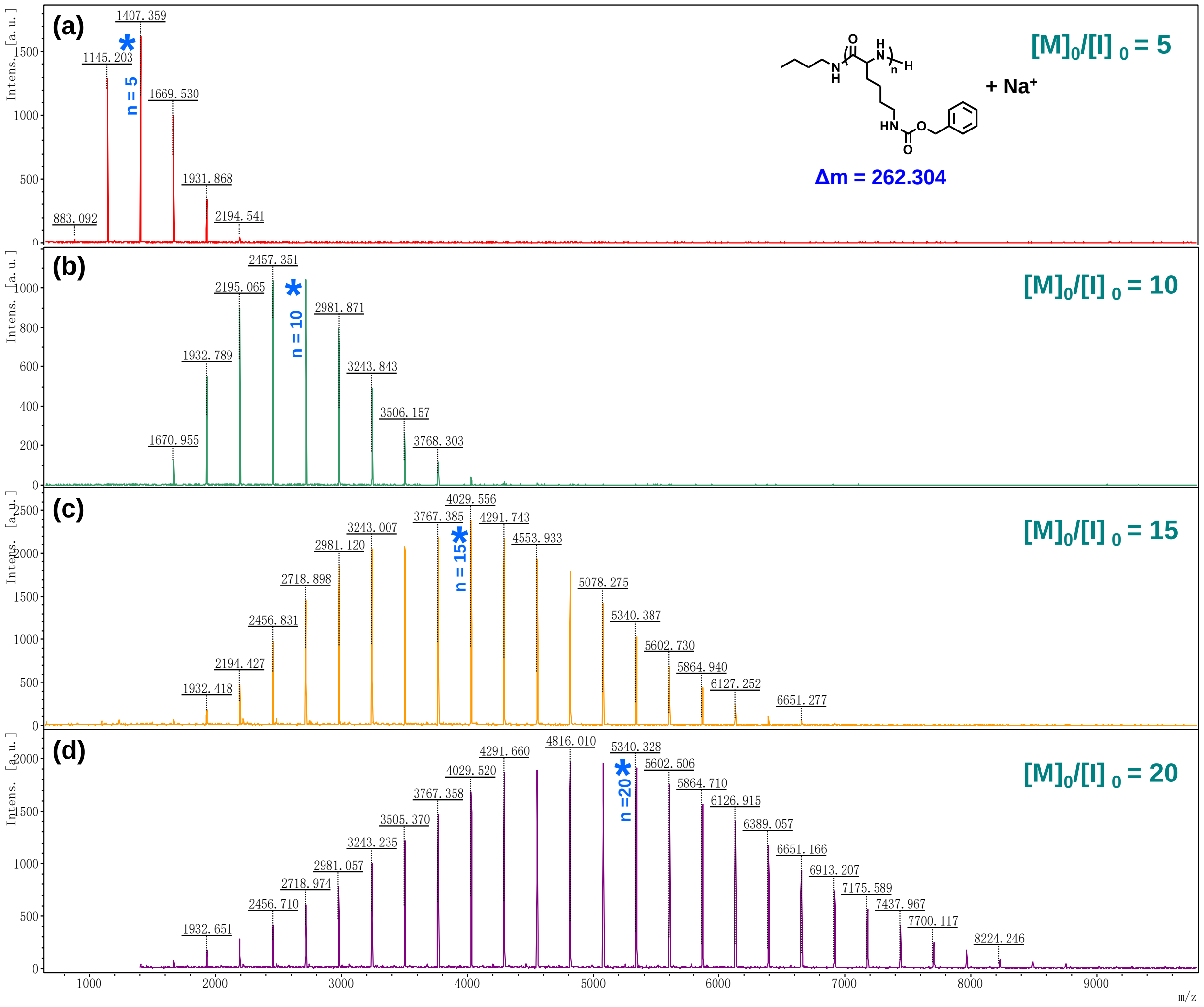

Figure S39. MALDI-TOF MS spectra recorded for PCbzK synthesized under open-vessel condition using *n*-$BuNH_3^+Cl^-$ as initiator at varying $[M]_0/[I]_0$ ratios: (a) 5, (b) 10, (c) 15, (d) 20. All polymerizations were conducted in DMAc at 70 °C and $[M]_0$ = 0.25 M.

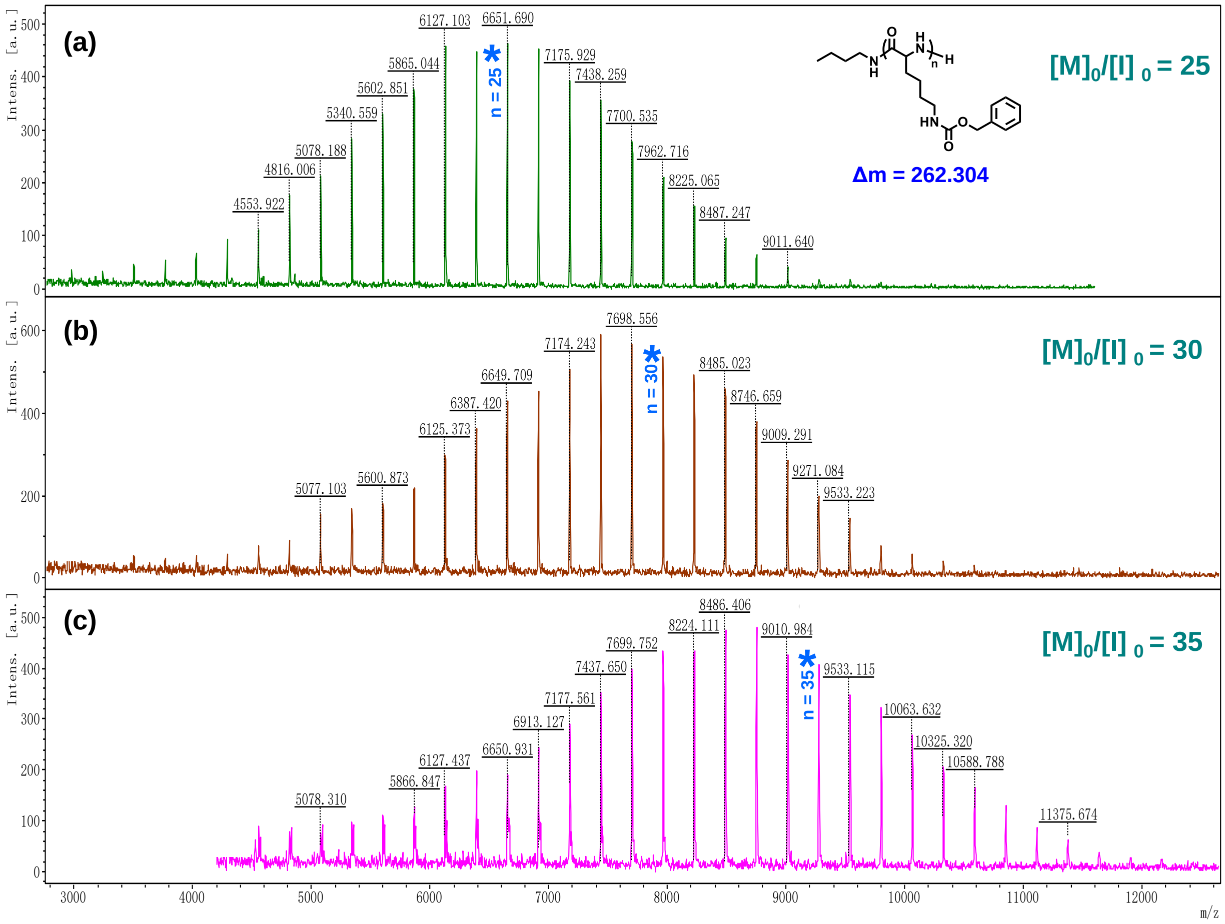

Figure S40. MALDI-TOF MS spectra recorded for PCbzK synthesized under open-vessel condition using *n*-$BuNH_3^+Cl^-$ as initiator at varying $[M]_0/[I]_0$ ratios: (a) 25, (b) 30, (c) 35. All polymerizations were conducted in DMAc at 70 °C and $[M]_0$ = 0.25 M.

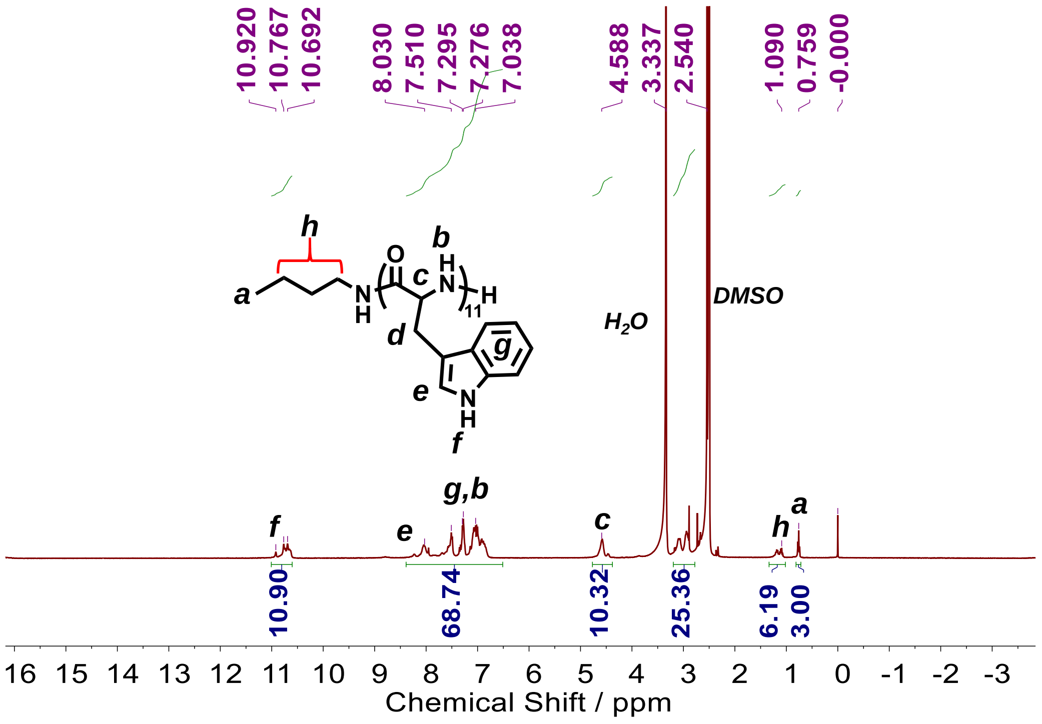

Figure S41. ^{1}H NMR spectrum recorded in DMSO-d_6 for $PTrp_{11}$ synthesized via polymerization of Trp precursor ($[M]_0$ = 0.25 M, DMAc, 70 °C) using n-$BuNH_3^+Cl^-$ as the initiator; during polymerization, the reaction vial was directly exposed to air.

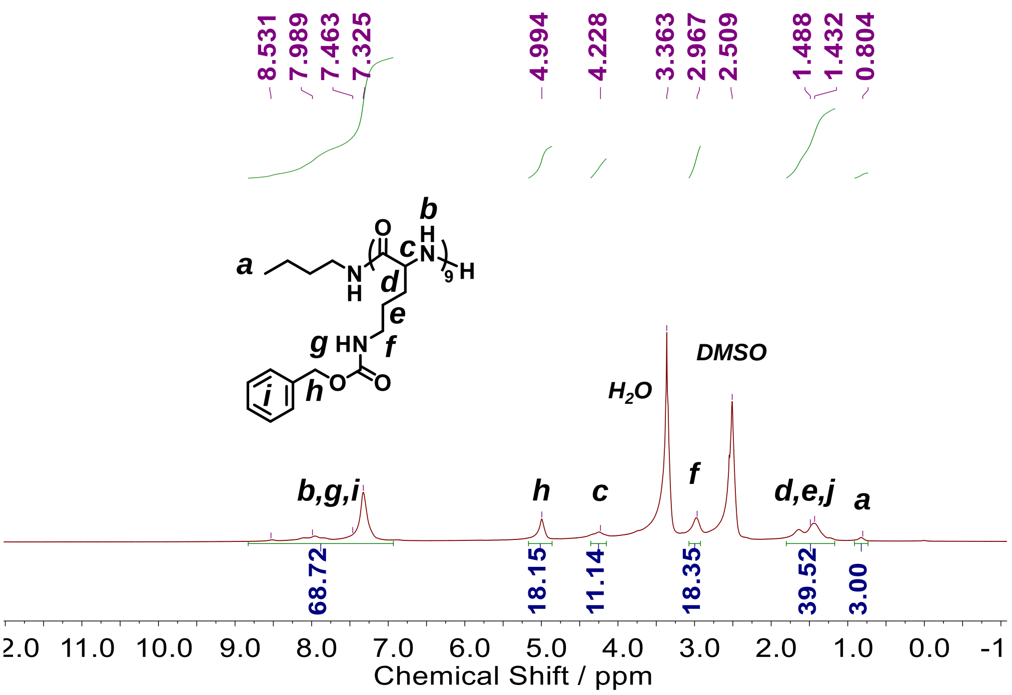

Figure S42. ^{1}H NMR spectrum recorded in DMSO-d_6 for PCbzO$_9$ synthesized via polymerization of CbzO precursor ($[M]_0$ = 0.25 M, DMAc, 70 °C) using *n*-$BuNH_3^+Cl^-$ as the initiator; during polymerization, the reaction vial was directly exposed to air.

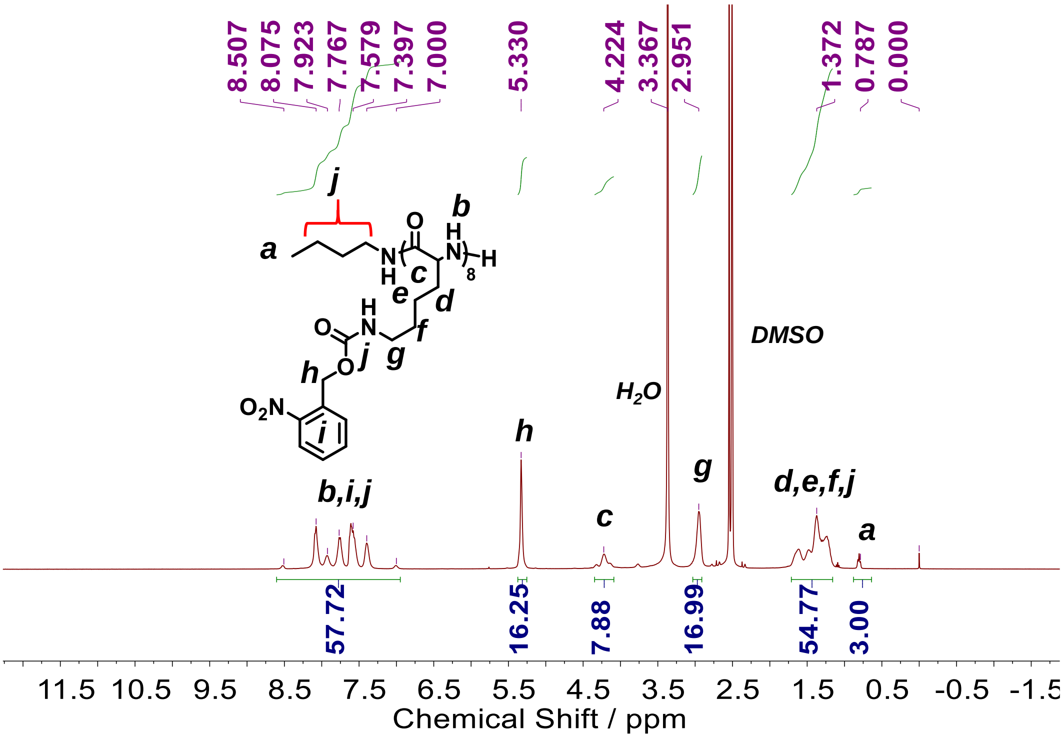

Figure S43. ^{1}H NMR spectrum recorded in DMSO-d_6 for $PNBK_8$ synthesized via polymerization of NBK precursor ($[M]_0$ = 0.25 M, DMAc, 70 °C) using *n*-$BuNH_3^+Cl^-$ as the initiator; during polymerization, the reaction vial was directly exposed to air.

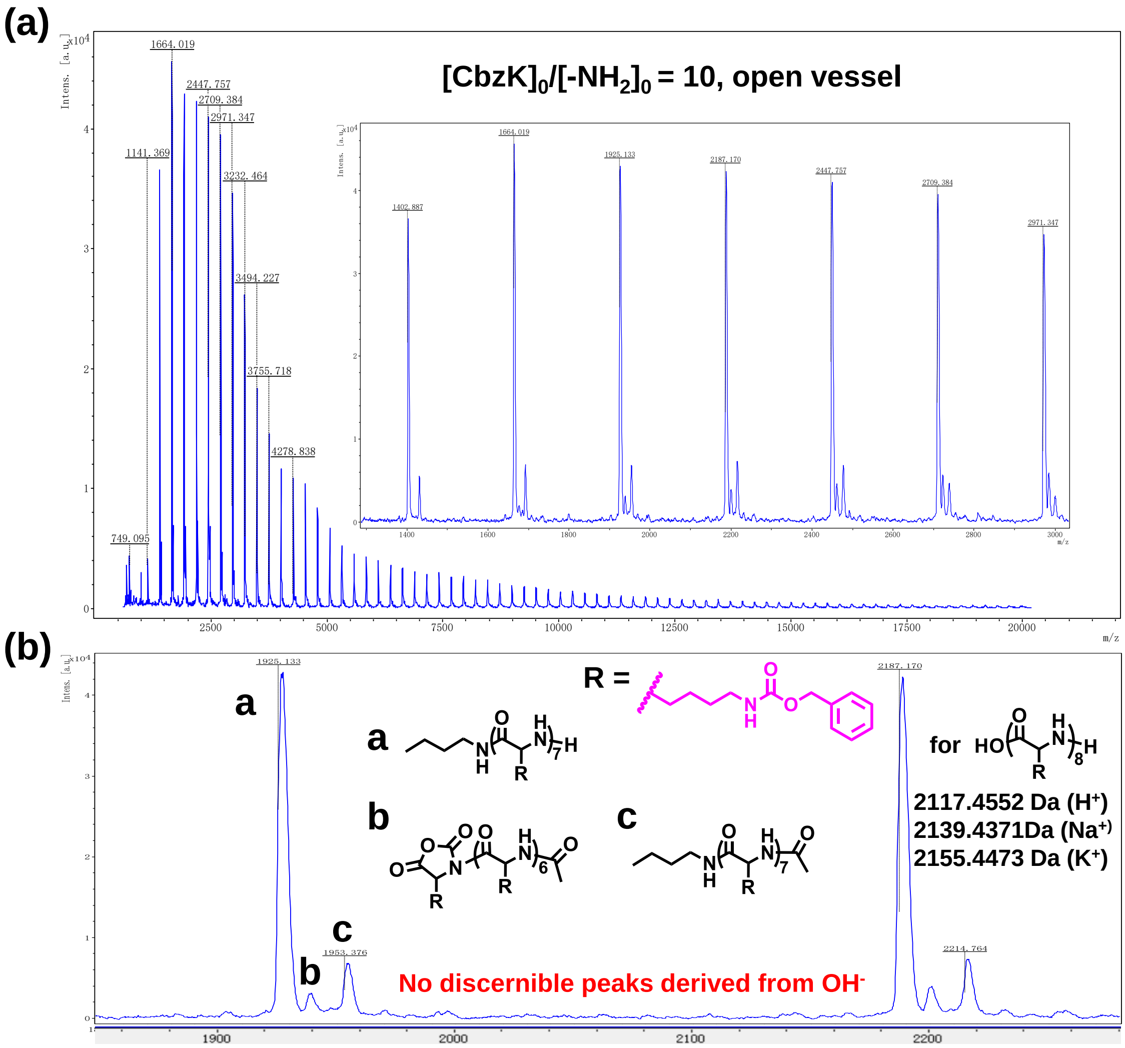

Figure S44. (a) MALDI-TOF MS spectrum recorded for CbzK polymerization under open-vessel condition using *n*-BuNH$_2$ initiator. (b) The zoom-in view of MALDI-TOF MS spectrum. The polymerization was conducted in DMAc at 60 ^{o}C and $[M]_0$ = 0.25 M.

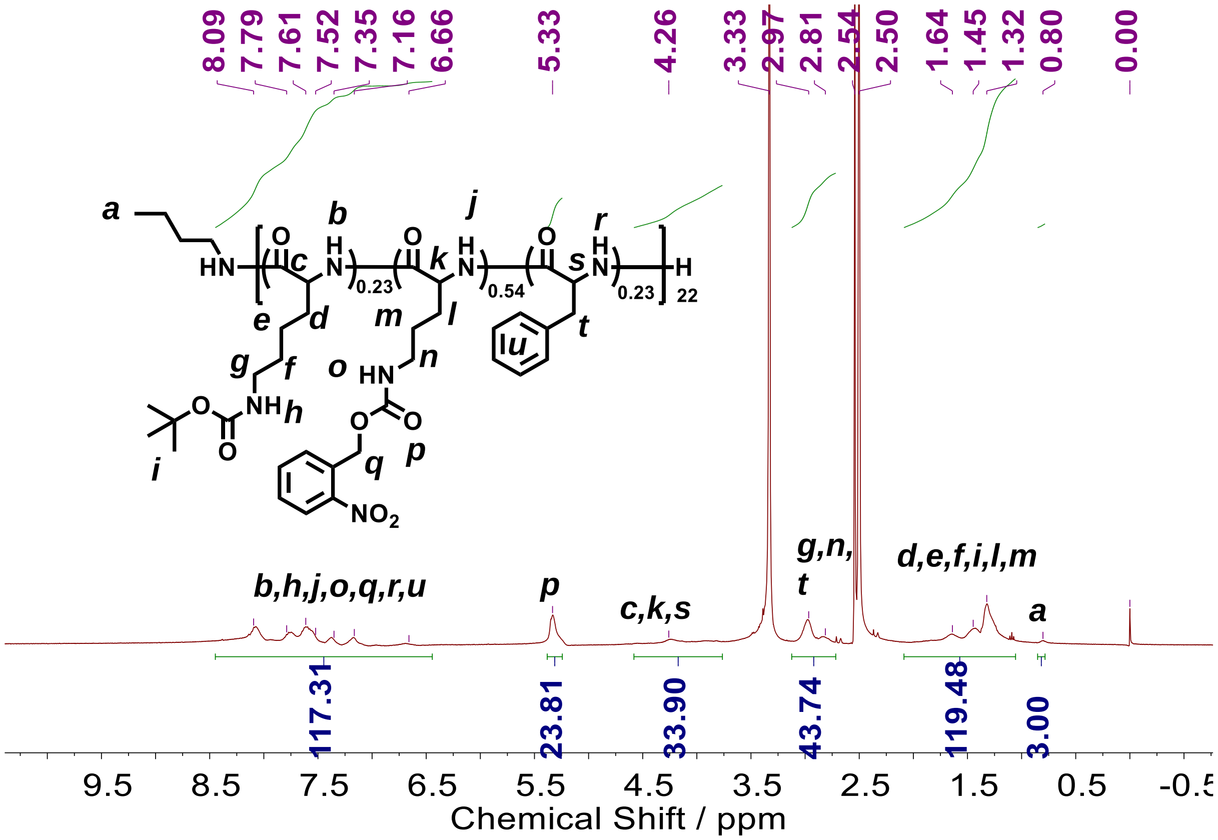

Figure S45. ^{1}H NMR spectrum recorded in DMSO-d_6 for Poly(BocK$_{0.23}$-*r*-NBO$_{0.54}$-*r*-Phe$_{0.23}$)$_{22}$ synthesized via one-pot random copolymerization of BocK, NBO, and Phe precursors ([M]$_0$ = 0.25 M, DMAc, 70 ^{o}C) using *n*-BuNH$_3^+$Cl$^-$ as the initiator under open-vessel condition.

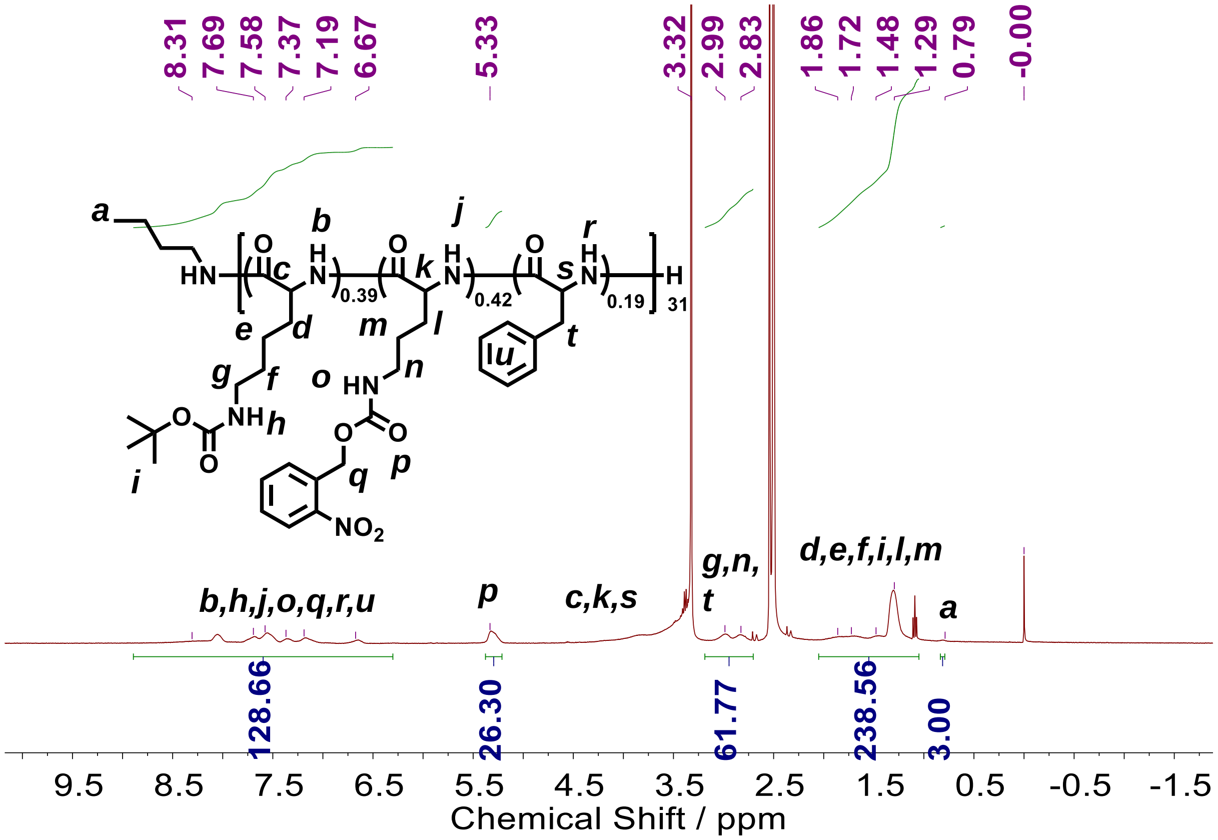

Figure S46. ^{1}H NMR spectrum recorded in DMSO-d_6 for Poly(BocK$_{0.39}$-*r*-NBO$_{0.42}$-*r*-Phe$_{0.19}$)$_{31}$ synthesized via one-pot random copolymerization of BocK, NBO, and Phe precursors ($[M]_0$ = 0.25 M, DMAc, 70 °C) using *n*-$BuNH_3^+Cl^-$ as the initiator under open-vessel condition.

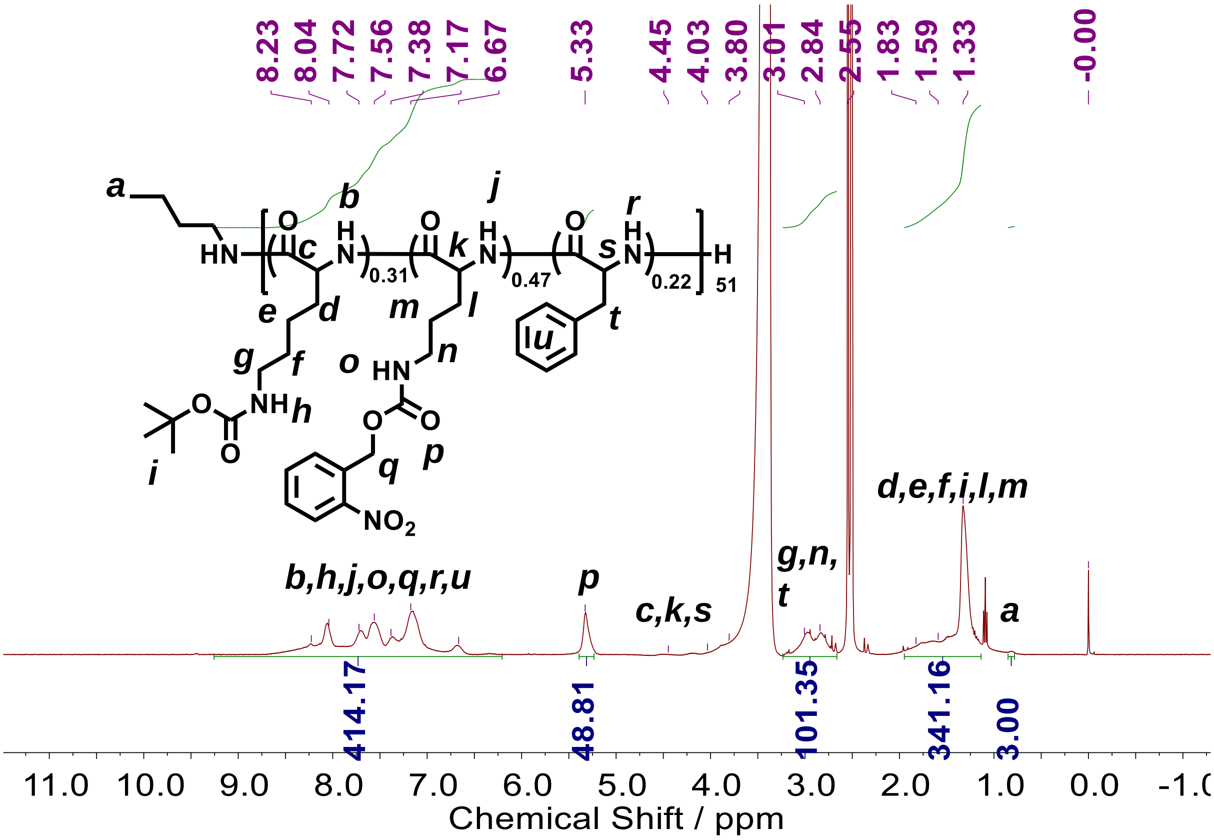

Figure S47. ^{1}H NMR spectrum recorded in DMSO-d_6 for Poly(BocK$_{0.31}$-*r*-NBO$_{0.47}$-*r*-Phe$_{0.22}$)$_{51}$ synthesized via one-pot random copolymerization of BocK, NBO, and Phe precursors ($[M]_0$ = 0.25 M, DMAc, 70 °C) using *n*-$BuNH_3^+Cl^-$ as the initiator under open-vessel condition.

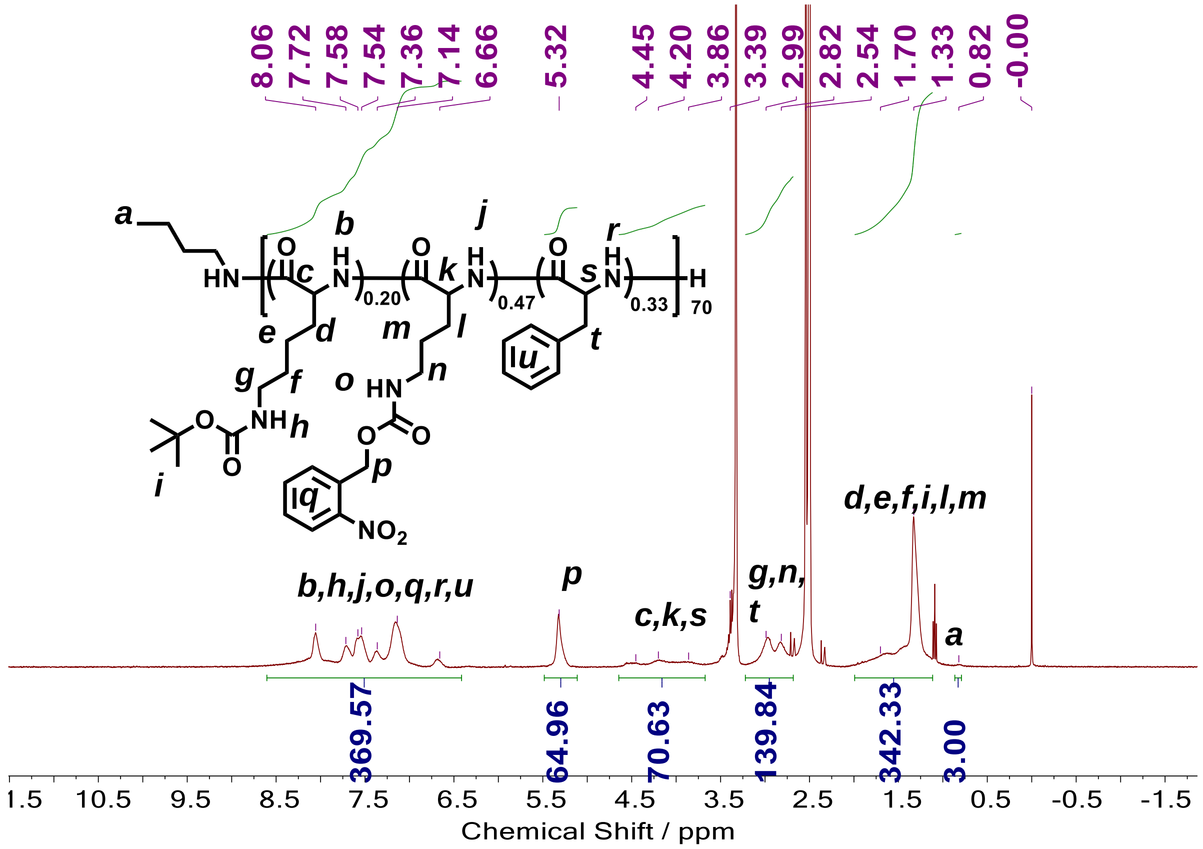

Figure S48. ^{1}H NMR spectrum recorded in DMSO-d_6 for Poly(BocK$_{0.20}$-*r*-NBO$_{0.47}$-*r*-Phe$_{0.33}$)$_{70}$ synthesized via one-pot random copolymerization of BocK, NBO, and Phe precursors ([M]$_0$ = 0.25 M, DMAc, 70 °C) using *n*-BuNH$_3^+$Cl$^-$ as the initiator under open-vessel condition.

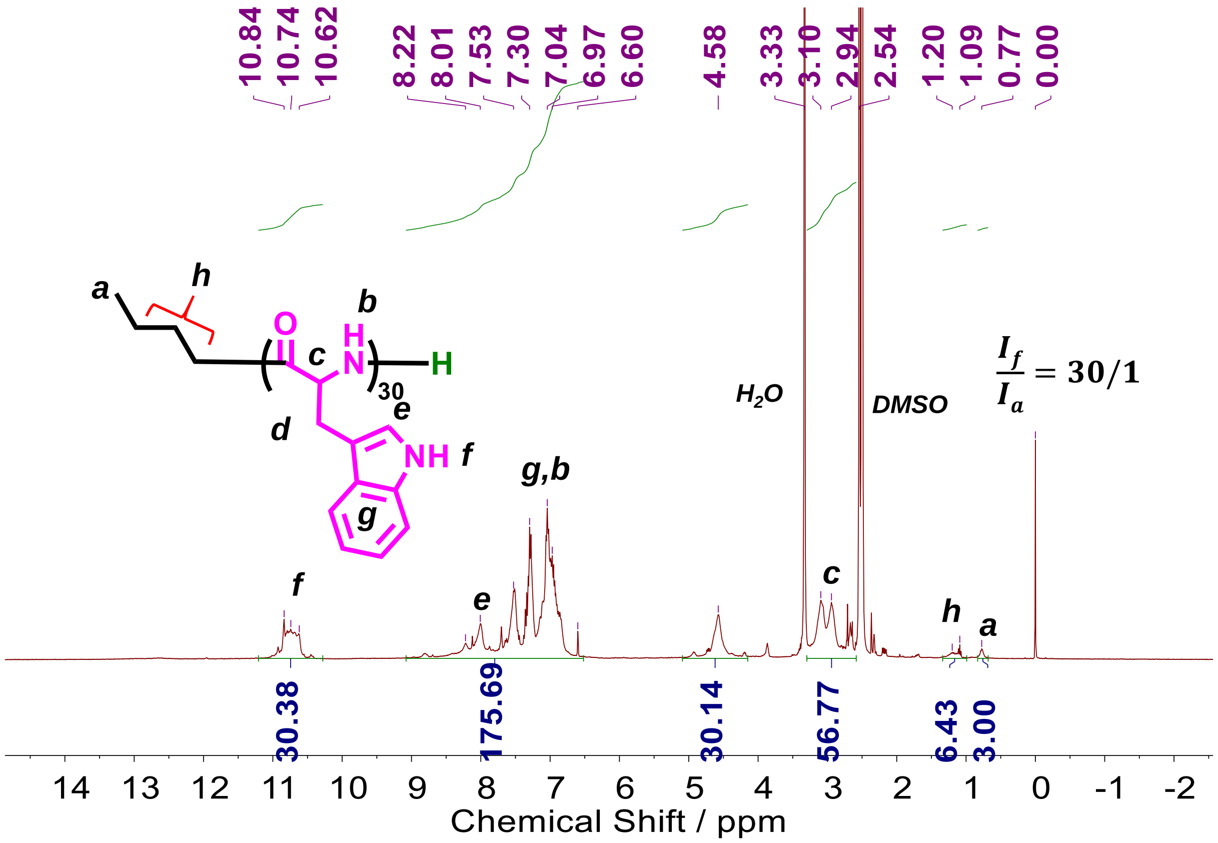

Figure S49. ^{1}H NMR spectrum recorded in DMSO-d_6 for $PTrp_{30}$ synthesized via polymerization of Trp precursor ($[M]_0$ = 0.25 M, DMAc, 70 °C) using n-$BuNH_3^+Cl^-$ as the initiator in the open vessel exposed to air.

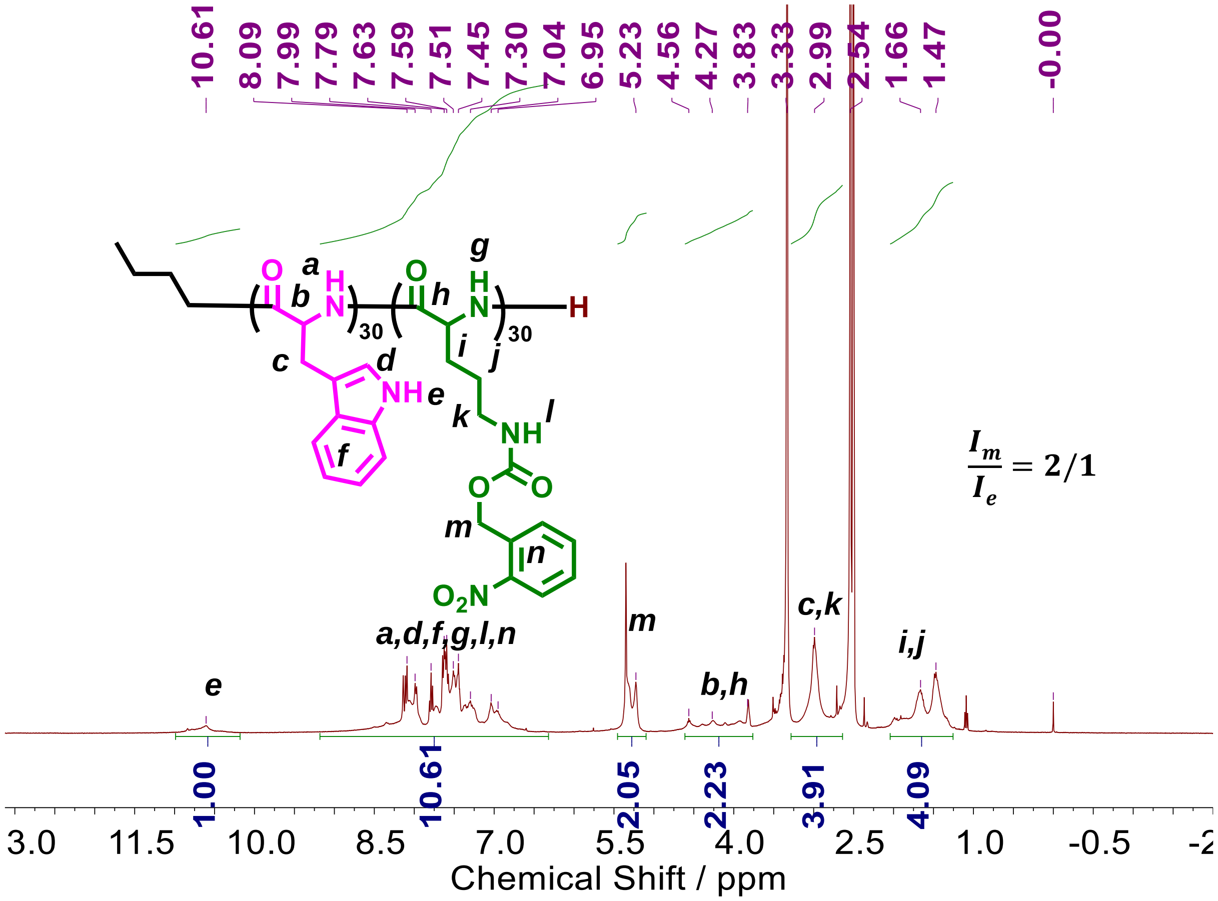

Figure S50. ^{1}H NMR spectrum recorded in DMSO-d_6 for PTrp$_{30}$-*b*-PNBO$_{30}$ diblock copolypeptide synthesized via sequential polymerizations of Trp and NBO precursors ($[M]_0$ = 0.25 M, DMAc, 70 °C) using *n*-BuNH$_3^+$Cl$^-$ as the initiator in the open vessel exposed to air.

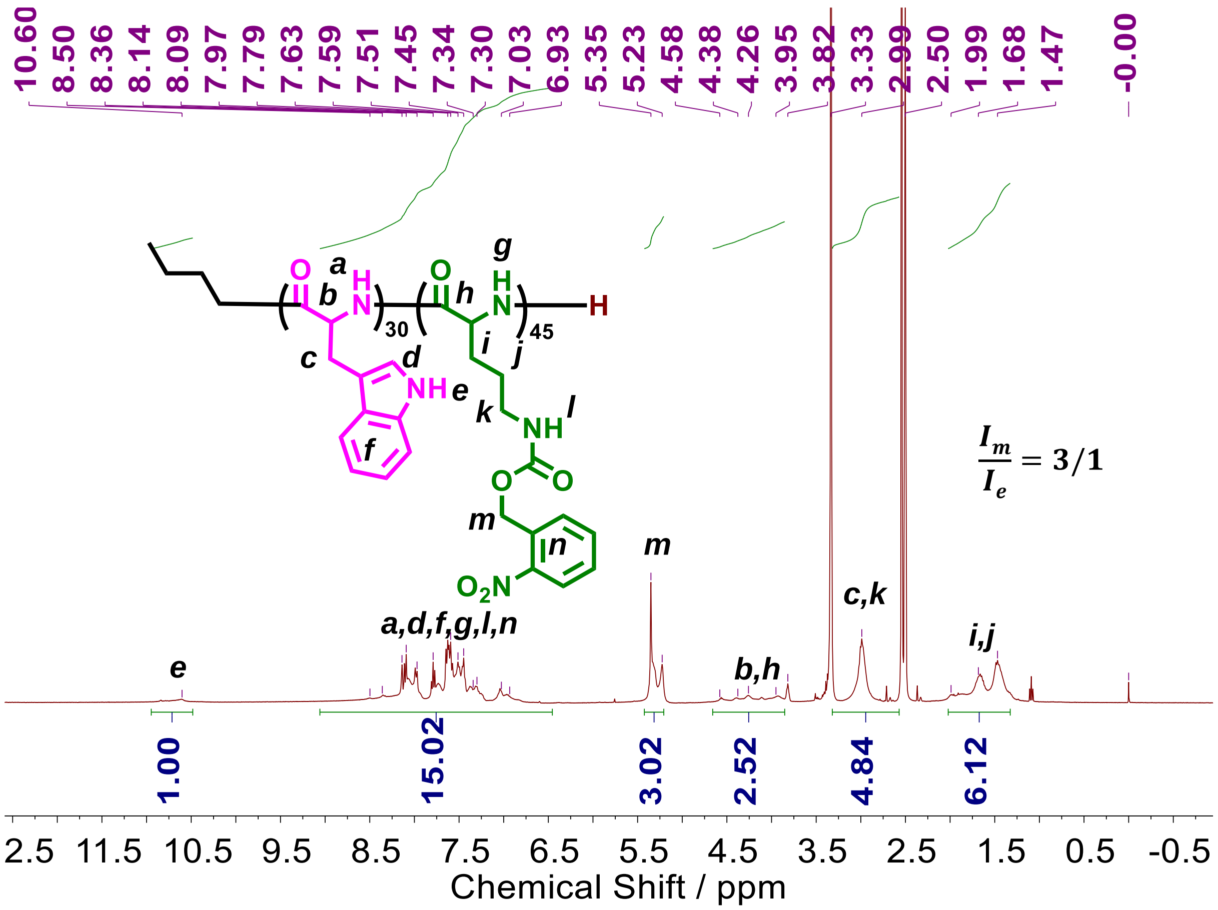

Figure S51. ^{1}H NMR spectrum recorded in DMSO-d_6 for PTrp$_{30}$-*b*-PNBO$_{45}$ diblock copolypeptide via sequential polymerizations of Trp and NBO precursors ($[M]_0$ = 0.25 M, DMAc, 70 °C) using *n*-$BuNH_3^+Cl^-$ as the initiator in the open vessel exposed to air.

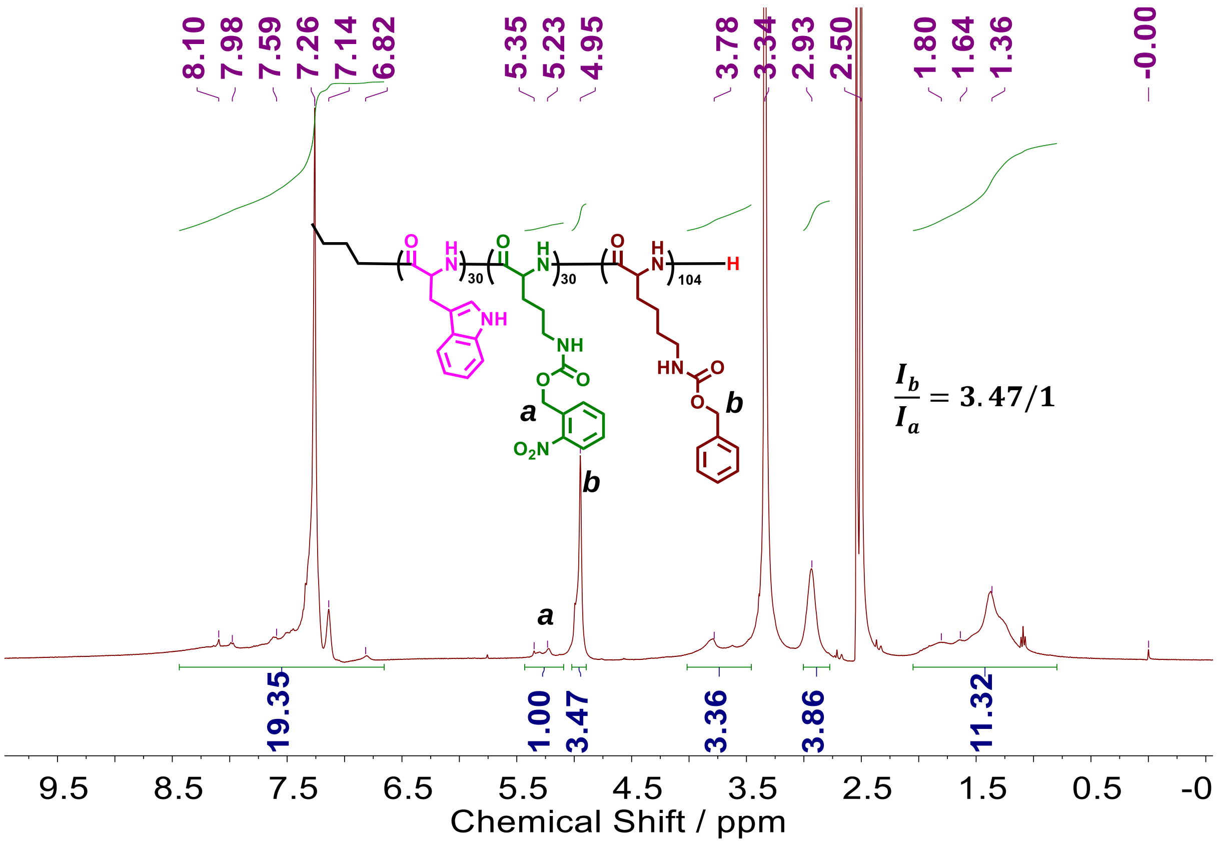

Figure S52. ^{1}H NMR spectrum recorded in DMSO-d_6 for PTrp$_{30}$-*b*-PNBO$_{30}$-*b*-PCbzK$_{104}$ triblock copolypeptide via sequential polymerizations of Trp, NBO, and CbzK precursors ($[M]_0$ = 0.25 M, DMAc, 70 °C) using *n*-BuNH$_3^+$Cl$^-$ as the initiator in the open vessel exposed to air.

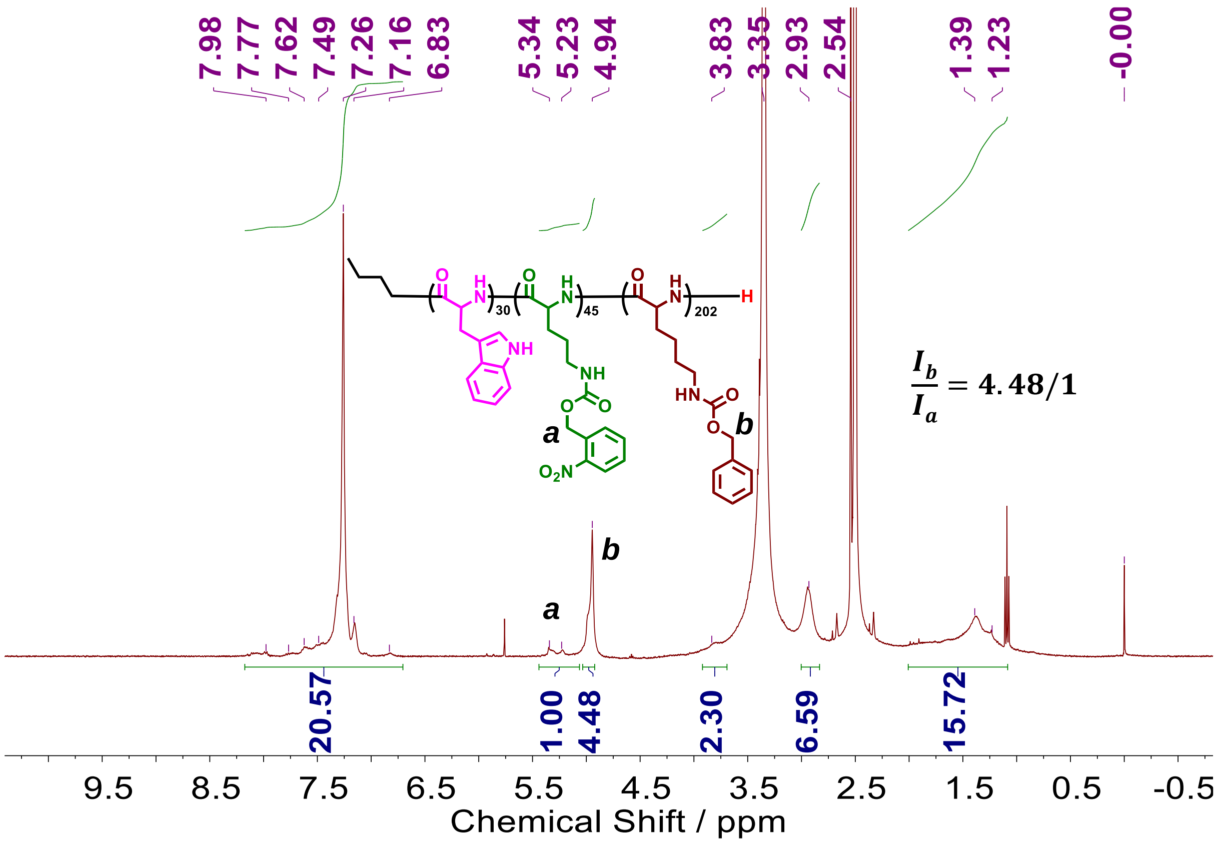

Figure S53. ^{1}H NMR spectrum in DMSO-d_6 recorded for PTrp$_{30}$-*b*-PNBO$_{45}$-*b*-PCbzK$_{202}$ triblock copolypeptide via sequential polymerizations of Trp, NBO, and CbzK precursors ($[M]_0$ = 0.25 M, DMAc, 70 °C) using *n*-$BuNH_3^+Cl^-$ as the initiator in the open vessel exposed to air.

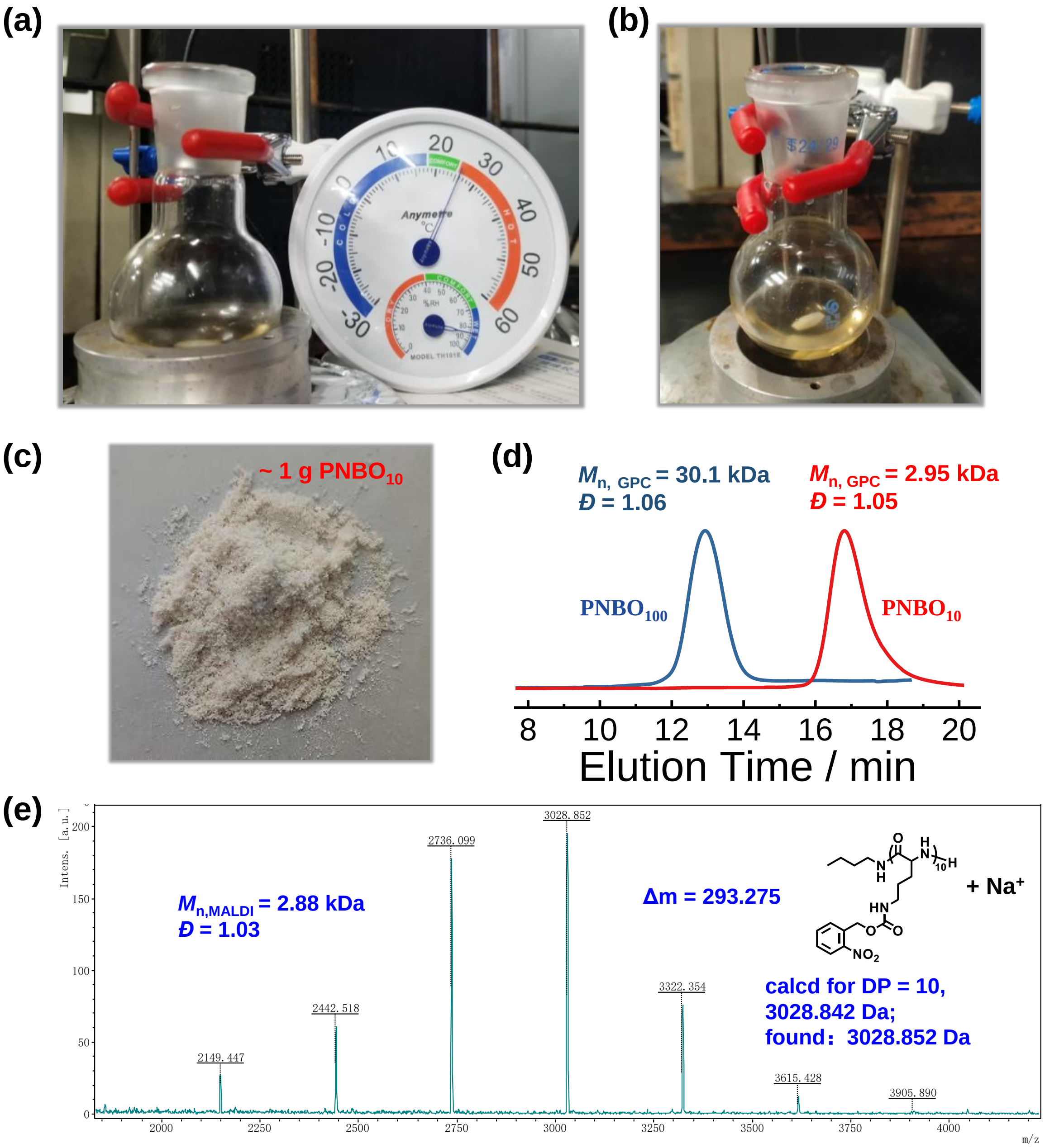

Figure S54. (a,b,c) Schematics illustrating gram scale synthesis of PNBO polypeptide initiated by *n*-$BuNH_3^+Cl^-$ under open-vessel condition. (d) GPC elution traces recorded for $PNBO_{10}$ and $PNBO_{100}$. (e) MALDI-TOF MS spectrum recorded for $PNBO_{10}$ synthesized via open-vessel polymerization of NBO precursor at gram scale using *n*-$BuNH_3^+Cl^-$ as initiator ($[M]_0$ = 0.25 M, DMAc, 70 °C).

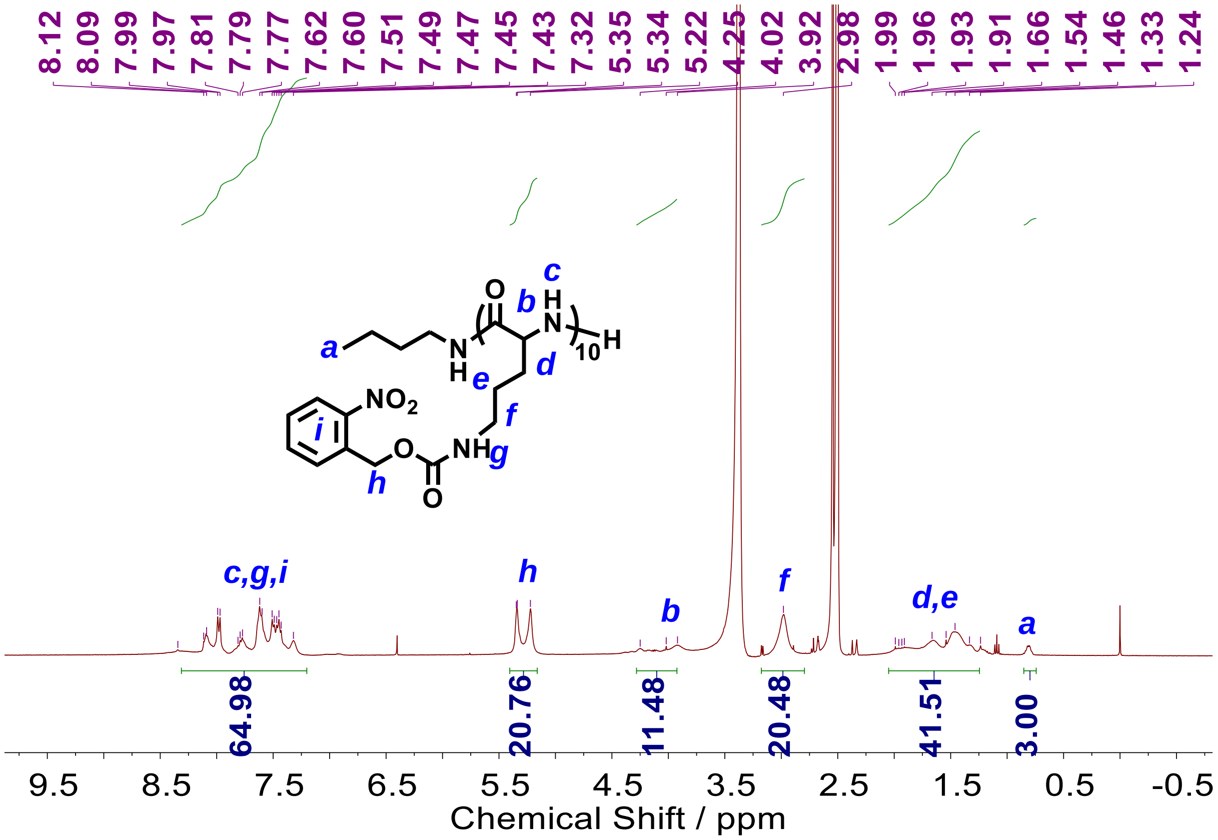

Figure S55. ^{1}H NMR spectrum recorded in DMSO-d_6 for $PNBO_{10}$ synthesized via open-vessel polymerization of NBO precursor ($[M]_0$ = 0.25 M, DMAc, 70 °C) at gram scale using *n*-$BuNH_3^+Cl^-$ as the initiator.

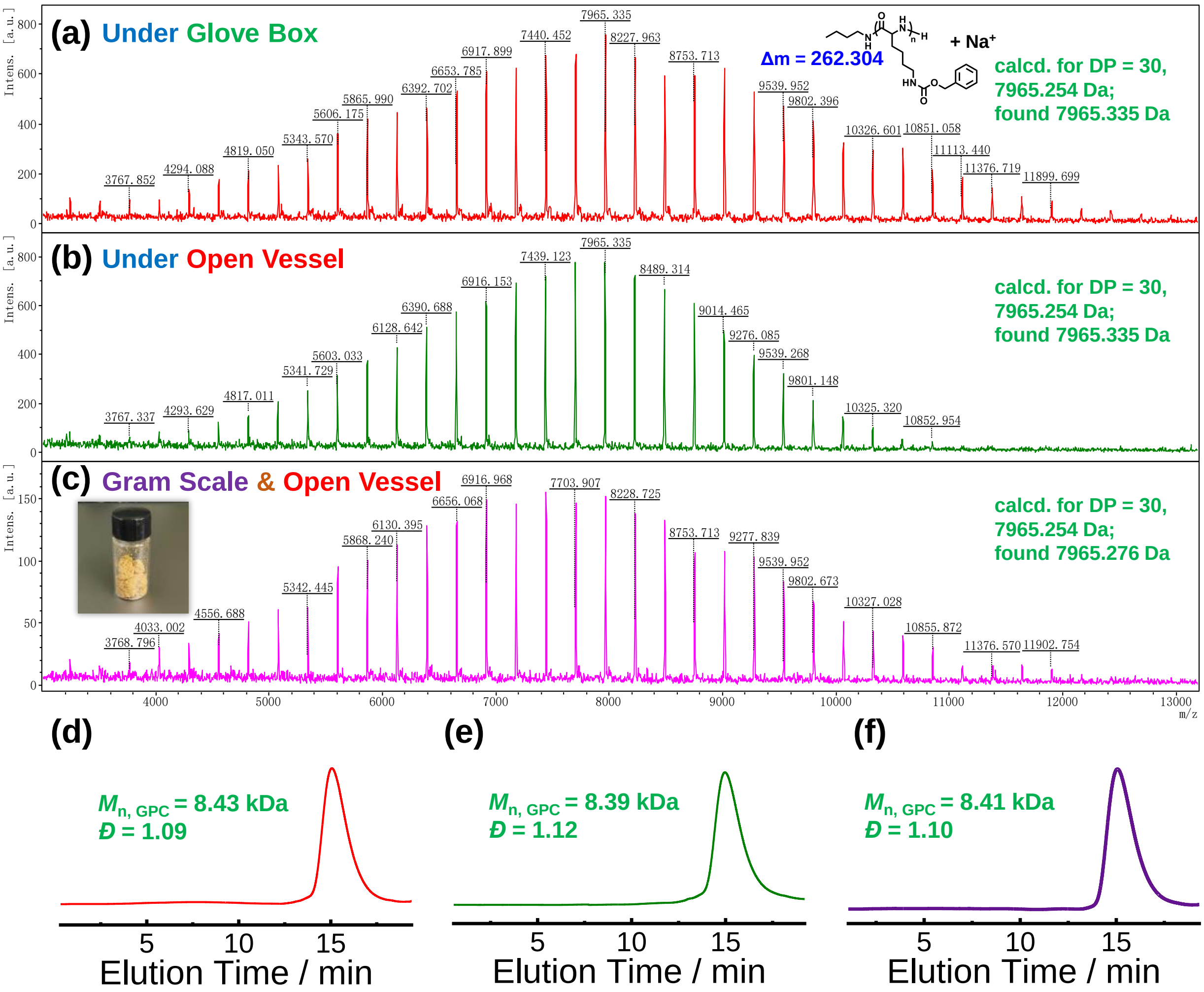

Figure S56. MALDI-TOF MS spectra and corresponding GPC elution traces recorded for PCbzK polypeptide synthesized under different conditions ($[M]_0/[I]_0 = 30$): (a,d) inside glove box, tens of milligram scale, (b,e) open-vessel, tens of milligram scale, (c,f) open-vessel, gram scale. All polymerizations were conducted at $[M]_0 = 0.25$ M in DMAc and 70 ^{o}C.

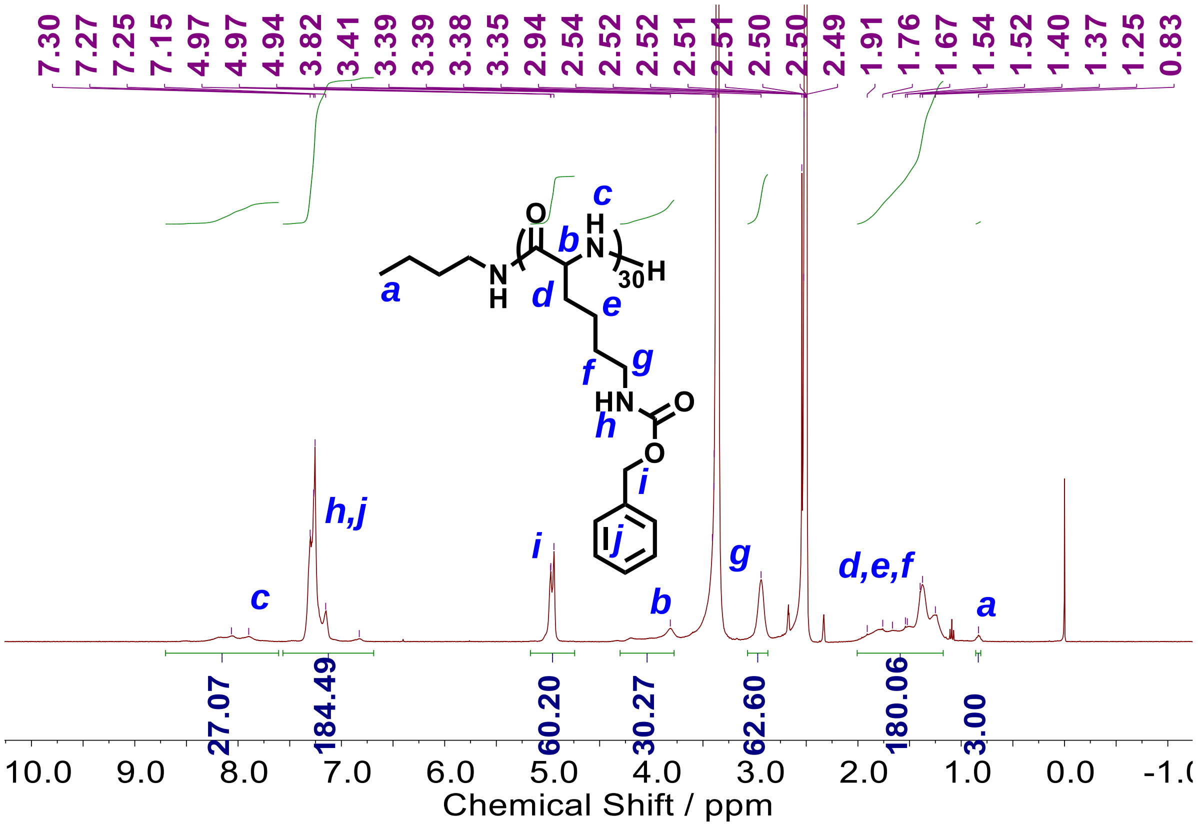

Figure S57. ^{1}H NMR spectrum recorded in DMSO-d_6 for $PCbzK_{30}$ synthesized via open-vessel polymerization of CbzK precursor ($[M]_0$ = 0.25 M, DMAc, 70 ºC) at gram scale using *n*-$BuNH_3^+Cl^-$ as the initiator.

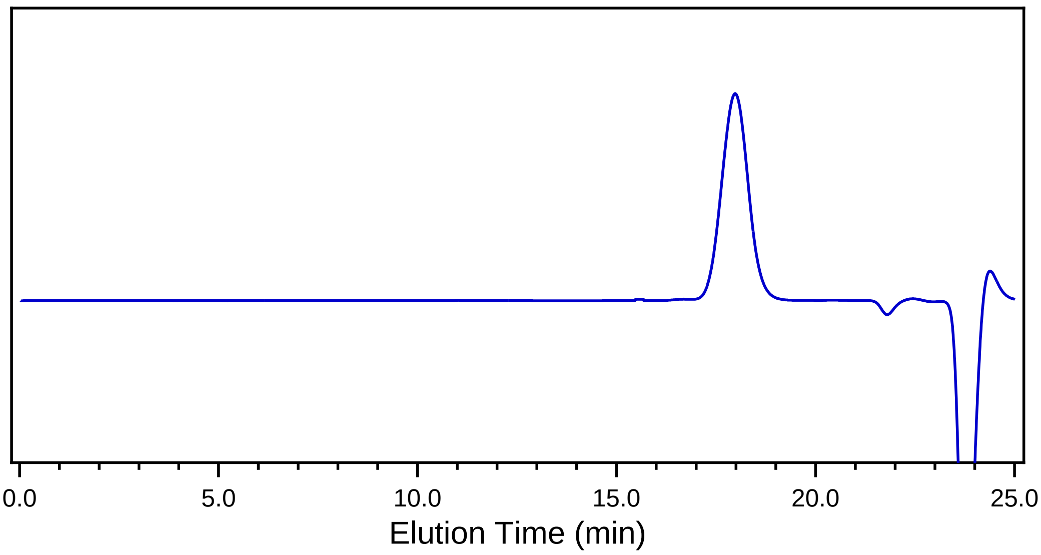

Figure S58. GPC raw data of PCbzK polypeptides synthesized at $[M]_0/[I]_0$ = 10 using *n*-$BuNH_3^+Cl^-$ as initiator under open vessel.

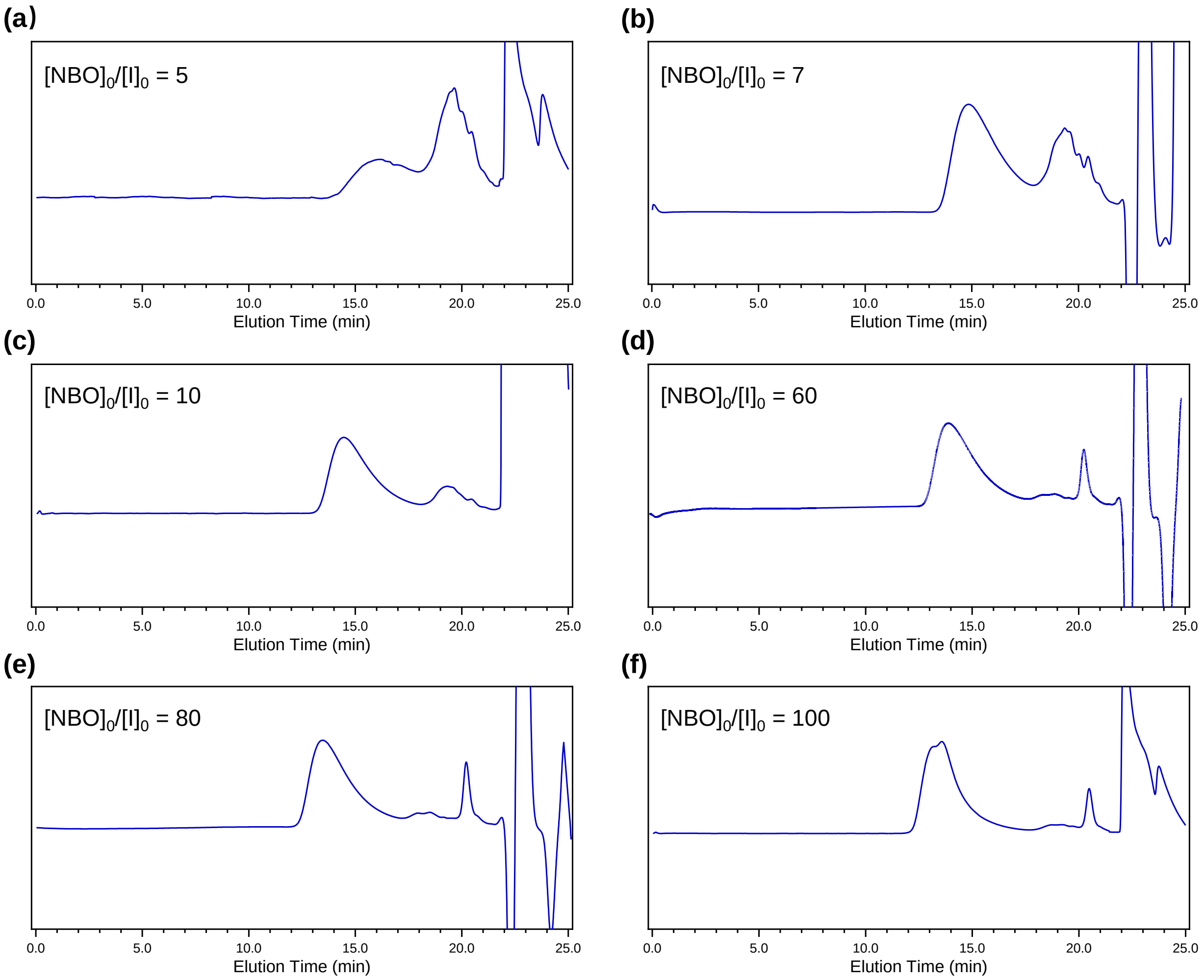

Figure S59. GPC raw data of PNBO via polymerization of NBO precursor ($[M]_0$ = 0.25 M, DMAc, 60 ^{o}C) using *n*-$BuNH_2$ as the initiator at different $[NBO]_0/[I]_0$ ratios.

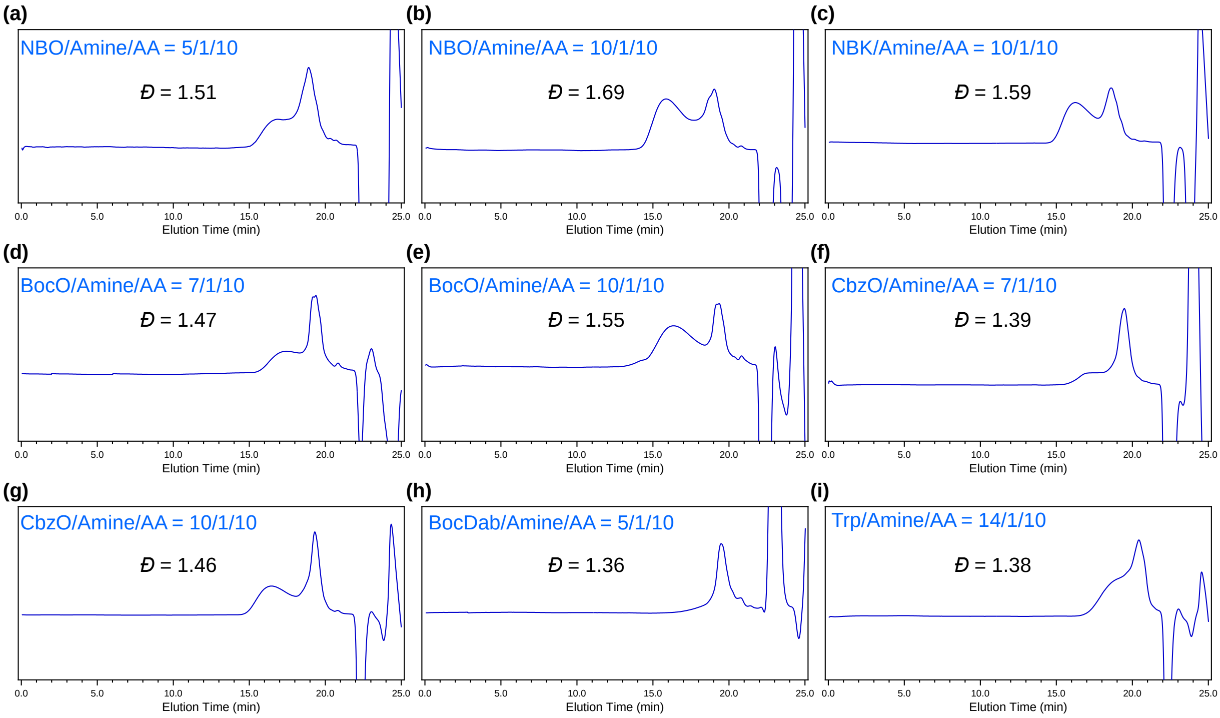

Figure S60. GPC raw data of polypeptides synthesized via polymerization of a variety of NPCA precursors using *n*-BuNH$_2$ as initiator in the presence of acetic acid ([AA]/[I] = 10/1, $[M]_0$ = 0.25 M, DMAc, 60 ^oC), revealing bimodal GPC elution traces for polypeptides synthesized at $[M]_0/[I]_0$ feed ratios in the range of 5-14.

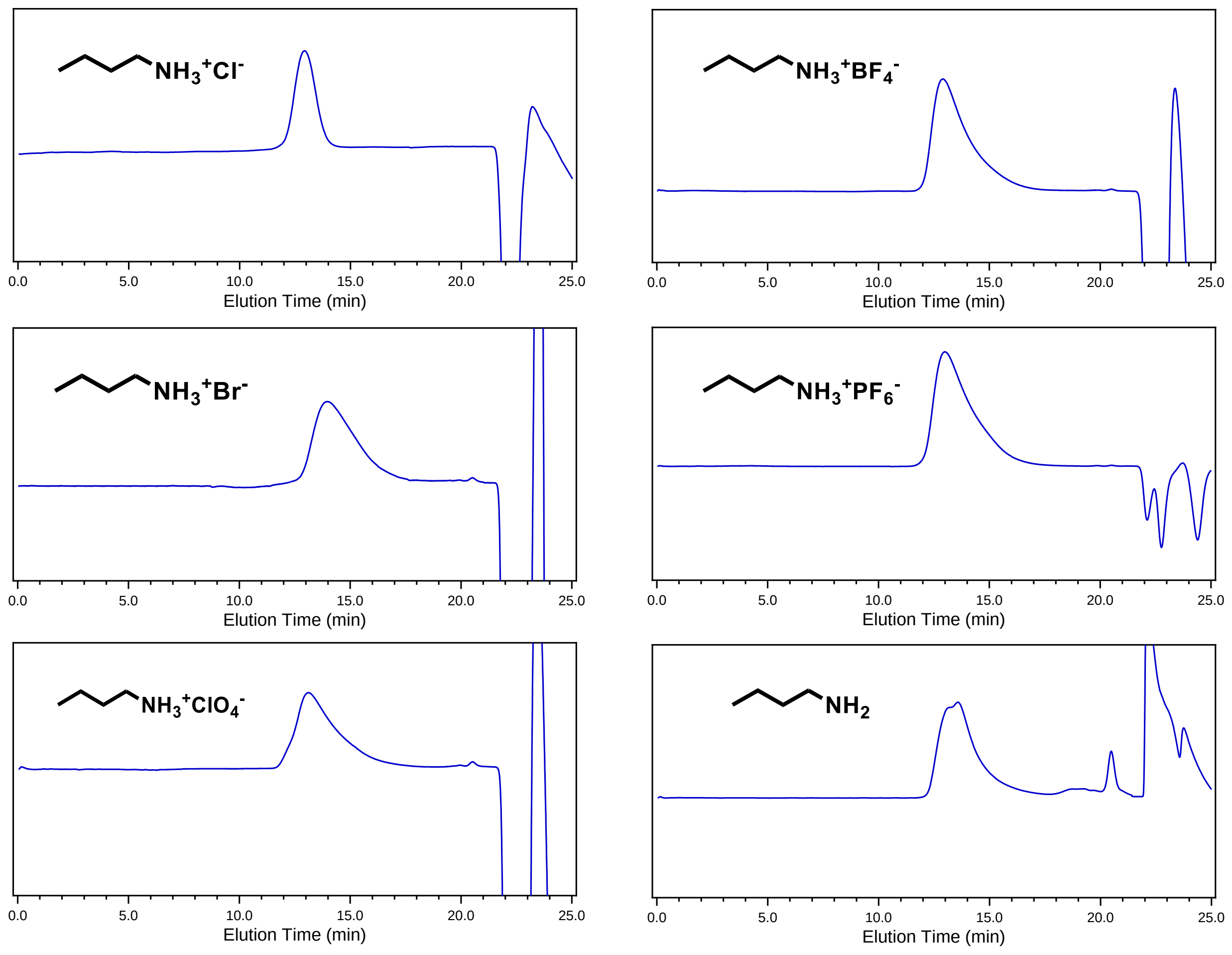

Figure S61. GPC raw data of PNBO polypeptides synthesized using *n*-$BuNH_3^+X^-$ initiators and *n*-$BuNH_2$ initiator with varying counter ions (X^- refers to Cl^-, BF_4^-, Br^-, PF_6^-, and ClO_4^-; $[M]_0/[I]_0 = 100$).

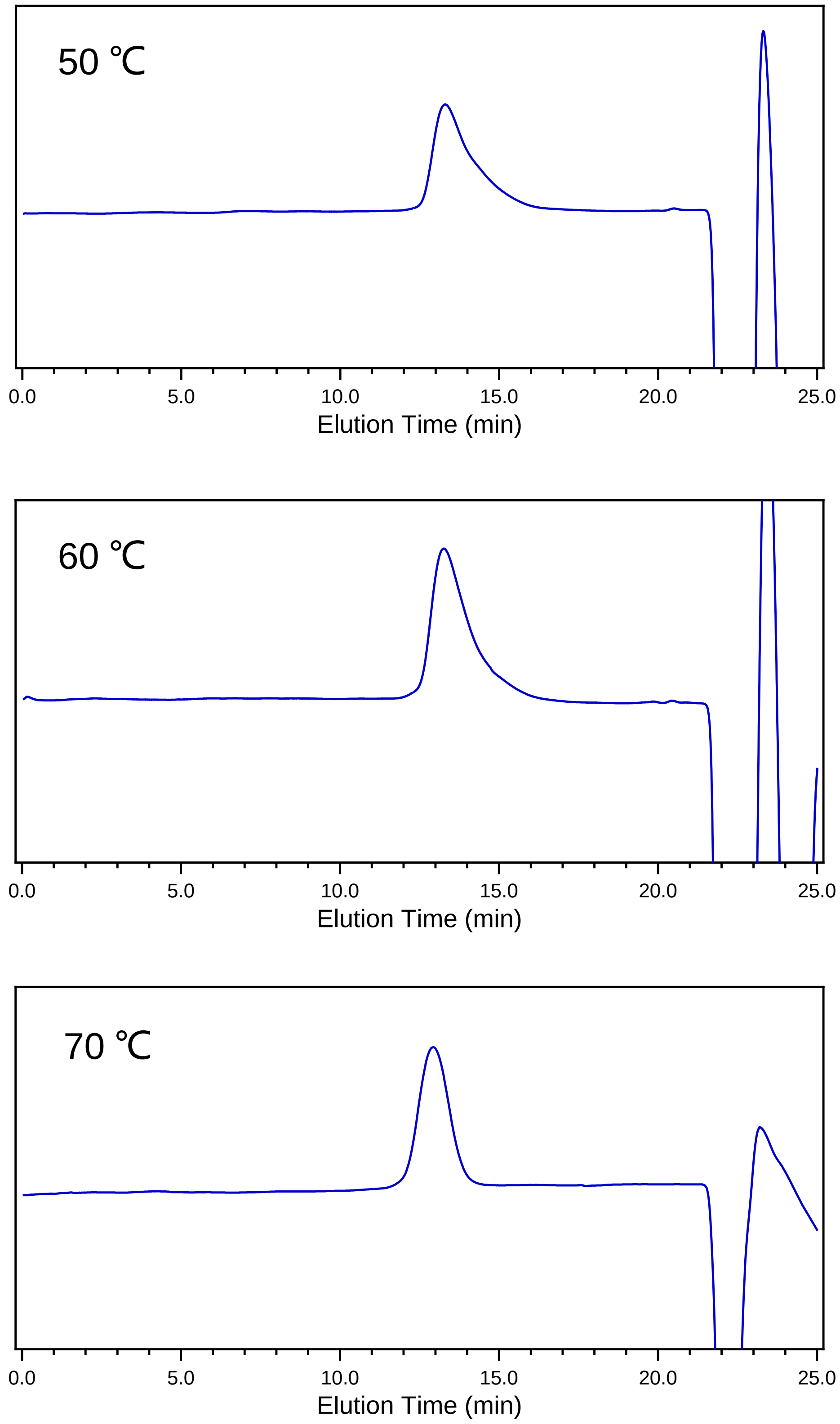

Figure S62. GPC raw data of PNBO synthesized using *n*-$BuNH_3^+Cl^-$ initiator at varying temperatures (50 ^oC, 60 ^oC, and 70 ^oC; $[M]_0/[I]_0 = 100$).

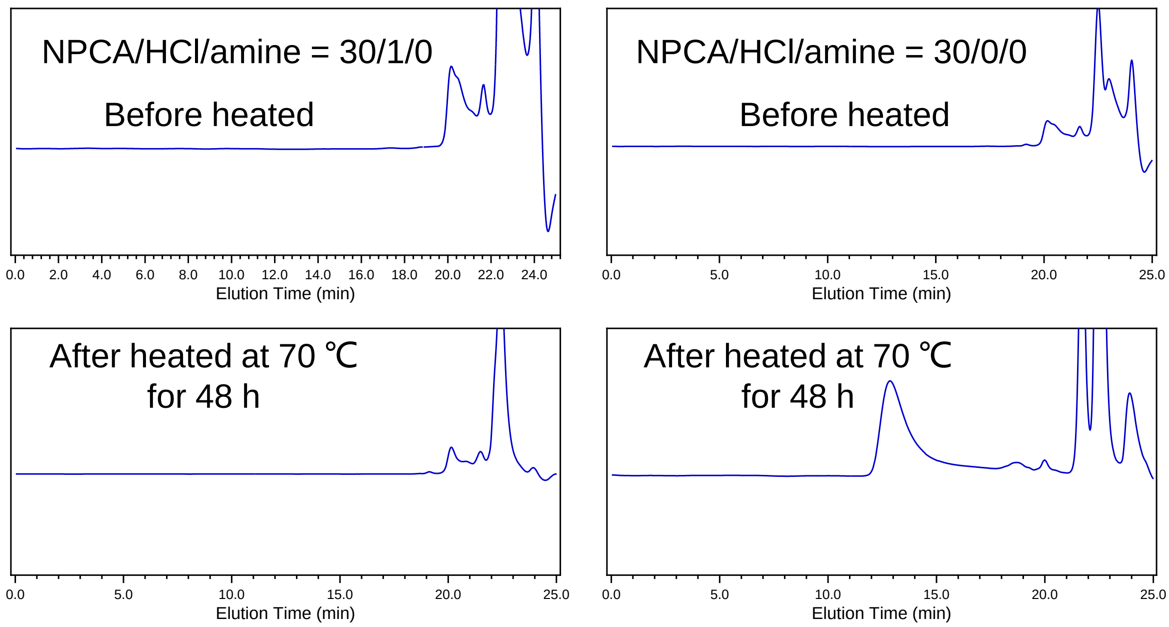

Figure S63. GPC raw data of comparing CbzK polymerizations at 70 ℃ (a) with and (b) without HCl addition in the absence of amine initiator.

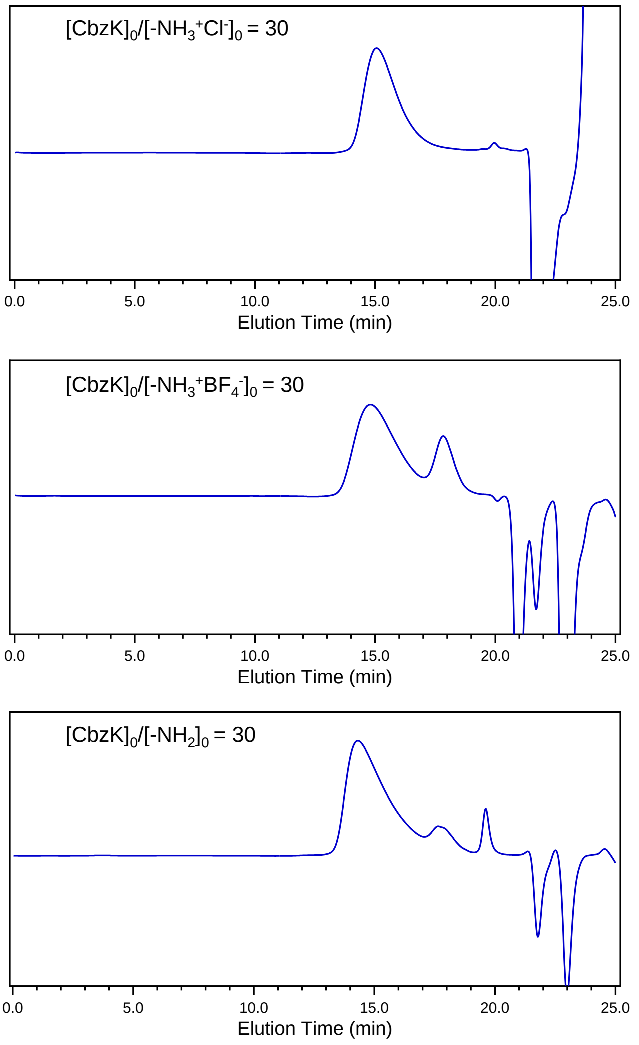

Figure S64. GPC raw data of comparing NPCA polymerizations using (a) *n*-$BuNH_3^+Cl^-$, (b) *n*-$BuNH_3^+BF_4^-$, and (c) *n*-$BuNH_2$ as the initiators ($[M]_0/[I]_0 = 30$).

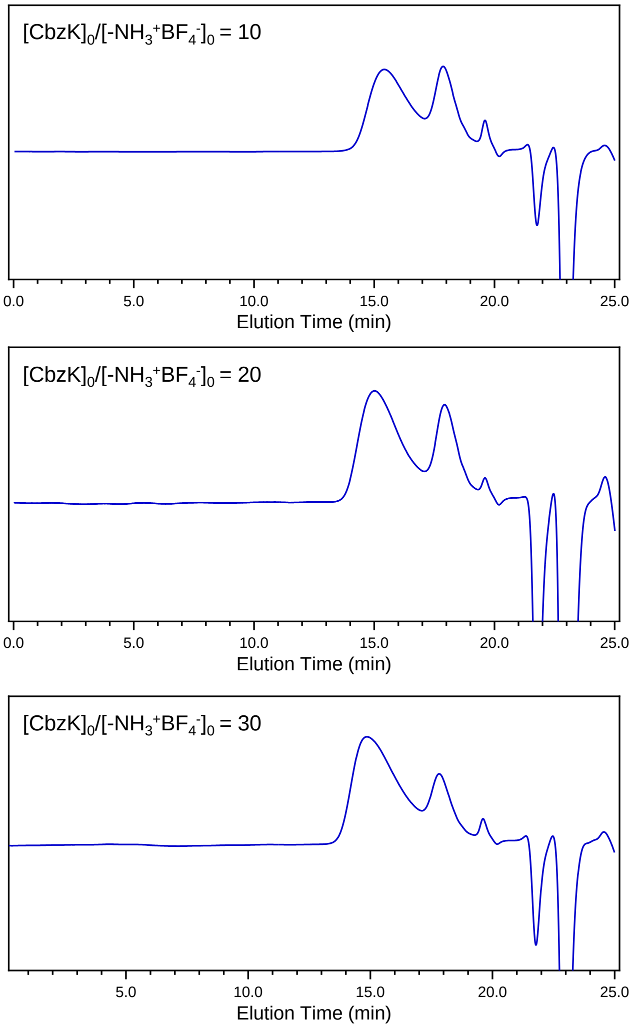

Figure S65. GPC raw data recorded for NPCA polymerization at a feed ratio of 10, 20, and 30 using *n*-$BuNH_3^+BF_4^-$ as the initiators.

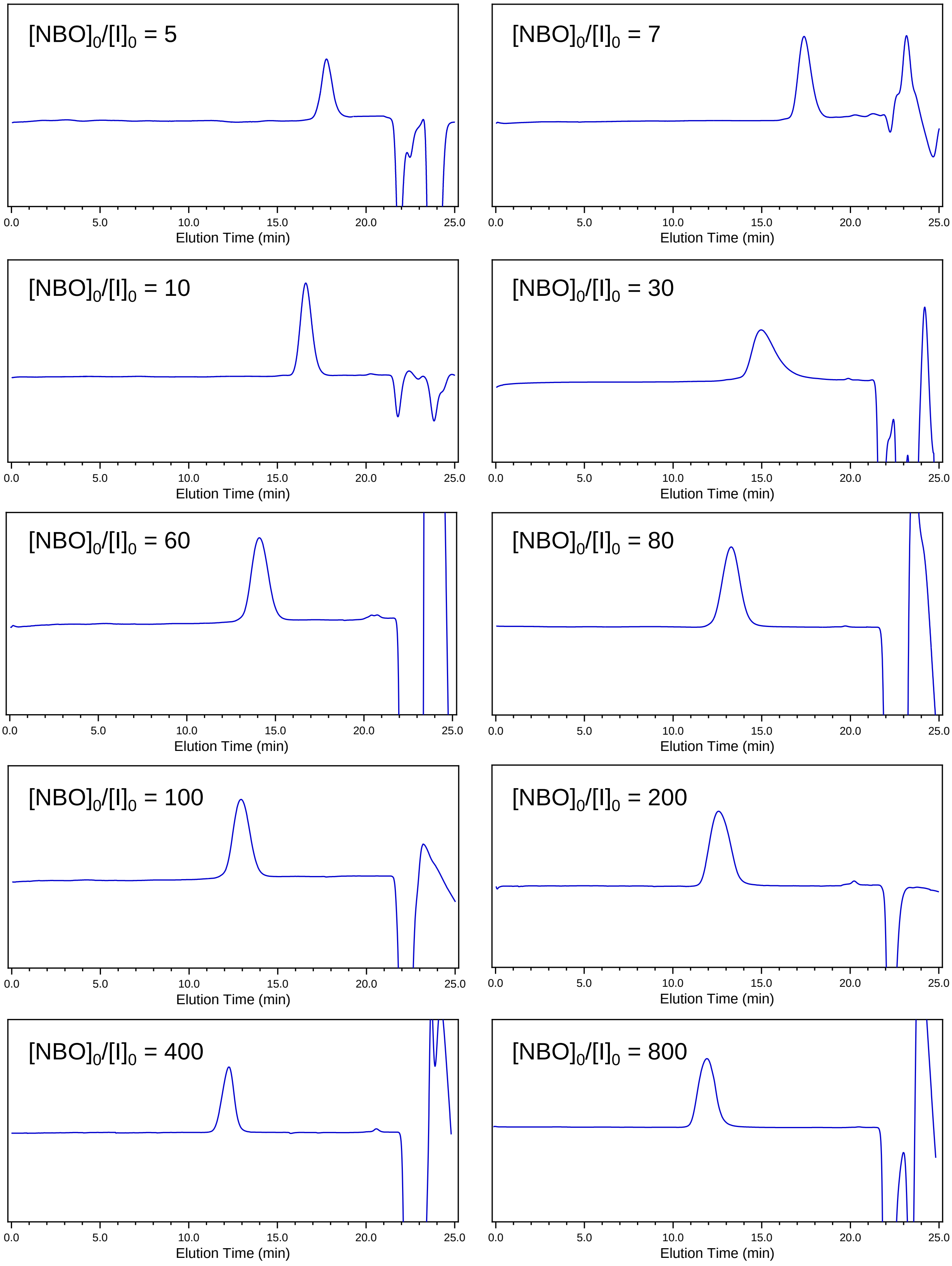

Figure S66. GPC raw data of PNBO polypeptides synthesized at varying $[M]_0/[I]_0$ ratios using *n*-$BuNH_3^+Cl^-$ as initiator.

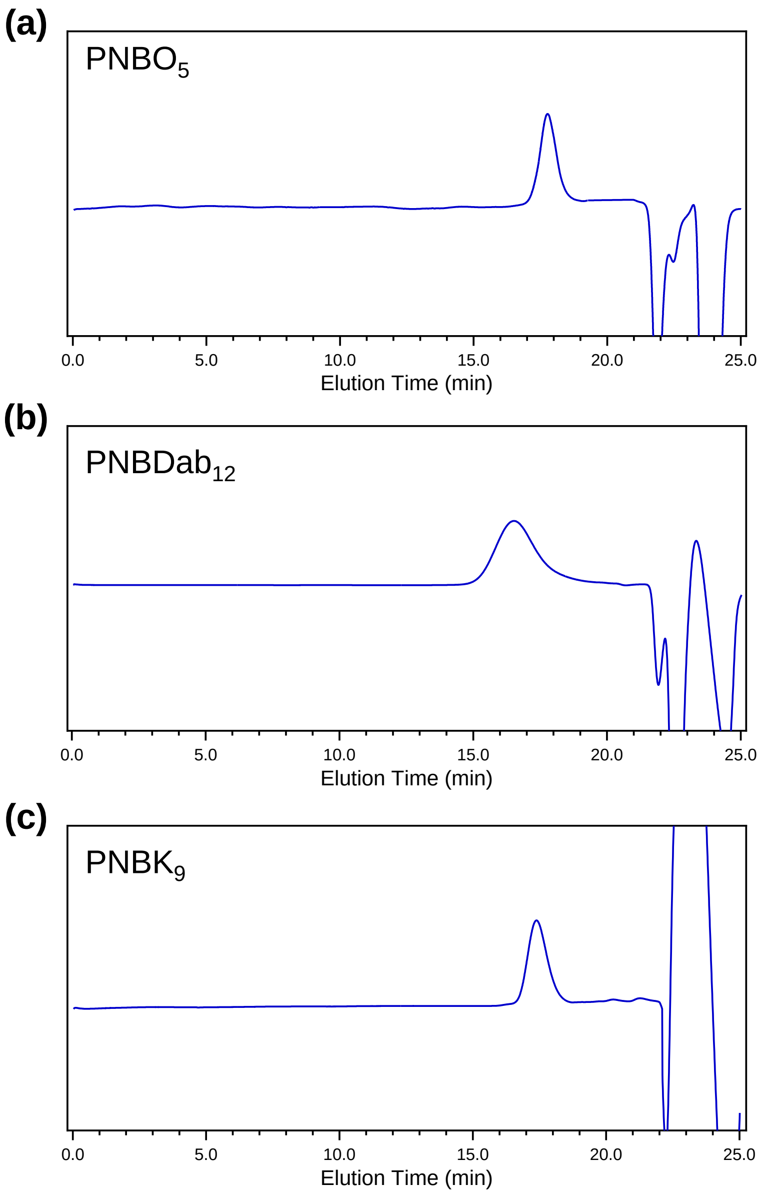

Figure S67. GPC raw data of $PNBO_5$, $PNBDab_{12}$, and $PNBK_9$ for MALDI-TOF MS characterization via polymerization of NPCA precursor ($[M]_0$ = 0.25 M, DMAc, 70 ^oC) using n-$BuNH_3^+Cl^-$ as the initiator.

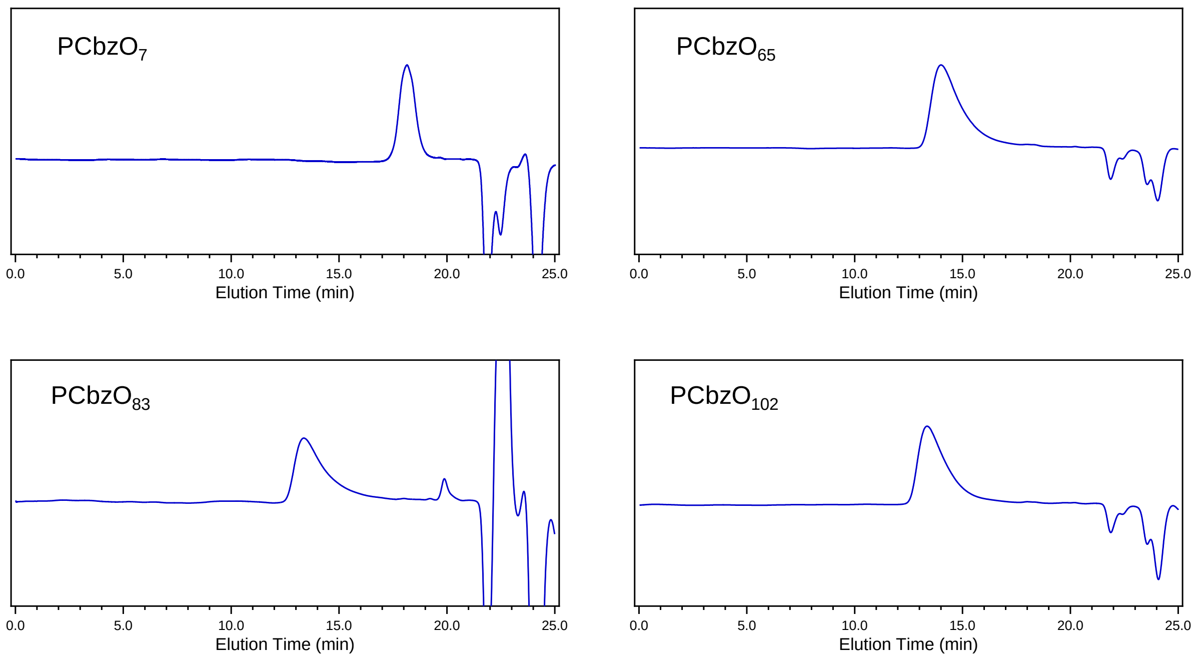

Figure S68. GPC raw data of PCbzO polypeptides synthesized at varying $[M]_0/[I]_0$ ratios using *n*-$BuNH_3^+Cl^-$ as initiator.

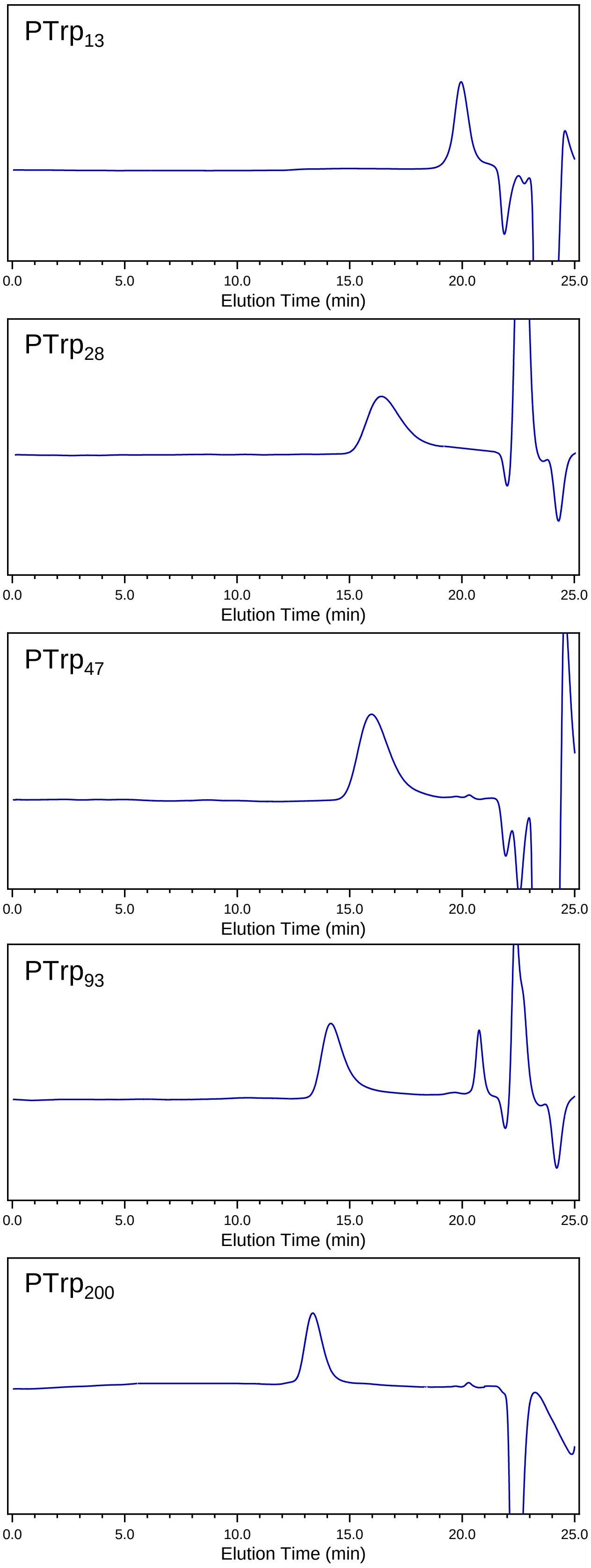

Figure S69. GPC raw data of PTrp polypeptides synthesized at varying $[M]_0/[I]_0$ feed ratios using *n*-$BuNH_3^+Cl^-$ as the initiator.

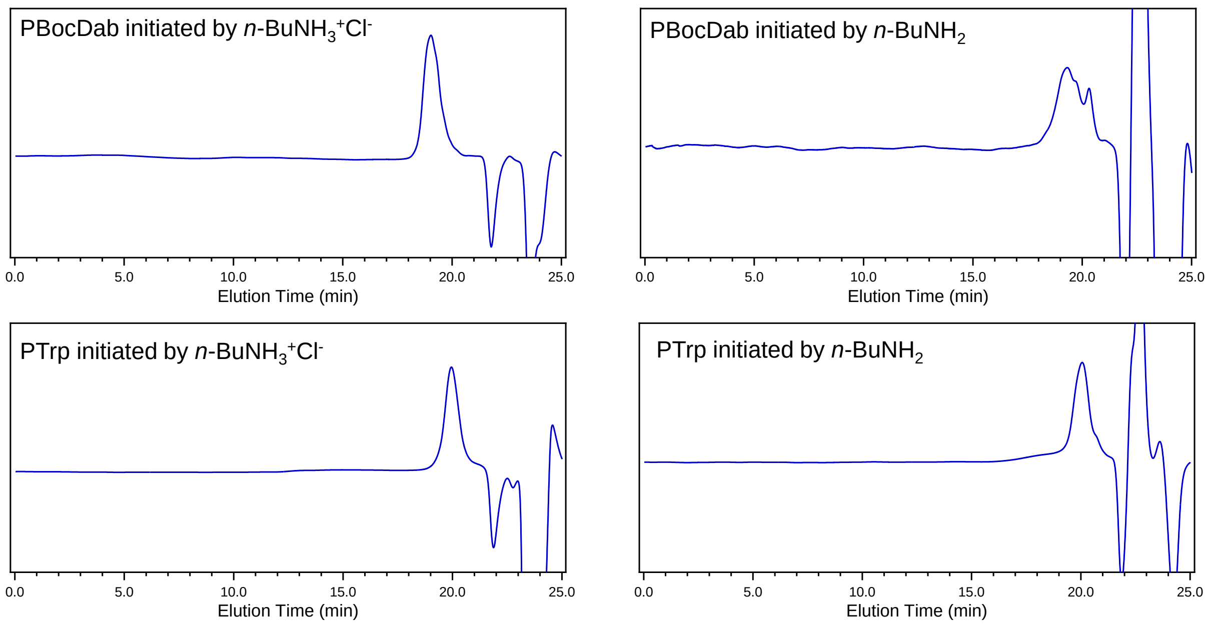

Figure S70. GPC raw data of polypeptides, PBocDab ($[M]_0/[I]_0$ =5) and PTrp ($[M]_0/[I]_0$ = 14) synthesized via NPCA polymerization using *n*-$BuNH_3^+Cl^-$ and *n*-$BuNH_2$ as initiators, respectively.

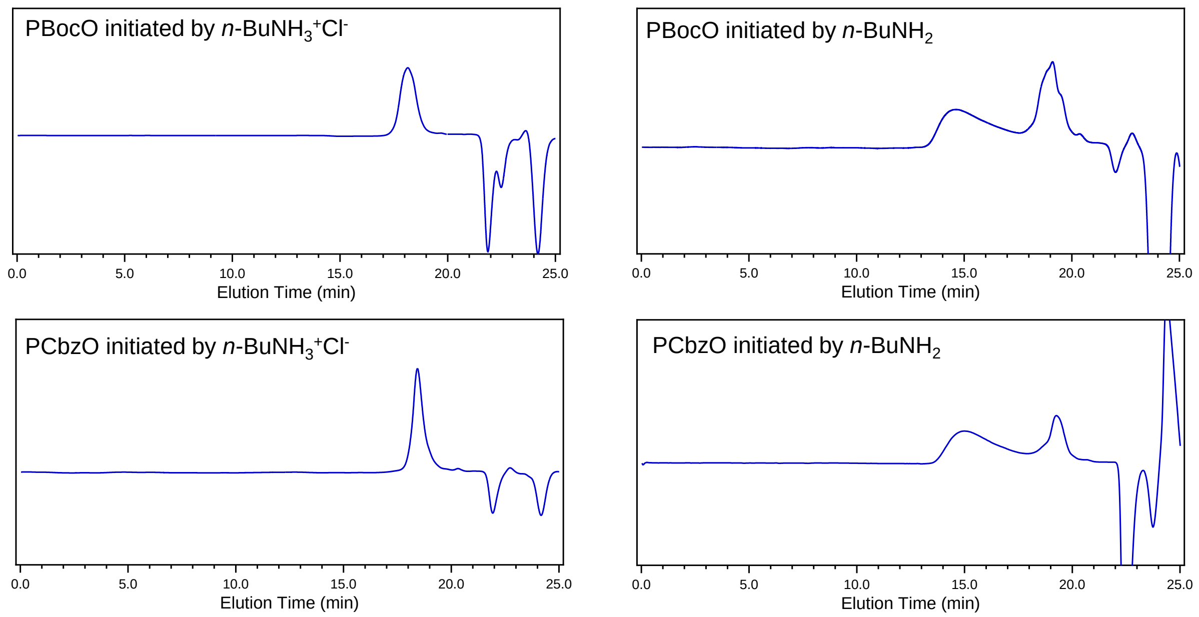

Figure S71. GPC raw data of polypeptides, PBocO ($[M]_0/[I]_0 = 7$) and PCbzO ($[M]_0/[I]_0 = 7$), synthesized via NPCA polymerization using *n*-$BuNH_3^+Cl^-$ and *n*-$BuNH_2$ as initiators, respectively.

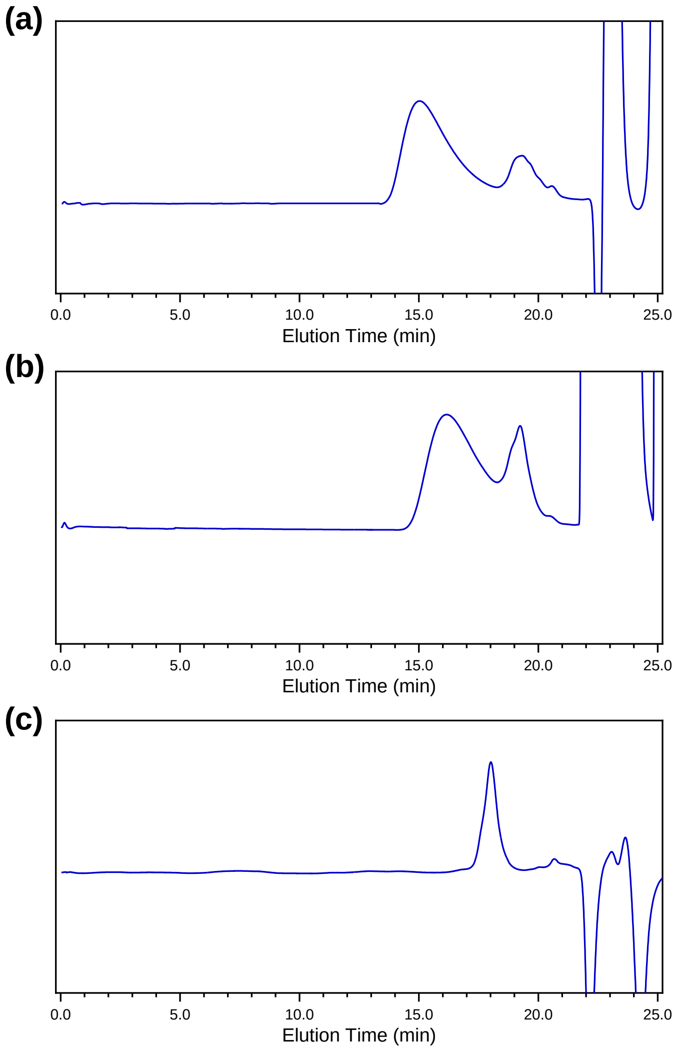

Figure S72. GPC raw data of PCbzK initiated by (a) *n*-$BuNH_2$ ($[M]_0/[I] = 10/1$), (b) *n*-$BuNH_2$ with the addition of acetic acid ($[M]_0/[AA]/[I] = 10/10/1$), and (c) *n*-$BuNH_3^+Cl^-$ ($[M]_0/[I] = 10/1$) via polymerization of CbzK NPCA precursor using different initiators.

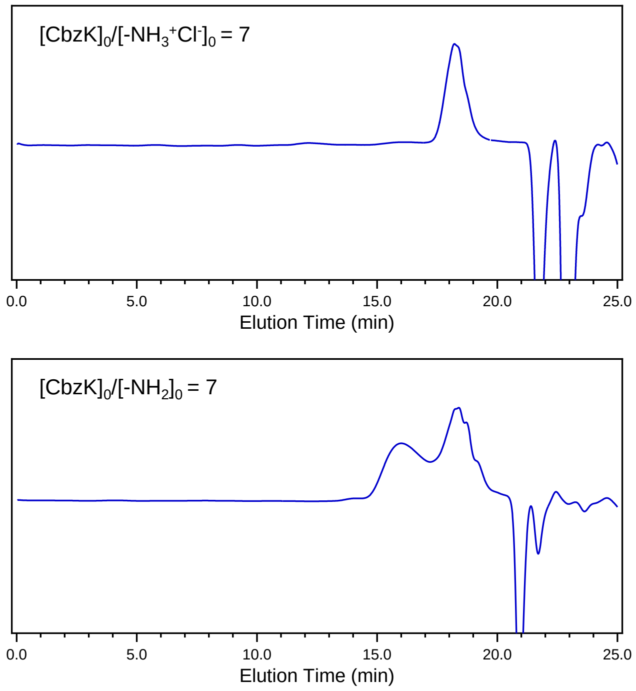

Figure S73. GPC raw data recorded for polymerization comparison of NPCA (CbzK) at a feed ratio of 7 using *n*-$BuNH_3^+Cl^-$ and *n*-$BuNH_2$ as the initiators.

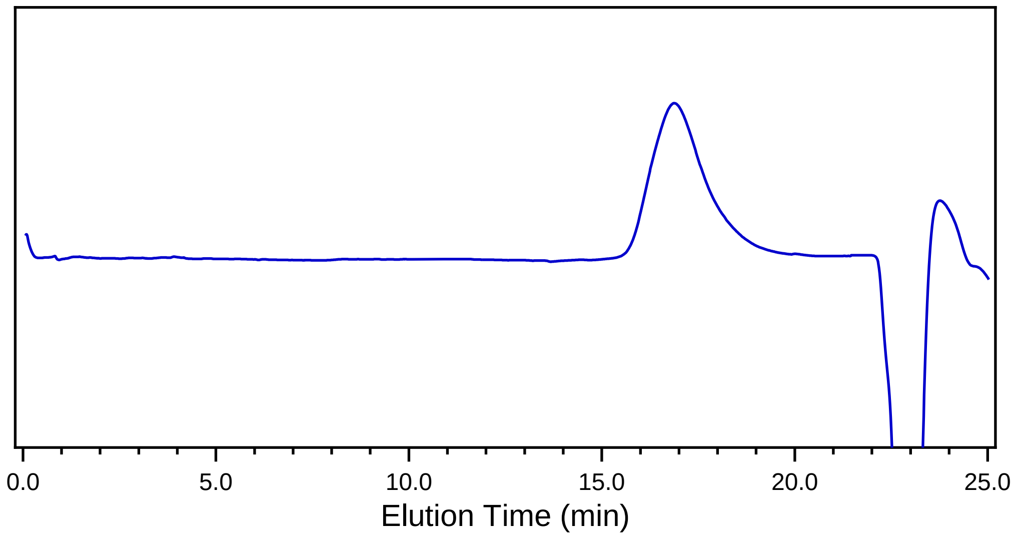

Figure S74. GPC raw data recorded for the obtained $PNBO_{10}$ for the analysis of H_2O content of DMAc in open-vessel polymerization ($[M]_0$ = 0.25 M, DMAc, 70 ^oC).

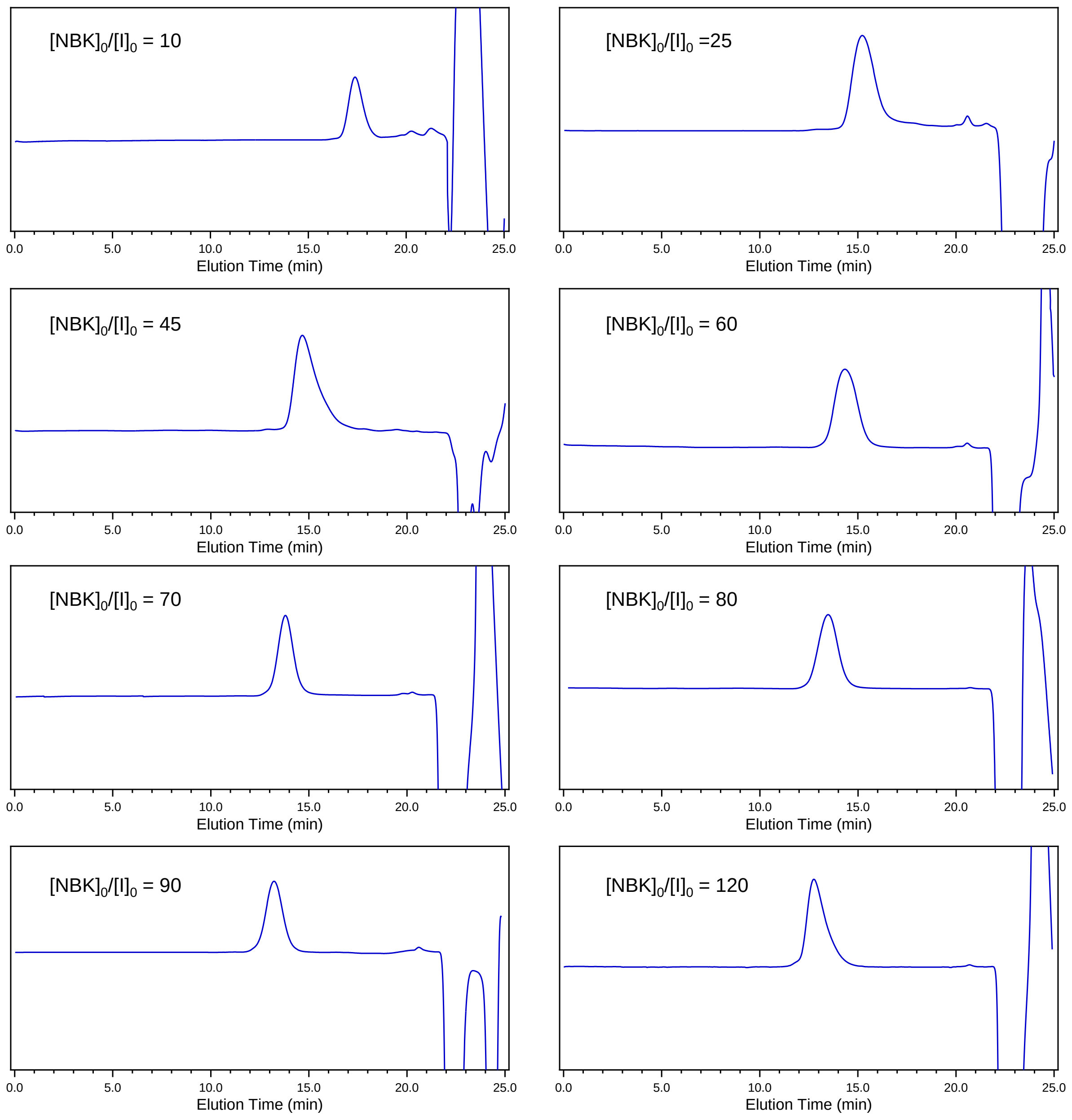

Figure S75. GPC raw data of PNBK polypeptides synthesized at varying $[M]_0/[I]_0$ ratios using *n*-$BuNH_3^+Cl^-$ as initiator under open vessel.

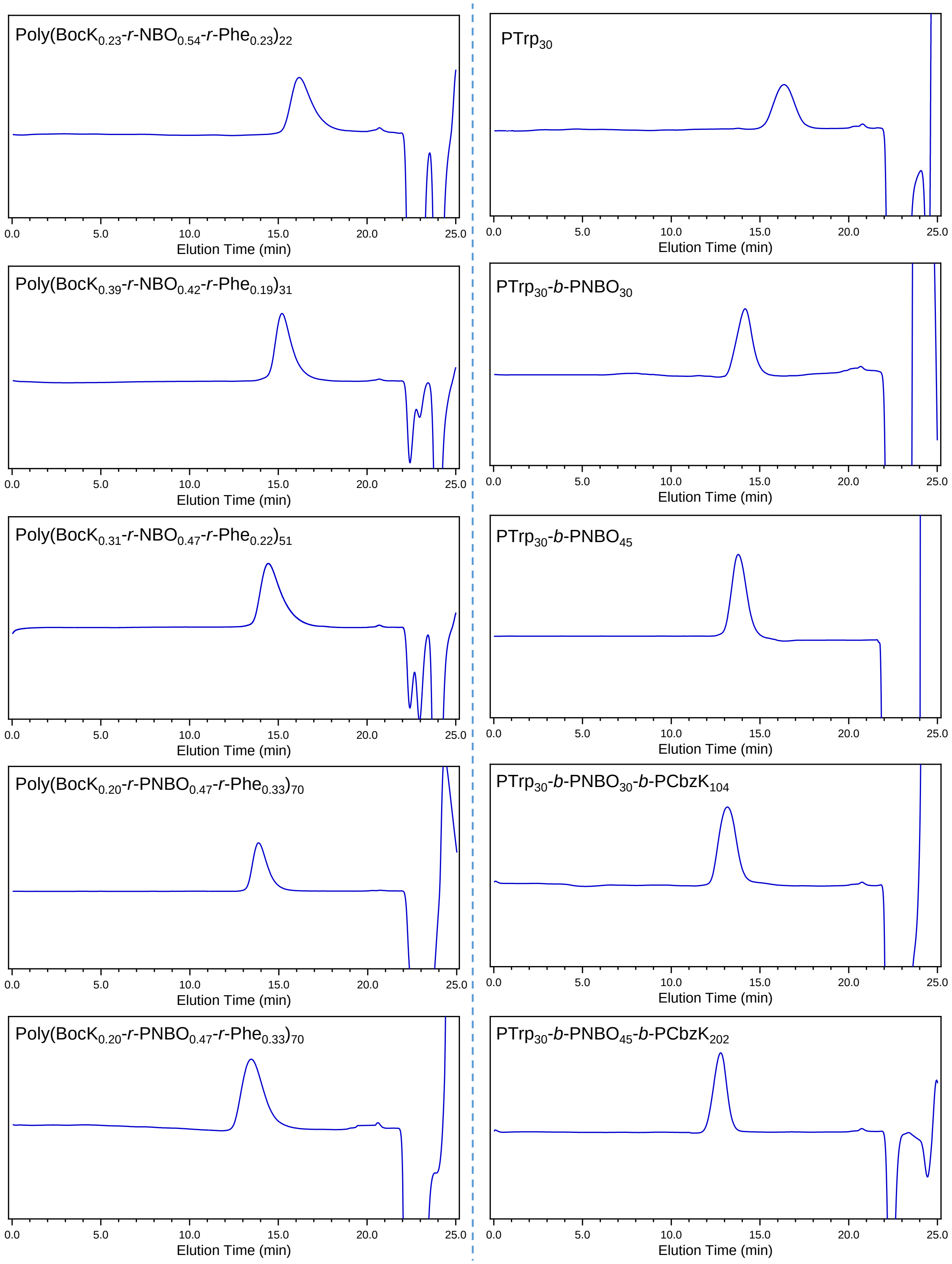

Figure S76. GPC raw data of random and block copolypeptides synthesized at varying $[M]_0/[I]_0$ feed ratios using *n*-$BuNH_3^+Cl^-$ as the initiator under open vessel.

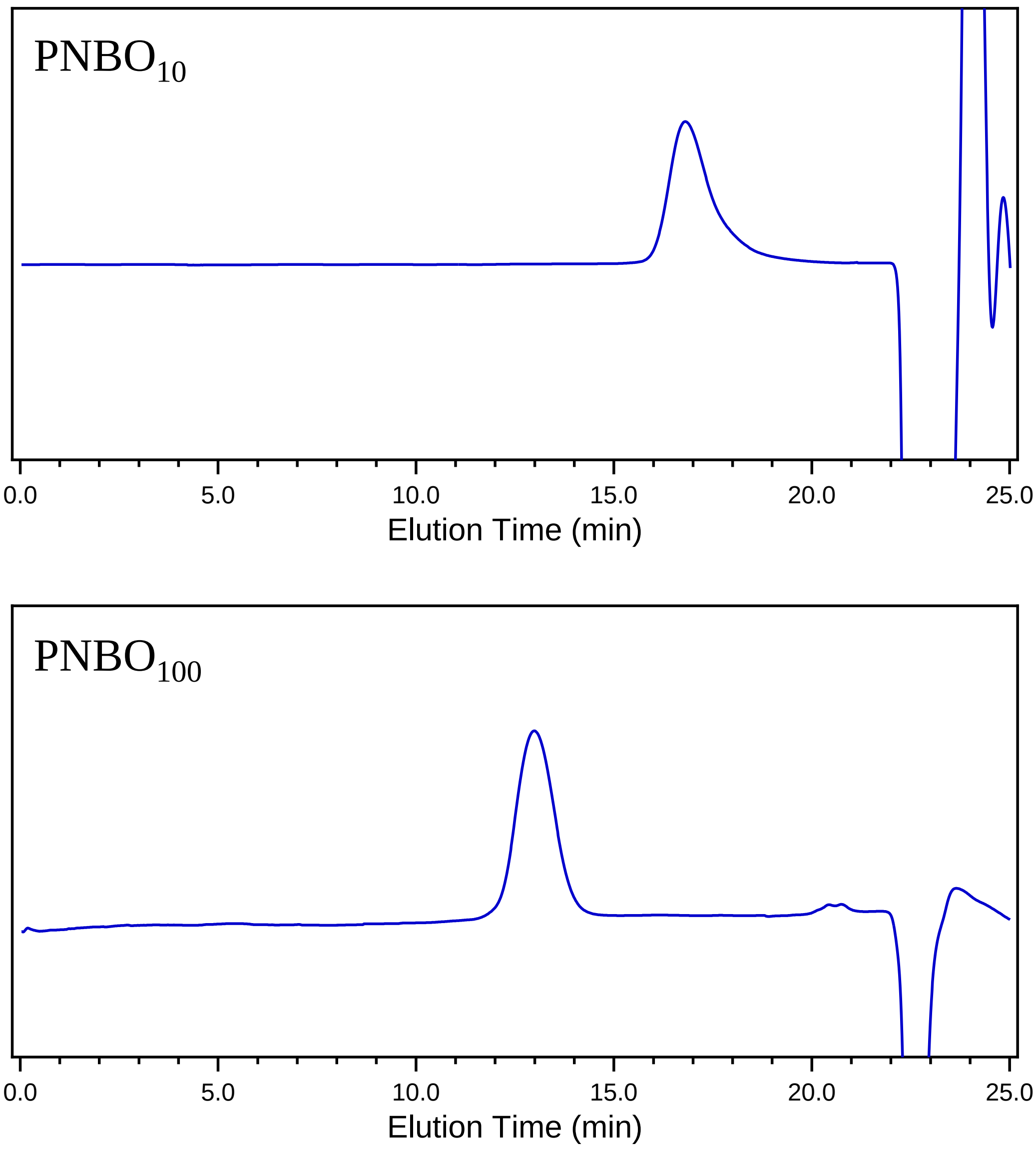

Figure S77. GPC raw data of $PNBO_{10}$ and $PNBO_{100}$ via polymerization of NPCA precursor at gram scale in open vessel initiated by *n*-$BuNH_3^+Cl^-$, $[M]_0$ = 0.25 M, DMAc, 70 °C.

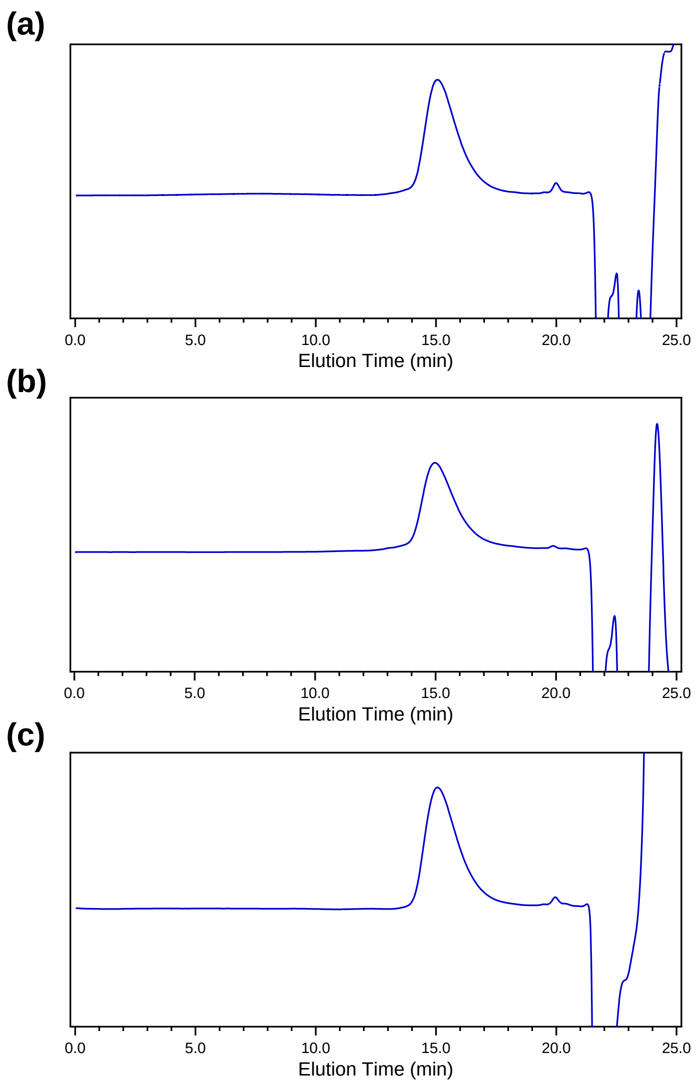

Figure S78. GPC raw data recorded for PCbzK polypeptide synthesized under different conditions ($[M]_0/[I]_0 = 30$): (a) inside glove box, tens of milligram scale, (b) open-vessel, tens of milligram scale, (c) open-vessel, gram scale. All polymerizations were conducted at $[M]_0$ = 0.25 M in DMAc and 70 ^{o}C.
